# Cryo-EM structures of Nipah virus polymerase complex reveal highly varied interactions between L and P proteins among paramyxoviruses

**DOI:** 10.1093/procel/pwaf014

**Published:** 2025-02-18

**Authors:** Lu Xue, Tiancai Chang, Jiacheng Gui, Zimu Li, Heyu Zhao, Binqian Zou, Junnan Lu, Mei Li, Xin Wen, Shenghua Gao, Peng Zhan, Lijun Rong, Liqiang Feng, Peng Gong, Jun He, Xinwen Chen, Xiaoli Xiong

**Affiliations:** State Key Laboratory of Respiratory Disease, Guangdong Provincial Key Laboratory of Stem Cell and Regenerative Medicine, GIBH-CUHK Joint Research Laboratory on Stem Cell and Regenerative Medicine, Guangzhou Institutes of Biomedicine and Health, Chinese Academy of Sciences, Guangzhou 510530, China; State Key Laboratory of Respiratory Disease, Guangdong Provincial Key Laboratory of Stem Cell and Regenerative Medicine, GIBH-CUHK Joint Research Laboratory on Stem Cell and Regenerative Medicine, Guangzhou Institutes of Biomedicine and Health, Chinese Academy of Sciences, Guangzhou 510530, China; Graduate School of Guangzhou Medical University, Guangzhou Medical University-Guangzhou Institutes of Biomedicine and Health Joint School of Life Sciences, Guangzhou Medical University, Guangzhou 511436, China; State Key Laboratory of Respiratory Disease, Guangdong Provincial Key Laboratory of Stem Cell and Regenerative Medicine, GIBH-CUHK Joint Research Laboratory on Stem Cell and Regenerative Medicine, Guangzhou Institutes of Biomedicine and Health, Chinese Academy of Sciences, Guangzhou 510530, China; Medical School, University of Chinese Academy of Sciences, Beijing 100864, China; State Key Laboratory of Respiratory Disease, Guangdong Provincial Key Laboratory of Stem Cell and Regenerative Medicine, GIBH-CUHK Joint Research Laboratory on Stem Cell and Regenerative Medicine, Guangzhou Institutes of Biomedicine and Health, Chinese Academy of Sciences, Guangzhou 510530, China; Graduate School of Guangzhou Medical University, Guangzhou Medical University-Guangzhou Institutes of Biomedicine and Health Joint School of Life Sciences, Guangzhou Medical University, Guangzhou 511436, China; Guangzhou National Laboratory, Guangzhou 510005, China; State Key Laboratory of Respiratory Disease, Guangdong Provincial Key Laboratory of Stem Cell and Regenerative Medicine, GIBH-CUHK Joint Research Laboratory on Stem Cell and Regenerative Medicine, Guangzhou Institutes of Biomedicine and Health, Chinese Academy of Sciences, Guangzhou 510530, China; Medical School, University of Chinese Academy of Sciences, Beijing 100864, China; State Key Laboratory of Respiratory Disease, Guangdong Provincial Key Laboratory of Stem Cell and Regenerative Medicine, GIBH-CUHK Joint Research Laboratory on Stem Cell and Regenerative Medicine, Guangzhou Institutes of Biomedicine and Health, Chinese Academy of Sciences, Guangzhou 510530, China; Graduate School of Guangzhou Medical University, Guangzhou Medical University-Guangzhou Institutes of Biomedicine and Health Joint School of Life Sciences, Guangzhou Medical University, Guangzhou 511436, China; State Key Laboratory of Respiratory Disease, Guangdong Provincial Key Laboratory of Stem Cell and Regenerative Medicine, GIBH-CUHK Joint Research Laboratory on Stem Cell and Regenerative Medicine, Guangzhou Institutes of Biomedicine and Health, Chinese Academy of Sciences, Guangzhou 510530, China; Guangzhou National Laboratory, Guangzhou 510005, China; Medical School, University of Chinese Academy of Sciences, Beijing 100864, China; Key Laboratory of Special Pathogens and Biosafety, Wuhan Institute of Virology, Center for Biosafety Mega-Science, Chinese Academy of Sciences, Wuhan 430061, China; Department of Medicinal Chemistry, Key Laboratory of Chemical Biology (Ministry of Education), School of Pharmaceutical Sciences, Shandong University, Jinan 250013, China; Department of Medicinal Chemistry, Key Laboratory of Chemical Biology (Ministry of Education), School of Pharmaceutical Sciences, Shandong University, Jinan 250013, China; Departments of Microbiology and Immunology, College of Medicine, University of Illinois Chicago, Chicago, IL 60612, USA; State Key Laboratory of Respiratory Disease, Guangdong Provincial Key Laboratory of Stem Cell and Regenerative Medicine, GIBH-CUHK Joint Research Laboratory on Stem Cell and Regenerative Medicine, Guangzhou Institutes of Biomedicine and Health, Chinese Academy of Sciences, Guangzhou 510530, China; Key Laboratory of Special Pathogens and Biosafety, Wuhan Institute of Virology, Center for Biosafety Mega-Science, Chinese Academy of Sciences, Wuhan 430061, China; State Key Laboratory of Respiratory Disease, Guangdong Provincial Key Laboratory of Stem Cell and Regenerative Medicine, GIBH-CUHK Joint Research Laboratory on Stem Cell and Regenerative Medicine, Guangzhou Institutes of Biomedicine and Health, Chinese Academy of Sciences, Guangzhou 510530, China; Guangzhou National Laboratory, Guangzhou 510005, China; Key Laboratory of Special Pathogens and Biosafety, Wuhan Institute of Virology, Center for Biosafety Mega-Science, Chinese Academy of Sciences, Wuhan 430061, China; State Key Laboratory of Respiratory Disease, Guangdong Provincial Key Laboratory of Stem Cell and Regenerative Medicine, GIBH-CUHK Joint Research Laboratory on Stem Cell and Regenerative Medicine, Guangzhou Institutes of Biomedicine and Health, Chinese Academy of Sciences, Guangzhou 510530, China

**Keywords:** Nipah virus, Paramyxovirus, RNA-dependent RNA polymerase (RdRp), L-P polymerase complex, Cryo-EM

## Abstract

Nipah virus (NiV) and related viruses form a distinct *henipavirus* genus within the *Paramyxoviridae* family. NiV continues to spillover into the humans causing deadly outbreaks with increasing human–bat interaction. NiV encodes the large protein (L) and phosphoprotein (P) to form the viral RNA polymerase machinery. Their sequences show limited homologies to those of non-*henipavirus* paramyxoviruses. We report two cryo-electron microscopy (cryo-EM) structures of the Nipah virus (NiV) polymerase L-P complex, expressed and purified in either its full-length or truncated form. The structures resolve the RNA-dependent RNA polymerase (RdRp) and polyribonucleotidyl transferase (PRNTase) domains of the L protein, as well as a tetrameric P protein bundle bound to the L-RdRp domain. L-protein C-terminal regions are unresolved, indicating flexibility. Two PRNTase domain zinc-binding sites, conserved in most *Mononegavirales*, are confirmed essential for NiV polymerase activity. The structures further reveal anchoring of the P protein bundle and P protein X domain (XD) linkers on L, via an interaction pattern distinct among *Paramyxoviridae*. These interactions facilitate binding of a P protein XD linker in the nucleotide entry channel and distinct positioning of other XD linkers. We show that the disruption of the L–P interactions reduces NiV polymerase activity. The reported structures should facilitate rational antiviral-drug discovery and provide a guide for the functional study of NiV polymerase.

## Introduction

Nipah virus (NiV) is a zoonotic virus with a human fatality rate of 40%–70%. The natural hosts of NiV are fruit bats of the *Pteropus* genus, whose habitat spans Southeast Africa, India, Southeast Asia, as well as Northern and Western Australia (Rahman et al., 2010). These bats often inhabit areas close to dense human populations or livestock, facilitating the zoonotic transmission of the virus. NiV was first identified in Malaysia in 1998 with the outbreak lasting until May 1999, infecting 265 people and causing encephalitis with a fatality rate of ~45% (Chua et al., 2000; Enserink, 1999). During this outbreak, NiV primarily spread from bats to domestic pigs and subsequently to humans. However, outbreaks in India and Bangladesh in 2001 (Chadha et al., 2006), and subsequent years in Bangladesh, showed a different transmission pattern, with NiV spreading directly from bats to humans and further engaging in human-to-human transmission (Gurley et al., 2007; Hsu et al., 2004). Recently, in August 2023, NiV resurfaced in Kerala, South India, with two reported fatalities among six infected individuals. Increasing human activities in or near forests, along with climate change, have increased the overlap between human and bat habitats, elevating the risk of NiV transmission. NiV is classified as a biosafety level 4 (BSL-4) pathogen and has been identified by the World Health Organization as a priority infectious disease warranting urgent research. Unfortunately, no vaccine or other treatments have been currently approved for NiV.

NiV is a nonsegmented negative-sense (NNS) RNA virus belonging to the genus *Henipavirus* in the family *Paramyxoviridae*, order *Mononegavirales*. The NiV genome is approximately 18k nts in length (Harcourt et al., 2005), primarily encoding six structural proteins: nucleoprotein (N), phosphoprotein (P), matrix (M) protein, fusion (F) protein, glycoprotein (G), and large (L) protein. Due to the longer P gene and extended UTR regions flanking the L gene, NiV has a longer genome compared to other paramyxoviruses. Within the core of the viral particle, the genome is tightly encapsidated by multiple copies of the nucleoprotein (N), forming a ribonucleoprotein (RNP) complex. The RNP complex functions as an essential component in viral genome replication and transcription. The L protein, as the largest viral protein, has a molecular weight of approximately 250 kDa (Jordan et al., 2018). It comprises five domains (Liang, 2020), including the RNA-dependent RNA polymerase (RdRp) domain located at the N-terminus, which serves as the polymerase catalytic core, followed by the poly-ribonucleotidyltransferase (PRNTase) domain, which functions as the second enzymatic domain, primarily mediating mRNA capping. The remaining domains, the connector domain (CD), the methyltransferase (MTase) domain, and the C-terminal domain (CTD), collectively constitute the C-terminus of the L protein. The third enzymatic domain, MTase domain, is primarily responsible for methylating the cap structure at the 2'-O and N7 positions in the viral mRNA.

The P protein is multi-functional and serves as an essential cofactor in the RNA synthesis of non-segmented negative-sense (NNS) RNA viruses through its interactions with the L protein. It also regulates the assembly of nascent monomeric N protein (N^0^). The P protein primarily comprises three domains: the N-terminal domain (P_NTD_), the central oligomerization domain (P_OD_) and the C-terminal X domain (P_XD_) (Bruhn et al., 2014; Jensen et al., 2020). The P_NTD_ is largely disordered and mainly responsible for binding with N^0^, preventing its nonspecific interaction with host RNA, and promoting the assembly of N^0^ on nascent viral RNA (Yabukarski et al., 2014). In addition, the P_NTD_ also binds STAT1 (Signal Transducer and Activator of Transcription 1), thereby blocking interferon (IFN) signaling (Ciancanelli et al., 2009; Devaux et al., 2007, 2013; Shaw et al., 2004). The P_XD_ presumably functions to facilitate the entry of the RNA template and NTP into their respective channels for replication and transcription (Pan et al., 2020).

To date, several polymerases of *Mononegavirales* have been structurally characterized. These include polymerases of Ebola virus (EBOV) (Peng et al., 2023; Yuan et al., 2022) from the *Filoviridae* family, human respiratory syncytial virus (RSV) (Cao et al., 2020, 2024; Gilman et al., 2019) and human metapneumovirus (hMPV) (Pan et al., 2020) from the *Pneumoviridae* family, vesicular stomatitis virus (VSV) (Jenni et al., 2020; Liang et al., 2015) and rabies virus (RABV) (Horwitz et al., 2020) from the *Rhabdoviridae* family, and parainfluenza virus 3 (PIV3) (Xie et al., 2024), parainfluenza virus 5 (PIV5) (Abdella et al., 2020), Newcastle disease virus (NDV) (Cong et al., 2023), and mumps virus (MuV) (Li et al., 2024) from the *Paramyxoviridae* family. Their structures reveal that a general similarity in the overall three-dimensional architecture. Although crystal structures of the NiV P protein have been reported (Bruhn et al., 2014, 2019), the structure of the NiV L protein and its interactions with the P proteins remain elusive.

Here, we report two structures of the NiV L-P complexes in an apo state using cryo-electron microscopy (cryo-EM). The structures reveal metal sites essential for polymerase activity. We report that tetrameric P proteins are anchored on the Nipah L protein RdRp domain surface distinctively among paramyxoviruses. We show that L–P interface mutations affect polymerase mini-replicon activity. The newly presented structural and biochemical data provide a foundation for computer-aided docking of small molecules to the NiV polymerase structure, facilitating the discovery of antivirals that target RNA synthesis mediated by the NiV L-P complex.

## Results

### Structure determination of the NiV polymerase complexes

**Figure 1.**
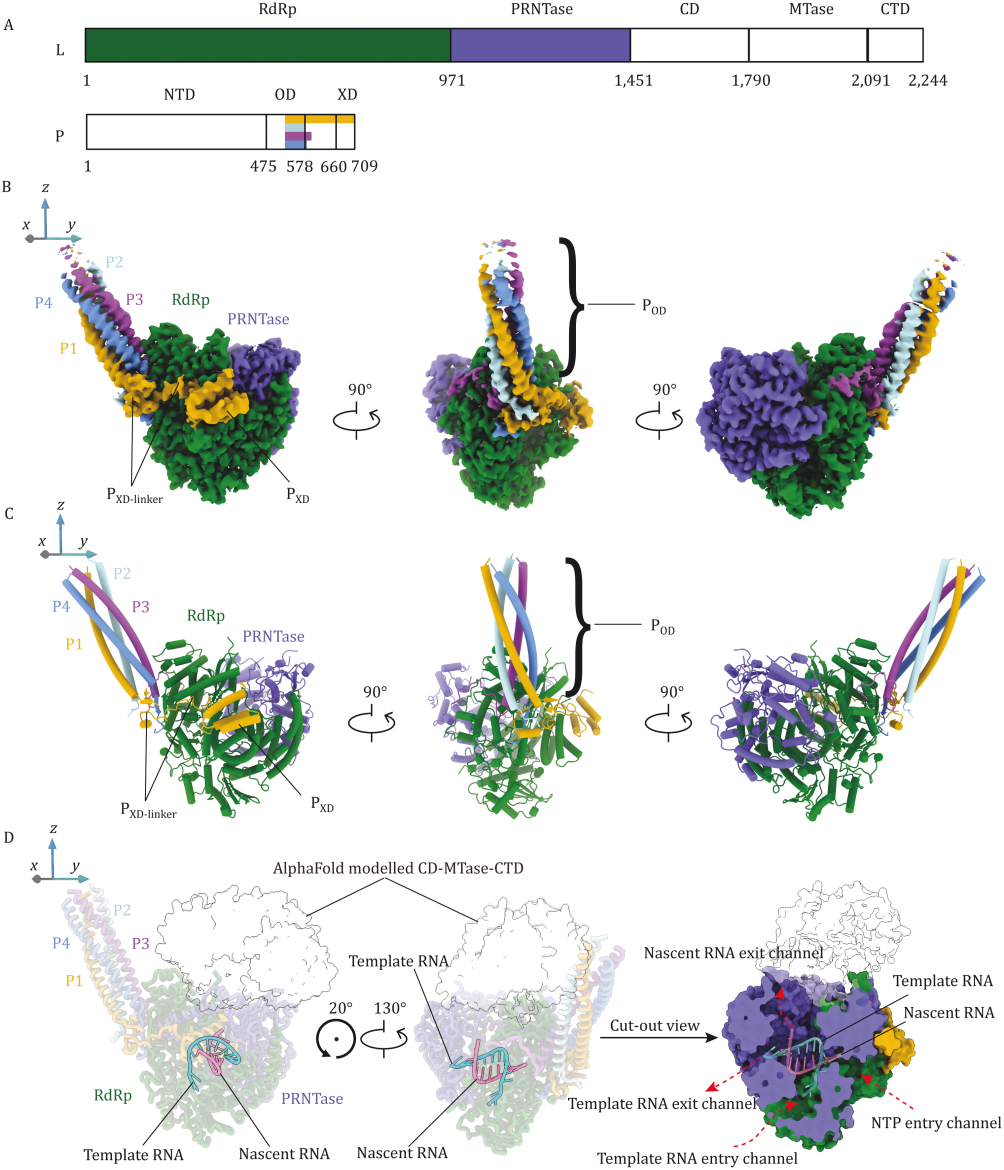
**Overall structure of the NiV polymerase complex.** (A) Schematic representation of the domain organizations in NiV L and P. RdRp and PRNTase domains are colored green and purple. Monomers in the P protein bundle is colored as follows: P1, yellow; P2, cyan; P3, magenta; P4, blue. The color scheme for each component of the NiV L and P is used in panels (B) and (C). (B and **C)** Three different views of the density map (B) and atomic model (C) of the NiV L_1__–__1,451_-P complex. (D) A model of the NiV L-P elongation complex. The template-nacsent RNA duplex was modeled based on a superposition of the Thogoto virus polymerase in a replication elongation-reception conformation (PDB: 8Z9R). The structures of CD, MTase and CTD domains of NiV L protein were predicted by AlphaFold2 and shown as transparency surfaces. Template and nascent RNA strands are colored green and pink.

To determine the NiV polymerase complex structure, we co-expressed a truncated version of NiV L (aa 1–1,451) protein and full-length NiV P protein in Sf9 insect cells using a bac-to-bac expression system. Size-exclusion chromatography of purified L_1–1,451_-P complex was confirmed to contain the L_1__–__1,451_ (~170 kDa) and P (~80 kDa) proteins with the expected sizes by SDS-PAGE (**Fig. S1A**). This NiV L_1–1,451_-P complex was imaged by cryo-EM. Particles picked from 13,033 micrographs were subjected to 2D classification followed by ab-initio reconstruction using cryoSPARC (Punjani et al., 2017), achieving a final structure with an overall resolution of 2.3 Å (**Fig. S2**; **Table S1**). The cryo-EM density map reveals that the 3D structure of the NiV polymerase complex is similar to those of other NNS RNA virus polymerases, such as those of EBOV (Peng et al., 2023; Yuan et al., 2022), and RSV (Cao et al., 2020, 2024; Gilman et al., 2019) (**Figs. 1A–C** and **S2**). In the structure we obtained, two domains are resolved for the L protein: the RdRp (RNA-dependent RNA polymerase, aa 1–971) domain and the PRNTase (polyribonucleotidyl transferase, aa 972–1,451) domain (**Fig. 1A–C**). For the P protein, which forms a homo-tetramer bound to the L protein, we successfully resolved its P_OD_ domain (oligomerization domain, aa 525–578) in all 4 monomers, forming of a long, tetrameric coiled coil (**Fig. 1A–C**). Additionally, we resolved the P1_XD_ domain (C-terminal X domain, aa 660–709), which is primarily composed of three helices (**Fig. 1A–C**). Finally, various lengths of the P_XD_ linker regions (579–659) among different P monomers are resolved. In the L_1__–__1,451_-P complex, due to the truncation of the L protein, connector domain (CD, aa 1,452–1,789), the methyltransferase domain (MTase, aa 1,790–2,090), and C-terminal domain (CTD, aa 2,091–2,244) are absent from the structure (**Fig. 1A–C**). In the hope of revealing a more complete architecture of the polymerase complex, we also co-expressed the full-length NiV L protein in the presence of P. After extensive sample preparation and data collection optimization, we obtained a 2.5 Å reconstruction of the full-length L-P complex (**Fig. S3**; **Table S1**). However, compared to the NiV L_1__–__1,451_-P complex, no additional L protein density was observed, despite that SDS-PAGE indicated the integrity of the L protein (~250 kDa) (**Fig. S1B**). We speculate that, similar to EBOV (Peng et al., 2023; Yuan et al., 2022), HMPV (Pan et al., 2020) and RSV (Cao et al., 2020, 2024; Gilman et al., 2019), the missing density likely reflects the inherent flexibility of the MTase domain, CD and CTD in NiV polymerase complex. As both structures are essentially the same (RMSD = 0.241 Å, 1,339 vs. 1,339 Cα atoms), we describe the NiV polymerase complex structural features primarily based on the L_1__–__1,451_-P complex structure determined to a higher resolution.

### Structure of the NiV L protein

The NiV L protein RdRp domain adopts a right-handed “fingers-palm-thumb” configuration, similar to other RNA virus polymerases (Abdella et al., 2020; Cao et al., 2020; Cong et al., 2023; Gilman et al., 2019; Hillen et al., 2020; Li et al., 2024; Liang et al., 2015; Pan et al., 2020; Pflug et al., 2014; Xie et al., 2024; Xue et al., 2024; Yuan et al., 2022) (**Fig. 1A–C**). The catalytic center also contains a highly conserved motif (G^831^-D^832^-N^833^) in *Mononegavirales*. Sequence alignment of the L protein revealed an additional long sequence (residues 623–710) in the RdRp domain of henipaviruses compared to the other viruses of *Mononegavirales* (**Fig. S4A**). However, this long region appears to be flexible and unresolved in both the L_1–1,451_-P and L-P complex maps. To further understand the potential structure of this long region within the polymerase, we performed AlphaFold2 (Jumper et al., 2021) modeling of the L protein. In the predicted NiV polymerase structure, this region is located on the periphery of the polymerase active site cavity, connecting the supporting helix (aa 587–601) to a β-strand (aa 712–722), both of which were resolved in our structures (**Fig. S4B**). A recent report has shown that this long region does not contribute to the folding of the RdRp domain but is crucial for the function of the polymerase (Hu et al., 2025).

Unlike the cap-snatching mechanism in orthomyxoviruses (Te Velthuis et al., 2021; Xue et al., 2024), the L protein in NNS RNA viruses utilizes its own PRNTase domain and MTase domain to complete the capping process. mRNA capping aids in evading host innate immunity, stabilizing newly synthesized mRNA, and further ensuring efficient viral protein translation. In the PRNTase domain of the NiV polymerase, two motifs are known to exist: the G^1273^-X^1274^-X^1275^-T^1276^ motif located on the priming loop (aa 1,254–1,291) and the H^1347^-R^1348^ motif located on the intrusion loop (aa 1,337–1,362). Sequence alignment shows these motifs are conserved among NNS RNA viruses (**Fig. S5A**). The GXXT motif is involved in binding of the capping guanosine nucleotide, whereas the HR motif serves as the site for covalent RNA attachment (Liang et al., 2015). Proper positioning of the priming and intrusion loops, ensuring an appropriate distance between the GXXT and HR motifs, is considered crucial for the capping reaction to occur (Gilman et al., 2019).

NNS RNA virus polymerases have been captured in only a few distinct functional states, limiting our understanding of their mechanisms of action. Most available NNS RNA virus polymerase structures were determined without RNA bound. The priming loops in RNA bound structures of EBOV and RSV polymerases are retracted from the polymerase active site cavity. Similarly, in previous apo HMPV (Pan et al., 2020), NDV (Cong et al., 2023), and PIV5 (Abdella et al., 2020) polymerases, their priming loops also retract from the polymerase active site cavity (**Fig. S5C and S5D**). Retracted priming loop structures have been proposed to create more space in the polymerase active site cavity to accommodate incoming nucleotides to be polymerized into the synthesizing RNA chain, linking retracted priming loop structures with RNA synthesis elongation. For the apo RABV and VSV polymerase complex structures (Jenni et al., 2020; Liang et al., 2015), their priming loops extend into the polymerase active site cavity, positioned directly opposite to the polymerase active site (**Fig. S5C and S5D**). The complete structures of the priming loop (aa 1,254–1,291) and intrusion loop (aa 1,337–1,362) are not fully resolved in our NiV structures. While the trajectory of the NiV intrusion loop is challenging to determine due to disorder, the backbone trajectories of both ends of the NiV priming loop suggest a conformation compatible with retraction from the polymerase active site cavity, similar to those observed in previous apo HMPV, NDV, and PIV5 polymerase complex structures. Therefore, the priming and intrusion loops show particularly high flexibility in our apo NiV polymerase structure, by comparison with previous NNS virus polymerase structures in apo state. The priming loop being in the polymerase active site cavity has been implicated in the *de-novo* initiation of RNA synthesis (Liang et al., 2015). Being in a retracted position in an apo polymerase, the NiV priming loop likely needs to undergo complex conformational change to facilitate RNA synthesis initiation.

Phylogenetically, polymerases of NNS RNA viruses and segmented negative sense (SNS) RNA viruses are relatively close (Mönttinen et al., 2021). Among SNS RNA viruses, orthomyxovirus polymerases are the best characterized. A number of orthomyxovirus polymerase structures captured in elongation states were determined (Kouba et al., 2019; Wandzik et al., 2020; Xue et al., 2024). These structures showing very similar arrangement between the bound elongating RNA and their RdRp domain. Due to the lack of NNS RNA virus polymerase structures in an elongation state with RNA bound, therefore, we superposed the orthomyxovirus Thogoto virus (THOV) polymerase structure (Xue et al., 2024) in an RNA elongation state, containing a template-nascent RNA duplex, to the NiV polymerase structure, for further insights into NiV polymerase RNA synthesis mechanism. The template-nascent RNA duplex captured within the THOV polymerase can be well accommodated by the NiV polymerase cavity (**Fig. 1D**). Four different solvent accessible channels within the NiV polymerase have been identified. Based on the trajectories of the template and nascent RNA strands accommodated within the NiV polymerase cavity, the four channels within the NiV polymerase have been assigned as the template RNA entry channel, template RNA exit channel, nascent RNA exit channel and NTP entry channel (**Fig. 1D**). Of note, the identified nascent RNA exit channel is primarily formed within the PRNTase domain and the opening of the nascent RNA exit channel can potentially deliver the nascent RNA into the AlphaFold modeled structures of the L-protein C-terminal domains, which contain the enzyme activities for mRNA cap methylation (**Fig. 1D**). Further analysis of polymerase cavity electrostatics reveals that the template RNA entry channel is less positively charged, by comparison with RSV (Cao et al., 2024) and EBOV (Peng et al., 2023) polymerases (**Fig. S6**).

### Essential PRNTase domain zinc-binding sites

**Figure 2.**
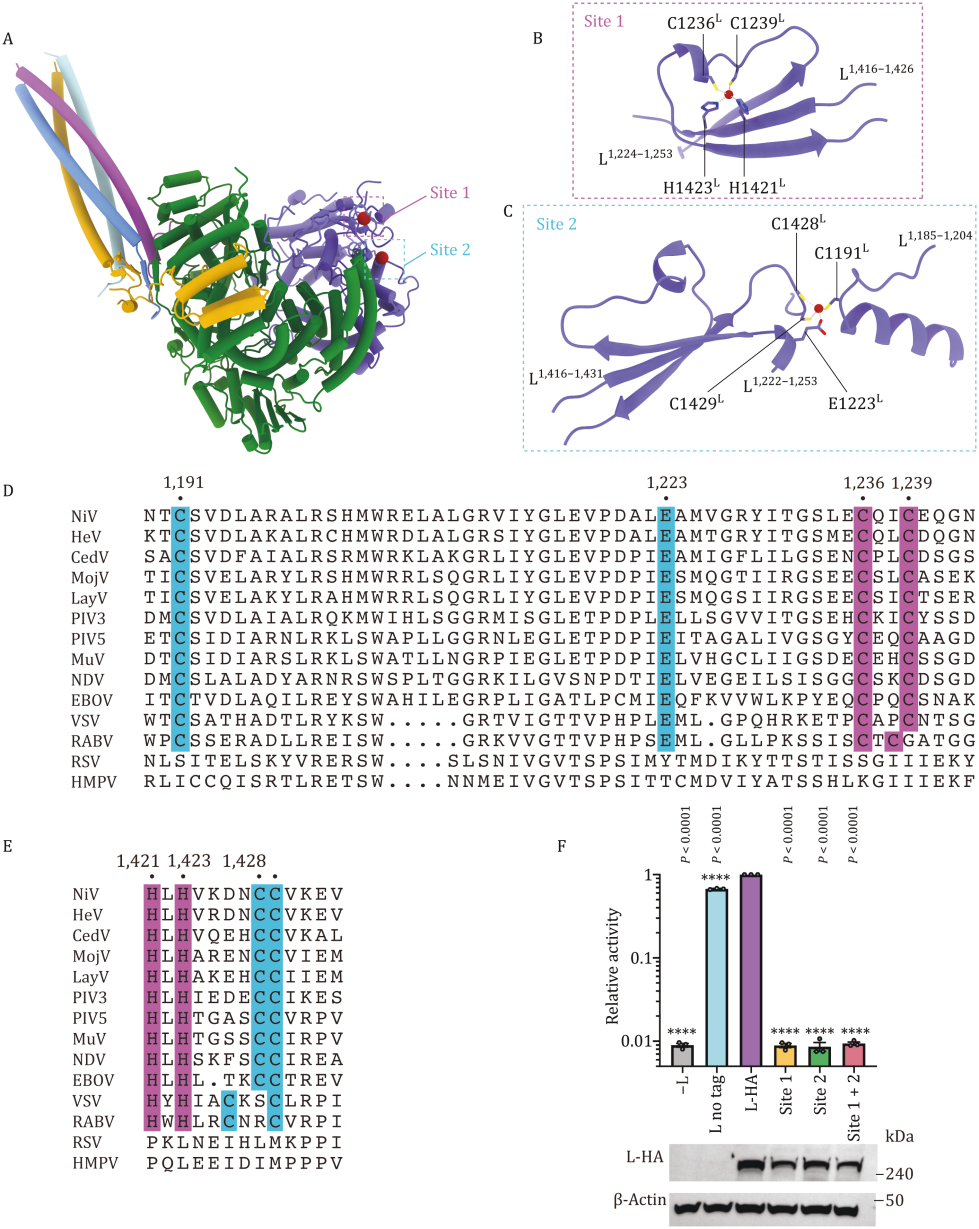
**Two zinc-binding sites within the PRNTase domain of NiV.** (A) Two zinc-binding sites, Site 1 and Site 2, are shown in the NiV L_1–1,451_-P complex structure. Their locations are marked by magenta and cyan dashed boxes. (B and C) Detailed views of the two zinc-binding sites within the PRNTase domain of NiV. Residues interacting with two zinc ions are shown and labeled, gray dashed lines indicate the metal coordinations. (D and E) Multiple sequence alignment of amino acid residues adjacent to the two zinc-binding sites of selected NNS (nonsegmented negative-sense) RNA viruses. The NiV site 1 residues and putative site 1 residues in other NNS viruses are shaded magenta. The NiV site 2 residues and putative site 2 residues in other NNS viruses are shaded cyan. L protein sequences analyzed are NiV (Nipah virus), HeV (Hendra virus), CedV (Cedar virus), MojV (Mojiang virus), LayV (Langya virus), PIV3 (Parainfluenza virus type 3), PIV5 (Parainfluenza virus 5), MuV (Mumps virus), NDV (Newcastle disease virus), EBOV (Ebola virus), VSV (Vesicular stomatitis virus), RABV (Rabies virus), RSV (Respiratory syncytial virus), and HMPV (Human metapneumovirus). Site 1 residues (C1236, C1239, H1421, H1423) and site 2 residues (C1191, E1223, C1428, C1429) are highly conserved among NNS RNA viruses (except for RSV and HMPV). (F) Effects of mutations in the two zinc-binding sites on NiV polymerase activity as measured by a Gaussia-luciferase mini-replicon assay. Polymerase activities are reported as mean ± SEM from three independent experiments (*n* = 3). All statistics used one-way ANOVA Dunnett’s multiple comparisons test (ns, *P* > 0.05; *, *P* < 0.05; **, *P* < 0.01; ***, *P* < 0.001; ****, *P* < 0.0001).

Two zinc-binding sites are located within the PRNTase domain of NiV L protein (**Fig. 2A**). The zinc ion in Site 1 is coordinated by the sidechains of C1236^L^, C1239^L^, H1421^L^, and H1423^L^ (**Fig. 2B**), while the zinc ion in Site 2 is coordinated by sidechains of C1191^L^, C1428^L^, C1429^L^, and E1223^L^ (**Fig. 2C**). To investigate whether these zinc-binding sites are functionally important, we performed alanine substitution mutations at Site 1 (C1236A^L^ + C1239A^L^) and Site 2 (C1428A^L^ + C1429A^L^) (**Fig. 2F**). The mutations reduced L protein expression to some extent, however, they completely abolished NiV polymerase activity in the mini-replicon assay (**Fig. 2F**). An L protein expression titration experiment shows that mini-replicon activity was still detected with even greater reduction in L protein expression (**Fig. S7**). These results indicate that these zinc-binding sites are essential for the function of the NiV polymerase. Sequence alignment shows that the two zinc-binding sites are highly conserved among NNS RNA virus polymerases, except for *Pneumoviridae*, including RSV, HMPV (**Fig. 2D and 2E**).

### Structure of the NiV P protein

Previously, crystal structures of a truncated version of NiV P protein show residues 476-576 forming a tetrameric helix bundle (Bruhn et al., 2014, 2019). In our L-P complex cryo-EM structures, the full-length P proteins also form a tetramer. The four monomers were designated P1–P4. P1 shows residues 525–579, 593–610, and 632–709; P2 shows residues 525–579; P3 shows residues 525–595; P4 shows residues 525–583 (**Fig. S8**). Therefore, cryo-EM structures resolved fewer residues on the P protein N-terminal side but more residues on the C-terminal side, by comparison with the crystal structures. The absence of P protein N-terminal region densities suggests their flexibility in the polymerase complex. P protein N-terminal region is known to bind nascent N protein (N^0^) (Curran et al., 1995; Mavrakis et al., 2006; Yabukarski et al., 2014), flexibility presumably allow the P N-terminal regions in the P protein bundle to perform spatial search to facilitate loading of multiple copies of N^0^ onto the nascent viral RNA. The P_OD_ regions forming the tetrameric coiled coil are well-resolved for all the four P monomers. A P_XD_ domain is resolved in the cryo-EM map for the P1 monomer (**Figs. 1** and **S8**). The tetrameric P protein bundle is formed and stabilized through hydrophobic interactions and hydrogen bonds among the P_OD_ regions of monomers, consistent with the crystal structures (Bruhn et al., 2014, 2019).

**Figure 3.**
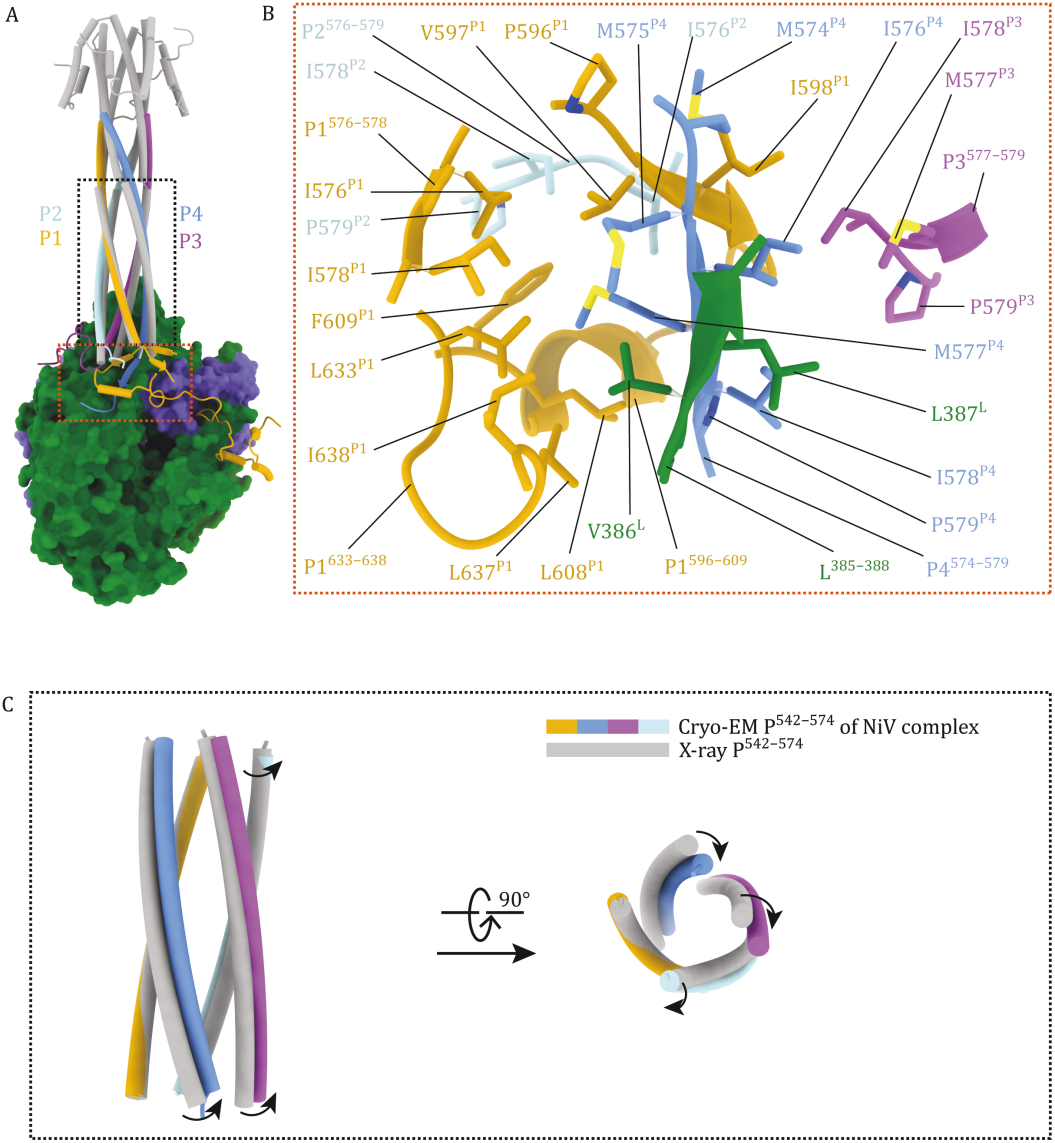
**Binding of the P protein bundle tetramer on the L protein.** (**A** and **B**) Binding of the P protein bundle tetramer on the NiV L protein. P1, P2, P3, and P4 monomers of the P protein bundle are colored yellow, cyan, magenta and blue, respectively. A crystal structure of tetrameric NiV P (gray, PDB: 4N5B) is superposed onto the P protein bundle of the cryo-EM structure of NiV L_1–1,451_-P complex. Orange and black dashed boxes indicate detailed views in panels (B) and (C). (B) A detailed view of a hydrophobic core formed by structural elements extended from the C-terminal ends of P1–4 P_OD_ helices and L^385–388^. (C) The superposition of cryo-EM (colored) and X-ray (gray) structures based on the P1 monomer identifies a rotational conformational change of the P protein bundle upon L protein binding.

**Figure 4.**
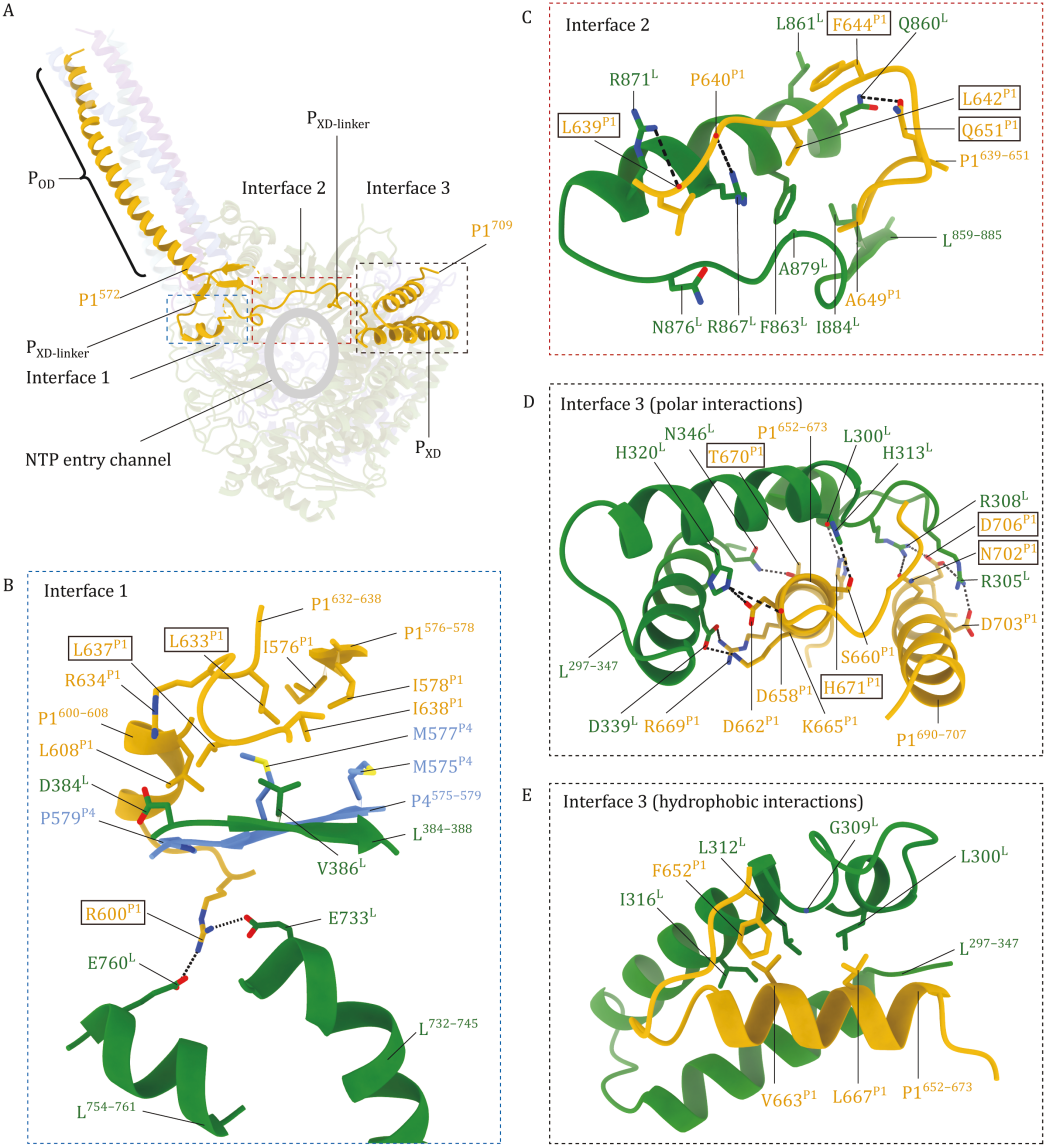
**The binding interfaces between NiV L and P1.** (A) The locations of the three interaction interfaces between NiV L and P1 are marked by the dashed boxes on the polymerase complex structure. The three interfaces are shown in detail in panels (B–E). (B–E) Close-up views of interactions in interface 1 (B), 2 (C), and 3 (D and E) between NiV L (green) and P1 (yellow). Residues involved in the three interfaces are shown and labeled, black dashed lines indicate hydrogen bonds or electrostatic interactions. Mutated P protein residues are marked with black solid-line boxes.

**Figure 5.**
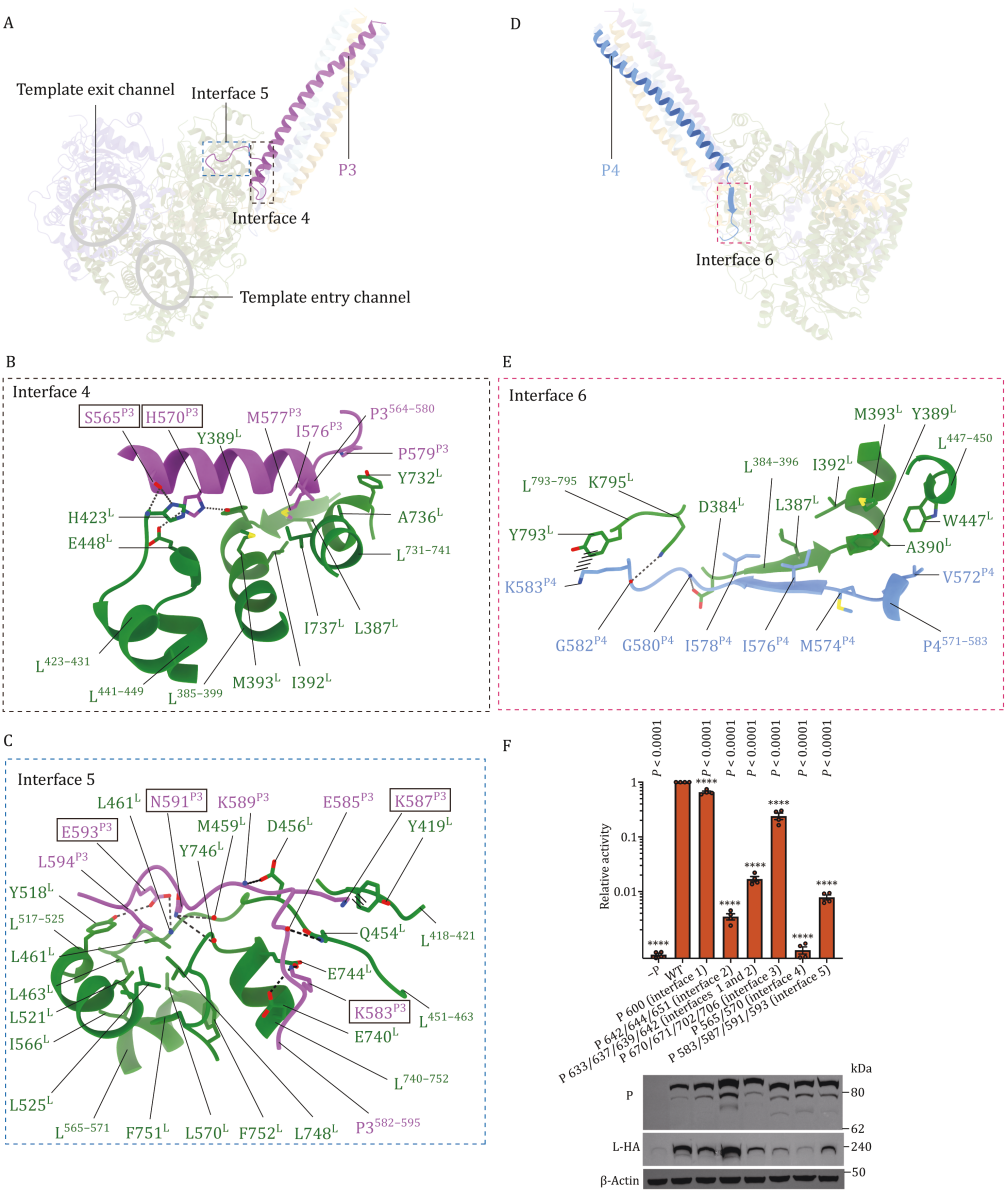
The binding interfaces between NiV L and P3 or P4 monomer of P protein bundle. (A) Overall structure of the L-P complex with dashed boxes showing the locations of the two interaction interfaces between NiV L and P3. Structural details in dashed boxes are shown in (B) and (C). (B and C) Sidechains of selected interacting residues in the interfaces 4 and 5 between NiV L (green) and P3 (magenta) are shown. Hydrogen bonds and salt bridges are shown as thin dashed lines; thick dashed lines indicate cation-π interactions. (D) Overall structure of the L-P complex with a dashed box to show the location of the interface between NiV L and P4. Structural details in the dashed box is shown in (E). (E), Sidechains of selected interacting residues in the interface 6 between NiV L (green) and P4 (blue) are shown. (F), Effects of mutations on selected L–P interacting residues on NiV polymerase activity as measured by a mini-replicon driven Gaussia-luciferase assay. Mutated P protein residues are marked with black solid-line boxes. Activities are reported as mean ± SEM from four independent experiments (*n* = 4). All statistics used one-way ANOVA Dunnett’s multiple comparisons test. (ns, *P* > 0.05; *, *P* < 0.05; **, *P* < 0.01; ***, *P* < 0.001; ****, *P* < 0.0001).

Of interest, being full-length proteins, P protein residues (S573^P^-I576^P^), at the C-terminal ends of the P protein helix, forming helical structures in the X-ray structures, are unfolded to various degree in our cryo-EM structures. The unfolded P_OD_ ends extend to form various helical, sheet and loop structures (**Fig. S8**). These structures interact to form a hydrophobic core at the base of the helix bundle (**Figs. 3A, 3B and****S9**). In particular, P4 M575^P4^-I578^P4^ refold into a β-strand in the cryo-EM structure (**Figs. 3B and****S10**) to interact with a β-strand formed by residues 385^L^-388^L^ of the L protein to assemble an antiparallel β-sheet (**Figs. 3B and****S10**). These structure elements engage further hydrophobic contacts and other interactions to stabilize the interaction between L and P proteins at the base of the P protein bundle (also see **Figs. 4 and 5**).

When the cryo-EM and X-ray P protein structures are aligned through the P1 monomer, P2, P3, and P4 display varying degrees of displacement, with P3 and P4 showing notable rotational shifts towards the L protein (**Fig. 3C**), likely due to their interactions with the L protein as captured in the cryo-EM structure (see below). These findings demonstrate the structural adaptability of the P protein, which facilitates its binding with the L protein. The detailed interactions between P protein monomers and L protein are described below (also see **Table S2**).

### NiV P1–L interaction is most extensive among the P bundle protomers

The P protein, serving as a cofactor, interacts with the L protein to participate in NiV RNA synthesis (Bloyet et al., 2016b; Morin et al., 2016). In the absence of the P protein, the L protein is unstable (Bloyet et al., 2019; Canter and Perrault, 1996) and cannot be recruited to the template for viral RNA synthesis showing no mini-replicon activity (**Fig. 5F**). Our structures show that extensive interactions occur between the NiV L protein and 3 out of the 4 P proteins in the tetrameric P protein bundle (**Figs. 1C,****S11****and****S12**). No interaction was found between L and P2, which is distal from the L protein within the P protein bundle (**Fig. 1C**). A large combined interface area of 3,537 Å^2^ was calculated between the L and P proteins, with contributions of 1,743, 1,176, and 617 Å^2^ from P1, P3, and P4, respectively. Extensive electrostatic, hydrogen bonding, and hydrophobic interactions are found in the L–P interface.

Residues 572–709 of P1, form the P1_XD_ domain and the linker between the P1_XD_ and P1_OD_. The P1_XD_ domain and P1_XD_ linker adopt a “W” topology and are positioned above the NTP entry channel of the L protein (**Fig. 4A**). It has been proposed for HMPV (Pan et al., 2020) that basic residues (K224^P1^, K227^P1^, K229^P1^, R241^P1^, K243^P1^, K250^P1^, K254^P1^, and K256^P1^) in the C-terminal region of the P1 subunit, located around the HMPV polymerase NTP entry channel, form a positively charged arch that may attract NTPs to the NTP entry channel (**Fig. S13**). Similarly, for NiV, we observe several basic amino acids (K595^P1^, R600^P1^, R634^P1^, R661^P1^, K665^P1^, R669^P1^, R675^P1^, and K687^P1^) present in the P1_XD_ domain and P1_XD_ linker near the NTP entry channel (**Figs. 4A and****S13**). The large interface area between P1 and the L protein can be divided into three regions: Interface 1 is located at the base of the P protein bundle, Interface 2 is situated above the NTP entry channel, and Interface 3 involves the P1_XD_ domain (**Fig. 4A**).

In interface 1 (**Fig. 4B**), structural elements, P1^576^^–^^578^, P1^600^^–^^608^, and P1^632–638^ are part of the hydrophobic core formed at the base of the P protein bundle (see **Fig. 3B**). Isoleucine and leucine residues from these structural elements form a hydrophobic cluster to contact V386^L^ of L^384^^–^^388^. This hydrophobic interaction is further enhanced by hydrophobic contacts from P4^575^^–^^579^ involving P579^P4^, M575^P4^, and M577^P4^. The above hydrophobic L–P interaction is further stabilized by a pair of prominent salt bridges: R600^P1^-E733^L^ and R600^P1^-E760^L^. A potential long-range electrostatic interaction between D384^L^ and R634^P1^ can be also located at the interface between L and P1.

In interface 2 (**Fig. 4C**), the P1^639–651^ loop, as part of the P_XD_ linker, is stabilized above the NTP entry channel by binding with L^859–885^ mainly through three hydrogen bonds (P640^P1^-R867^L^, L639^P1^-R871^L^, Q651^P1^-Q860^L^) and hydrophobic interactions (involving L642^P1^, F644^P1^, L861^L^, F863^L^, A879^L^, and I884^L^).

In interface 3 (**Fig. 4D and 4E**), P1^652–673^ and P1^690–707^ helices are part of the P1_XD_ domain. Their polar residues (S660^P1^, D662^P1^, R669^P1^, K665^P1^, T670^P1^, H671^P1^, N702^P1^, D706^P1^, and D703^P1^) are located in close proximity to interact with the residues (L300^L^, R305^L^, R308^L^, H313^L^, H320^L^, D339^L^, and N346^L^) on L^297–347^ of the RdRp domain. Additionally, the P1^652^^–^^673^ helix, as part of the P1_XD_ domain, forms a tight hydrophobic core with L^297^^–^^347^, involving hydrophobic residues I316^L^, L312^L^, G309^L^, L300^L^ of L^297^^–^^347^, and F652^P1^, V663^P1^, L667^P1^ of P1^652^^–^^673^. Interactions identified within interface 3 appear to allow stable binding of the P1_XD_ domain on the L protein RdRp surface, making P1_XD_ the only XD domain resolved among the four P monomers.

### P3 and P4 interactions further anchor the P tetramer on NiV L

The interface area between P3 and L is 1,176 Å^2^, ranking 2nd among the L-interacting P proteins. The P3–L interface area can be divided into two distinct regions (**Fig. 5A**), namely, interface 4, located at the base of the P protein bundle, and interface 5, involving the P3_XD_ linker region. These two interfaces are formed by interactions from the P3^564–580^ helix, located at the C-terminal end of the P3_OD_ helix (interface 4, **Fig. 5B**), and the P3^582–595^ loop, as part of the P3_XD_ linker region, (interface 5, **Fig. 5C**), with the L protein.

In interface 4, we observed that in helix P3^564–580^, the side chain of S565^P3^ forms a bifurcated hydrogen-bond network with H423 of the L protein. Meanwhile, H570^P3^ interacts with E448^L^ and Y389^L^ of the L protein through hydrogen bonds. Additionally, I576^P3^ and M577^P3^, which are part of the hydrophobic core formed at the base of the P protein bundle (**Fig. 3B**), insert into a large hydrophobic pocket formed by the side chains of L387^L^, I392^L^, M393^L^, Y732^L^, A736^L^, and I737^L^ in the L protein, further stabilizing the binding of P3^564–580^ to the L protein (**Fig. 5B**). In interface 5 (**Fig. 5C**), the P3_XD_ linker loop (P3^582^^–^^595^) extends from the P3^564^^–^^580^ helix in interface 4 (see **Fig. 5B**), at the C-terminal end of the P3_OD_ helix, to further interact with the L protein surface (**Fig. 5C**). By comparison with the available polymerase complex structures of *Mononegavirales*, the interactions engaged by the P3_XD_ linker seem to be distinct (see below). Specifically, K583^P3^ forms salt bridges with E740^L^ and E744^L^, while another basic residue, K587^P3^, engages in a cation-π interaction with Y419^L^. N591^P3^ forms hydrogen bonds with the main-chain carbonyls of M459^L^ and Y746^L^. The negatively charged carboxyl group of E593^P3^ accepts hydrogen bonds from the main-chain amide of L461^L^ and the hydroxyl group of Y518^L^ sidechain. Notably, towards the C-terminus of the P3^582^^–^^595^ loop, L594^P3^ inserts into a hydrophobic core on the L protein, which is formed by multiple leucine and phenylalanine residues (**Fig. 5C**). The above interactions appear to firmly anchor the P3_XD_ linker on the L protein surface.

In the interface between P4 and L (interface 6, **Fig. 5D and 5E**), residues of P4^571^^–^^583^, extending from the end of the P4_OD_ helix, are adjacent to the β-strand of L^384^^–^^396^, forming a continuous β-sheet. This interface facilitates extensive side chain interactions, such as hydrogen bonds (G580^P4^-D384^L^ and G582^P4^-K795^L^), cation-π interactions (K583^P4^-Y793^L^) and hydrophobic contacts (V572^P4^, M574^P4^, I576^P4^, and I578^P4^ to L387^L^, Y389^L^, A390^L^, I392^L^, M393^L^, and W447^L^). Of note, M574^P4^, I576^P4^, and I578^P4^ are also part of the hydrophobic core formed at the base of the P protein bundle (**Fig. 3B**). These interactions appear to anchor P4^571–583^, as part of the P4_XD_ linker N-terminal region, firmly on the L protein surface.

### P protein mutations within the L–P interface affect mini-replicon activity

**Figure 6.**
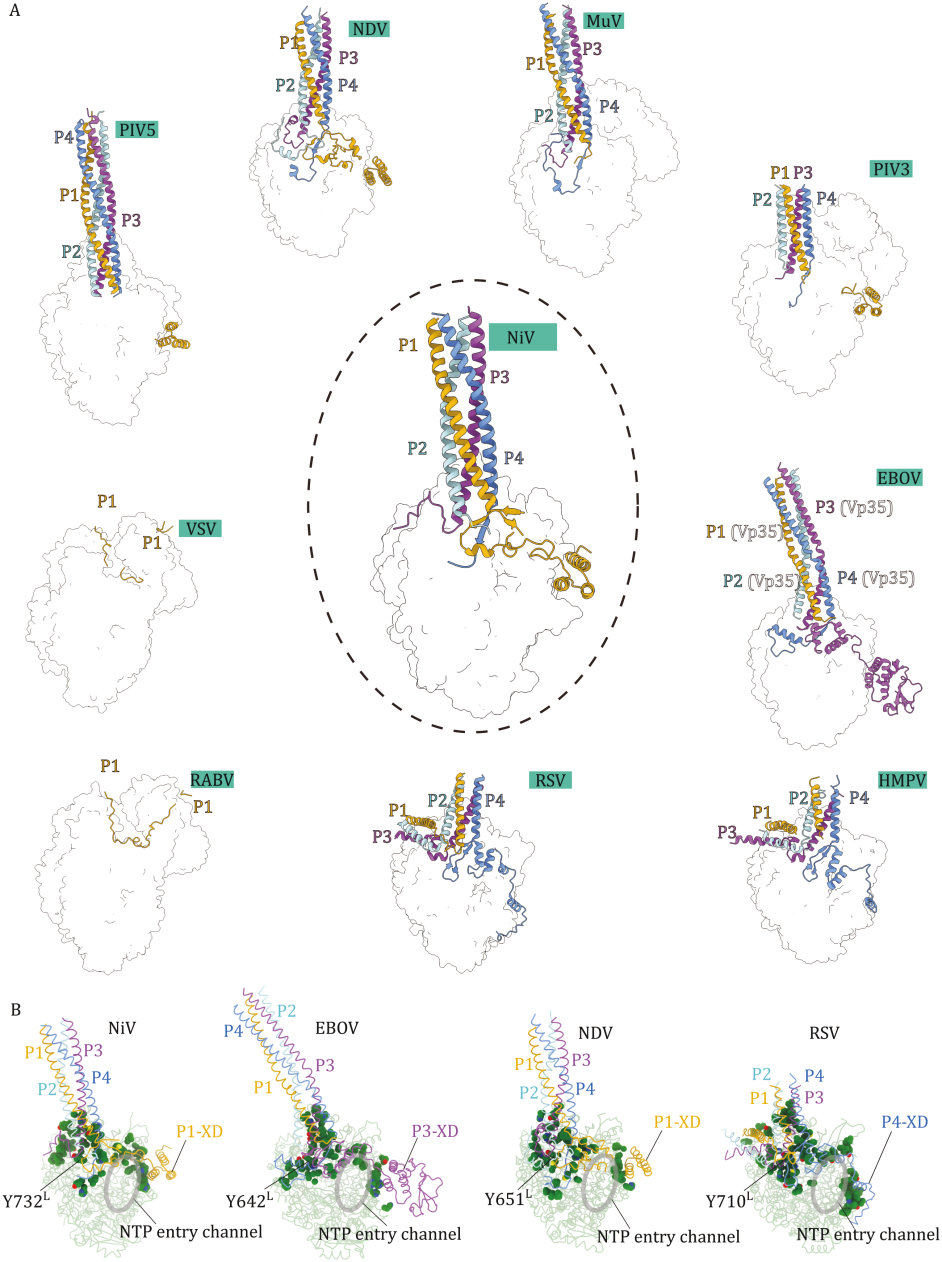
**Binding of phosphoproteins compared among NNS RNA virus polymerase complexes.** (A) Polymerase complexes of NiV, PIV5 (PDB: 6V85), NDV (PDB: 7YOV), MuV (PDB: 8IZL), PIV3 (PDB: 8KDC), EBOV (PDB: 7YER), HMPV (PDB: 6U5O), RSV (PDB: 6UEN), RABV (PDB: 6UEB), and VSV (PDB: 6U1X) are aligned based on their L proteins. The L protein parts of the complexes are shown as transparent surfaces and the phosphoproteins are shown as cartoon representations to highlight their assembly status. Monomers within bundle forming phosphoproteins are assigned as P1–P4 according to the arrangement in the NiV P protein bundle and colored accordingly (P1, yellow; P2, cyan; P3, magenta; P4, blue). (B) Comparison of the phosphoprotein interacting hydrophobic and aromatic residues on the surfaces of NiV, NDV, EBOV, and RSV L proteins. Sidechains of interacting hydrophobic and aromatic residues are shown as spheres.

Among available NNS RNA virus polymerase complex structures, paramyxovirus, pneumovirus and filovirus P proteins adopt tetrameric helix bundle structures and form complex with L proteins (**Fig. 6**). In these structures, their P protein C-terminal domains (CTD, defined as equivalent to P_XD_ linker and P_XD_ domain combined in paramyxoviruses) are in nonequivalent positions. The CTD of one monomer in the P tetramer, interacts stably with the L protein’s RdRp core, while the other three CTDs appears to be flexible (**Fig. 6**). The NiV P protein CTD P_XD_ domain has been shown to interact with the NiV nucleocapsid tail (Bourhis et al., 2022). Among several NNS viruses, P protein CTD-nucleocapsid interactions have been shown essential for keeping the polymerase complex attached to the template during RNA synthesis (Bloyet et al., 2016a; Brunel et al., 2014; Cox et al., 2017). Based on the above results, a proposed RNA synthesis mechanism suggests that the three flexible P protein CTDs sequentially interact with the RNP complex, guiding the template toward the L protein template entry channel (Abdella et al., 2020; Pan et al., 2020).

A structural superposition of the paramyxovirus polymerase complexes reveals that while L can be well aligned, P proteins from different species exhibit conformational deviations on the surface of the L proteins (**Figs. 6 and****S14**). Within the *Paramyxovirus* family, the NiV P shares low sequence identity with PIV5 (21.33 %), PIV3 (21.15 %), NDV (22.01 %), MuV (21.20 %). Paramyxovirus PIV3, PIV5, and MuV and NDV L-P complexes show similar structural features, with the P from NDV being the best resolved (**Fig. 6A**). A comparison reveals that the NiV P1 in interfaces 1 and 2 adopts a distinct structure compared with the equivalent regions in NDV (compare **Fig. 4 to****Fig. S15**), such that P1 extends and forms stable interactions above the NTP entry channel.

There are also notable differences between NiV and NDV in the L-P interface 5 (compare **Fig. 5 to****Fig. S16**). Notably, in interface 5, the loop P3^582–595^ within the P3_XD_ linker region extends along the surface of NiV L to interact with a hydrophobic surface. In contrast, the corresponding region in NDV extends away from this hydrophobic patch. The different trajectories of the P_XD_ linkers on L surfaces result in the P3 CTD of our NiV L-P complex structure being positioned particularly close to the template entry channel. The proximal location of the P3^582–595^ in NiV L–P complex above the entry and exit channels of the template RNA (**Fig. 5A and 5C**), suggesting that the C-terminal XD domain extending from P3^582–595^ may be particularly important to facilitate the entry of template RNA into the polymerase.

To investigate the impact of L–P interactions on NiV mini-replicon activity (**Fig. 5F**), we first performed a P protein expression titration experiment and examined its effect on L protein expression (**Fig. S17**). The experiment shows that a reduction in P protein expression greatly lowers L protein expression and mini-replicon activity, confirming previous result showing that L protein expression and stability are highly dependent on P expression.

We further introduced alanine substitutions into specific clusters of amino acid residues at various L–P interaction interfaces. In interface 1, an R600A^P^ substitution was made at the base of the P protein bundle (**Fig. 4B**). For interface 2, L642A^P^/F644A^P^/Q651A^P^ in the P_XD_ linker C-terminal region above the NTP entry channel were introduced (**Fig. 4C**). L633A^P^/L637A^P^/L639A^P^/L642A^P^ substitutions spanning both interfaces 1 and 2 were introduced (**Fig. 4B and 4C**). In interface 3, T670A^P^/H671A^P^/N702A^P^/D706A^P^ substitutions targeting the L-interacting P_XD_ surface were introduced (**Fig. 4D**). For interface 4, S565A^P^/H570A^P^ substitutions were made in the C-terminal region of the P protein helix bundle (**Fig. 5B**). Finally, in interface 5, K583A^P^/K587A^P^/N591A^P^/E593A^P^ substitutions were introduced in the P_XD_ linker N-terminal region (**Fig. 5C**).

Compared to the WT P control, all P mutants showed reduction in mini-replicon activity, indicating that L–P interface residues are important for the activities of the NiV polymerase. By western blot, all P mutants showed maintained or increased P protein expression levels (**Fig. 5F**). All mutants, except for the interface 2 L642A^P^/F644A^P^/Q651A^P^ mutant, showed decreased L protein expression levels and mini-replicon activities. The reduction in L protein expression was particularly pronounced for the interface 3 and 4 mutants. Based on the dependency of L expression on P expression, as shown by the P protein expression titration experiment (**Fig. S17**), a reduction in L expression without a corresponding reduction in P expression most likely indicates a disruption of L–P interaction, which affects the stability of L protein (Bloyet et al., 2019; Canter and Perrault, 1996). The L642A^P^/F644A^P^/Q651A^P^ mutant exhibited increased L protein levels despite a reduction in mini-replicon activity. The maintained L protein expression level supported by the L642A^P^/F644A^P^/Q651A^P^ mutant suggests that these mutations may directly affect polymerase mini-replicon activity.

### Highly varied L–P interactions among *Mononegavirales*

We further compare the NiV L–P complex structure to the other available polymerase complex structures of *Mononegavirales* (**Fig. 6A**). Among these, the P proteins of *Rhabdoviridae* (VSV and RABV) do not adopt helix-bundle structures with interaction pattern entirely distinct from other *Mononegavirales*. The P proteins of VSV (Jenni et al., 2020; Liang et al., 2015) and RABV (Horwitz et al., 2020) associate to form a dimer to bind their cognate L through interactions from the N-terminal region of a P monomer. Although *Rhabdoviridae* P proteins lack a XD domain, their C-terminal domain (CTD) has been shown to associate with nucleocapsid to allow L recruitment (Whitehead et al., 2023). Helix bundle structures have been observed for P proteins bound to polymerases of *Paramyxoviridae* (NDV and MuV, PIV3, and PIV5)*, Pneumoviridae* (RSV and HMPV)*,* and *Filoviridae* (EBOV, the P protein equivalent is named Vp35). In the above structures, the bases of the P protein bundles are all anchored to similar areas on the L proteins in the RdRp “fingers” (**Fig. 6A**). However, different P protein assembly and interaction patterns are observed among the above virus families. For paramyxoviruses, P proteins appear to assemble similarly, such that P3 and P4 are positioned most proximal to the polymerase. One of the four C-terminal P_XD_ domains available from the P protein bundle is usually found stably bound to the polymerase except for the MuV polymerase complex, suggesting that the P_XD_–L interaction may be dynamic among different viruses. In the structures showing stable P_XD_–L interactions (PIV5, NDV, PIV3), the P_XD_ domains are discontinuous from the P protein bundles, and the P_XD_ domains are deduced to originate from P1 of the P protein bundle based on the P protein bundle assembly geometries (**Fig. 6A**). In the NiV polymerase complex structure, the linker to the P1_XD_ domain is mostly resolved allowing us to confirm that the stably bound P1_XD_ domain extends from P1 within the P protein bundle. The P1_XD_ linker region is resolved due to its stable interaction above the NTP entry channel. In contrast, a stable interaction is not found for the P_XD_ linkers above the NTP entry channels, in the other non-*henipavirus* paramyxovirus polymerase complexes. The P protein interaction patterns also vary markedly for EBOV, RSV and HMPV. In EBOV, instead of P1, the P_XD_ domain from P3 of the Vp35 bundle interacts stably with the polymerase. Notably, in EBOV, the P3_XD_ linker is resolved and stably bound above the NTP entry channel in a similar fashion as observed for the NiV P1_XD_ linker. In RSV and HMPV, different from paramyxoviruses and filoviruses, XDs from P4 of the P protein bundles extend and form stable interactions with the polymerases. In both structures the linker regions to the XDs are resolved and positioned above the NTP entry channels (**Fig. 6A**).

Sequence alignment of L reveals that residues involved in hydrophobic interactions with P in NiV are somewhat conserved among paramyxoviruses (**Figs. S11****and****S18**). In contrast, the residues involved in hydrogen bond and salt bridge interactions with P in NiV are non-conserved (**Fig. S18**). To gain further insight, we compare the L-P interfaces of NiV, NDV, EBOV and RSV polymerase complexes, which are best resolved among *Mononegavirales*, focusing on interface hydrophobic and aromatic residues (**Figs. 6B and****S11**). The analysis reveals that hydrophobic and aromatic residues within the L–P interfaces show limited conservation among *Mononegavirales* (**Fig. 6B**). However, several common signatures can be identified: (i) A large hydrophobic patch can be identified on the RdRp “fingers” domain to interact with the base of the P protein bundle. (ii) A hydrophobic patch is located away from the P protein bundle binding site, across the NTP entry channel, to bind the P_XD_ domains. The P_XD_ interacting hydrophobic patches are similar in NiV, NDV and EBOV. In contrast, the P_XD_ interacting patch is located lower on the RSV L protein surface, likely due to RSV P_XD_ domain adopting a different structure (**Fig. 6B**). A highly conserved tyrosine (Y732 in NiV, Y651 in NDV, Y642 in EBOV, Y710 in RSV) is identified within the hydrophobic patches interacting with the bases of the P protein bundles (**Fig. 6B**). This residue interacts with P3 of the P protein bundles in NiV and NDV, while it interacts with P3 and P4 in EBOV and RSV. This tyrosine appears to be highly conserved among L proteins known to interact with bundled P proteins (Figs. S11**and**S18). In all these polymerase complex structures, from the conserved tyrosine, hydrophobic residues extend upwards to interact with the lower parts of the P protein bundles in their helix regions. In NDV, RSV, and EBOV, hydrophobic residues around the conserved tyrosine are found to interact with the P_XD_ linker regions of P protein monomers with unbound P_XD_ domains (Fig. 6B). In contrast, less hydrophobic residues are found around the conserved tyrosine on the NiV L-protein to interact with the P_XD_ linker regions, as a result less P_XD_ regions are resolved for P2, P3, and P4 (**Fig. 6B**). Hydrophobic interactions with P_XD_ linkers likely affect P_XD_ dynamics in polymerase systems of *Mononegavirales*. Hydrophobic interactions are found above the NTP entry channel for NiV, RSV, and EBOV, connecting the P protein bundle interacting patches with the P_XD_ interacting patches (**Fig. 6B**). These interactions are consistent with that P_XD_ linkers are stably bound above the NTP entry channel in these polymerase complexes. Amino acid differences in the P_XD_ linker region between NiV and NDV (**Figs. S12** and **S19**), might result in the P_XD_ linker region in P1 to extends away from the polymerase, adopting an unbound flexible conformation in NDV.

## Discussion

NiV continues to cause outbreaks with increased human–bat interaction. Small molecule drugs targeting polymerase activities have been developed for various viral diseases as successful therapeutics (Palazzotti et al., 2024). To potentially facilitate structure-based drug discovery, we have determined two NiV polymerase structures in complex with its phosphoprotein cofactor. The two structures differ in the L protein being either in a full-length or a truncated form. These two structures confirm that in the apo state, we captured the polymerase complex, the L-protein C-terminal domains—CD, MTase, and CTD are disordered. Previously, CD, MTase and CTD can usually be resolved for paramyxovirus L proteins, including MuV (Li et al., 2024), PIV3 (Xie et al., 2024), PIV5 (Abdella et al., 2020), and NDV (Cong et al., 2023). Therefore, our structures of the NiV polymerase complex reveal differences in the intrinsic flexibility of the C-terminal domains of the NiV L protein compared to other paramyxovirus L proteins. A recent report shows that the NiV L protein CTD becomes ordered upon RNA elongation (Sala et al., 2025). We confirm that the two zinc binding sites in the NiV PRNTase domain, which are conserved among available non-segmented negative-strand (NNS) RNA virus polymerase structures except for those of RSV and HMPV, are essential for its polymerase activity. Previously, various zinc-chelating compounds and metallocompounds with antiviral activities have been developed as potential treatments for retroviruses and herpesviruses, specifically targeting zinc fingers (Abbehausen, 2019; Asquith et al., 2019; Mjos and Orvig, 2014; Pannecouque et al., 2010; Rice et al., 1993). Some of these molecules have entered Phase I/II trials although none has been clinically approved (Abbehausen, 2019; Goebel et al., 2001). Despite likely challenges in pursing such therapeutic strategy, with the development of newer zinc finger inhibitors, treatment of NiV infections with zinc finger inhibitors may be further investigated (Abbehausen, 2019; Pannecouque et al., 2010). L–P interaction has been shown essential for polymerase activity (Bloyet et al., 2019; Sourimant et al., 2015). Comparison of the NiV polymerase phosphoprotein complex structure to other polymerase complex structures of *Mononegavirales* identifies a highly conserved tyrosine, among viruses with bundle-forming phosphoproteins (i.e., this tyrosine is not conserved in VSV, RABV and others known to have phosphoprotein cofactors that cannot form helix bundles). This tyrosine appears to serve as an anchoring point for the phosphoprotein bundle. Around this tyrosine, some conservation in polymerase surface hydrophobic residues engaged in L–P interaction can be observed among paramyxoviruses. Although hydrophobic residues on L around the conserved tyrosine are observed for L–P interaction in other NNS RNA virus polymerases, their sequences and locations are non-conserved. The semi-conservation of the P protein interacting hydrophobic patch among paramyxoviruses suggest this patch may serve as a druggable surface for the development of wider spectrum molecules against paramyxoviruses. Our NiV polymerase complex structures also reveal distinct arrangement in NiV P_XD_ domains. In NiV, P1_XD_ domain forms stable interactions with the polymerase away from the phosphoprotein bundle across the NTP entry channel, similar to other paramyxoviruses. Distinctively, the NiV P1_XD_ linker forms stable interactions above the NTP entry channel, similar to those seen in EBOV, RSV, and HMPV structures. In addition, via extensive interactions, the P2, P3, and P4 XD linker regions are anchored on the polymerase surface in a different manner from other available NNS RNA virus polymerase structures, implicating a difference in P_XD_ domain positioning. P_XD_ domain has been proposed to interact with the template bound N protein, difference in P_XD_ domain positioning may discriminate NiV polymerase activity with the other paramyxovirus polymerases. In summary, our cryo-EM structures of the NiV L–P complexes provide insights into the virus’s RNA synthesis and inform antiviral development.

Note added during revision: During the revision process of this article, several studies report the apo structure of the NiV L–P complex (Balıkçı et al., 2025; Hu et al., 2025; Peng et al., 2024; Wang et al., 2024; Yang et al., 2024). A comparison shows varied modeling of the P^632–656^ region above the NTP entry channel among our reported structures and released structures (Peng et al., 2024; Wang et al., 2024; Yang et al., 2024) (**Fig. S20**).

## Methods

### Cells

BSR-T7/5 cells (provided by coauthor Prof. Liqiang Feng’s group) were maintained at 37°C and 5% CO_2_ in Dulbecco’s modified Eagle’s medium supplemented with 10% fetal bovine serum, 1% penicillin/streptomycin (Gibco), and 1 mg/mL of G418 (MedChemExpress, HY-17561). *Spodoptera frugiperda* (Sf9) cells, maintained in SF-900 II SFM (Gibco) were used for generating recombinant baculovirus (rBV) stocks and protein expression. All cell lines used in this study are routinely checked for Mycoplasma and other microbial contaminations.

### Protein expression and purification

The genes for NiV L (GenBank: AAK29089.1) and P (GenBank: AAF73378.1) were condon-optimized for expression in insect cells. A PreScission protease cleavable 2× Strep and 1× Flag tag were added N-terminal to the L protein. Full-length (aa 1–2,244) or truncated L (aa 1–1,451) and full-length P (aa 1–709) genes were cloned into the pFast-DUAL vector for co-expression under the control of polH (for NiV L or L_1–1,451_) and p10 (for NiV P) promoters. Recombinant bacmid was generated using the Bac-to-Bac expression system. Recombinant baculovirus generation, amplification, and protein expression were carried out in Sf9 insect cells.

Cell pellet was lysed by sonication in lysis buffer A (50 mmol/L Tris-HCl, pH 8.0, 500 mmol/L NaCl, 10% glycerol, 2 mmol/L TCEP) supplemented with protease inhibitors (Roche, cOmplete, EDTA-free, 4693132001). After centrifugation (39,190 ×*g*, 60 min, 4°C), the supernatant was incubated with 3 mL Strep-Tactin resin (Cytiva, 29401324) for 2 h at 4°C. The beads were washed twice with buffer A before the target protein was eluted with buffer A containing 2.5 mmol/L d-desthiobiotin (Sigma-Aldrich, D1411). Subsequently, eluted fractions containing NiV L–P or L_1–1,451_-P complex were incubated with anti-FLAG beads (GenScript, L00432) for 3 h at 4°C before elution in buffer B (50 mmol/L Tris-HCl, pH 8.0, 500 mmol/L NaCl, 5% glycerol) containing 200 μg/mL FLAG peptide (GenScript, RP10586CN). The NiV L–P or L_1–1,451_-P complex was subsequently loaded onto a Superose 6 increase 10/300 GL (GE HealthCare, 29091596) pre-equilibrated with buffer B. The peak fractions were collected and stored at -80°C for further use. We routinely achieved ~3 times the purification yield for the truncated L_1–1,451_-P complex by comparison with the full-length L–P complex.

### NiV minigenome assay

NiV mini-genome was generated based on constructs previously reported (Bruhn et al., 2019; Halpin et al., 2004; Jordan et al., 2018), with slight modifications to adapt to the Gaussia luciferase (GLuc) reporter gene designed to express as a secreted protein. To create the NiV mini-genome, the NiV trailer—L 3′ UTR (untranslated regions)–GLuc—NP 5′ UTR (untranslated regions) leader—HDV (self-cleaving hepatitis delta virus ribozyme) sequence was synthesized and cloned into the pT7 vector (Yang et al., 2019).

For functional studies with the mini-genome system, BSRT7/5 cells at about 80%–90% confluency in 48-well plates were transfected with the NiV mini-genome plasmid (2 μg) and the helper plasmids encoding NiV L (1 μg) (GenBank: AAK29089.1), P (0.5 μg) (GenBank: AAF73378.1), and NP (1 μg) (GenBank: AAF73377.1) proteins. After 48 h post-transfection, the cell culture supernatant was harvested. After preloading 100 μL of Renilla Luciferase Assay Reagent (Promega, E2820) into a 96-well opaque, white plate, 20 μL of cell culture supernatant was carefully added and mixed well. The detection plate was then placed in a GloMax^TM^ 96 Microplate Luminometer (Promega) for reading.

Mutations C1236A^L^/C1239A^L^, C1428A^L^/C1429A^L^, and C1236A^L^/C1239A^L^/C1428A^L^/C1429A^L^ were inserted into the HA-NiV_L construct and assayed to test the function of the zinc binding sites in the NiV polymerase. To test the effect of P–L interface mutations, HA-NiV_L constructs containing the following muations: R600A^P^, L642A^P^/F644A^P^/Q651A^P^, L633A^P^/L637A^P^/L639A^P^/L642A^P^, T670A^P^/H671A^P^/N702A^P^/D706A^P^, S565A^P^/H570A^P^, and K583A^P^/K587A^P^/N591A^P^/E593A^P^ were tested. Gaussia luciferase activity was measured after 48 h transfection. Negative control experiments were performed by substituting the NiV L or P expression plasmid with an empty plasmid. L protein titration experiment was performed by transfecting a decreasing amount (0.125–1 μg) of helper plasmid encoding NiV L protein. P protein titration experiment was performed by transfecting a decreasing amount (0.0625–0.5 μg) of helper plasmid encoding NiV P protein.

### Western blot

To evaluate the impact of zinc binding site mutations and NiV L–P interface mutations on polymerase expression levels, BSR-T7/5 cells expressing mutant polymerases were collected 48 h post-transfection. The cells were lysed for 10 min at 98°C with 5× protein loading buffer (Solarbio, P1040). The samples were subjected to SDS-PAGE and transferred onto a PVDF membrane. After blocking with 5% (*w*/*v*) skimmed milk, the membrane was incubated with a mouse anti-HA monoclonal antibody (1:2,000 dilution, Sino Biological, 100028-MM10) or a custom rabbit polyclonal serum against a short synthetic peptide of NiV P (aa 414–427) (Bruhn et al., 2019) (1:2,500 dilution, GenScript) before horseradish peroxidase-conjugated goat anti-mouse or rabbit secondary antibody was incubated. The bands were detected using the SuperSignal™ West Pico PLUS Chemiluminescent Substrate kit (Thermo Scientific, 34580). β-Actin was used as an internal control and anti-β-actin antibodies (1:2,500 dilution, Sino Biological, 100166-MM10) were utilized.

### Cryo-EM sample preparation and data collection

To prepare NiV polymerase samples (L_1–1,451_-P/L-P), proteins were diluted to a concentration of 0.85 mg/mL in cryo-EM buffer (50 mmol/L Tris-HCl, pH 8.0, 500 mmol/L NaCl, 2 mmol/L TCEP, 5% (*v*/*v*) glycerol). For each sample, 3 µL of the sample was applied onto each glow-discharged (at 15 mA for 30 s in air, GloCube, Quorum) holey grid (ANTcryo^TM^ R1.2/1.3, Au 300 mesh grids). The grids were blotted for 2.5 s with a force of 4 at ~100% humidity and plunged into liquid ethane using an Vitrobot (Thermo Fisher). Cryo-EM grids were loaded onto a 300 keV Titan Krios electron microscope (Thermo Fisher) equipped with a Falcon4 direct electron detector with SelectrisX energy filter (slit width 10 eV) for data collection using EPU. Movies were collected and recorded in counting mode at a nominal magnification of 165,000 ×*g* with a calibrated pixel size of 0.73 Å and a defocus range from −0.6 to −2.4 μm. Gain-normalized movies of 30 frames were collected with a total exposure of ~50 e^–^/Å^2^.

### Cryo-EM image processing

The flow charts of cryo-EM data processing are shown in **Figs. S2****and****S3**. Sample-specific data collection and processing parameters are summarized in **Table S1**.

For the NiV L_1–1,451_-P sample dataset (**Fig. S2**), movie motion correction was performed using the RELION v4.0 (Kimanius et al., 2021) implemented MotionCor2 algorithm. Subsequent processing steps were performed in cryoSPARC v4.3 (Punjani et al., 2017). Micrographs were subjected to CTF estimation and manually inspected, with low-quality images being discarded. Blob picking was carried out across 1000 micrographs within a diameter range of 110 to 130 Å. The picked particles were extracted and subjected to 2D classification. Well-defined particles were chosen as templates for further template picking, employing a particle diameter of 120 Å across all recorded images. These picked particles were further filtrated by 2D classification and subjected to the “ab-initio reconstruction” job. The best initial model was used for 3D non-uniform refinement and sharpening. The final resolution of the map for the NiV L_1–1,451_-P complex is 2.31 Å.

For the NiV L–P sample dataset (**Fig. S3**), the data processing workflow is the same as that for the NiV L_1–1,451_-P sample dataset, except that a round of 3D classification was performed to remove bad particles. A final NiV L–P map with a resolution of 2.52 Å were obtained (**Fig. S3**).

### Cryo-EM model building and refinement

The coordinates of the NiV P protein (PDB: 6EB8) and an AlphaFold2 (Jumper et al., 2021) model of the NiV L protein by were positioned into the NiV L_1–1,451_-P map as the initial model using UCSF Chimera v1.4 (Pettersen et al., 2004) and COOT v0.9.8.1 (Emsley et al., 2010). The generated structure of NiV L_1–1,451_-P served as the initial models for model building for the NiV L–P map. The structure models were manually built in COOT v0.9.8.1 (Emsley et al., 2010), followed by real-space refinement using PHENIX v.1.20.1 (Afonine et al., 2018). A standard set of stereo-chemical restraints (covalent bonds, angles, dihedrals, planarities, chiralities, non-bonded) and secondary structure restraints were applied with standard settings in the PHENIX program to achieve good model geometry. The data processing and refinement statistics are provided in **Table S1**. The interactions between L and P are summarized in **Table S2**. Structural figures were prepared with UCSF Chimera (Pettersen et al., 2004) or ChimeraX (Pettersen et al., 2021). Interface area analysis was performed using PISA (Krissinel and Henrick, 2007).

## Supplementary Material

pwaf014_suppl_Supplementary_Figures_S1-S20_Tables_S1-S2

## Data Availability

Cryo-EM density maps and coordinates generated in this study have been deposited in the Electron Microscopy Data Bank (EMDB) and the Protein Data Bank (PDB) with the following accession numbers: NiV L_1–1,451_-P complex: EMD-60922 and 9IV9; NiV L–P complex: EMD-60923 and 9IVA. Other data or materials generated in this study are available from the corresponding authors upon request.

## Code availability

This paper does not report original code.
