# Supplementary material for: Cryo-EM structures of Nipah virus polymerase complex reveal highly varied interactions between L and P proteins among paramyxoviruses
Source: Protein Cell. 2025 Feb 18;16(8):705–23. doi: 10.1093/procel/pwaf014 (PMC12342179; doi:10.1093/procel/pwaf014)
Supplement: pwaf014_suppl_Supplementary_Figures_S1-S20_Tables_S1-S2 [file pwaf014_suppl_supplementary_figures_s1-s20_tables_s1-s2.pdf]

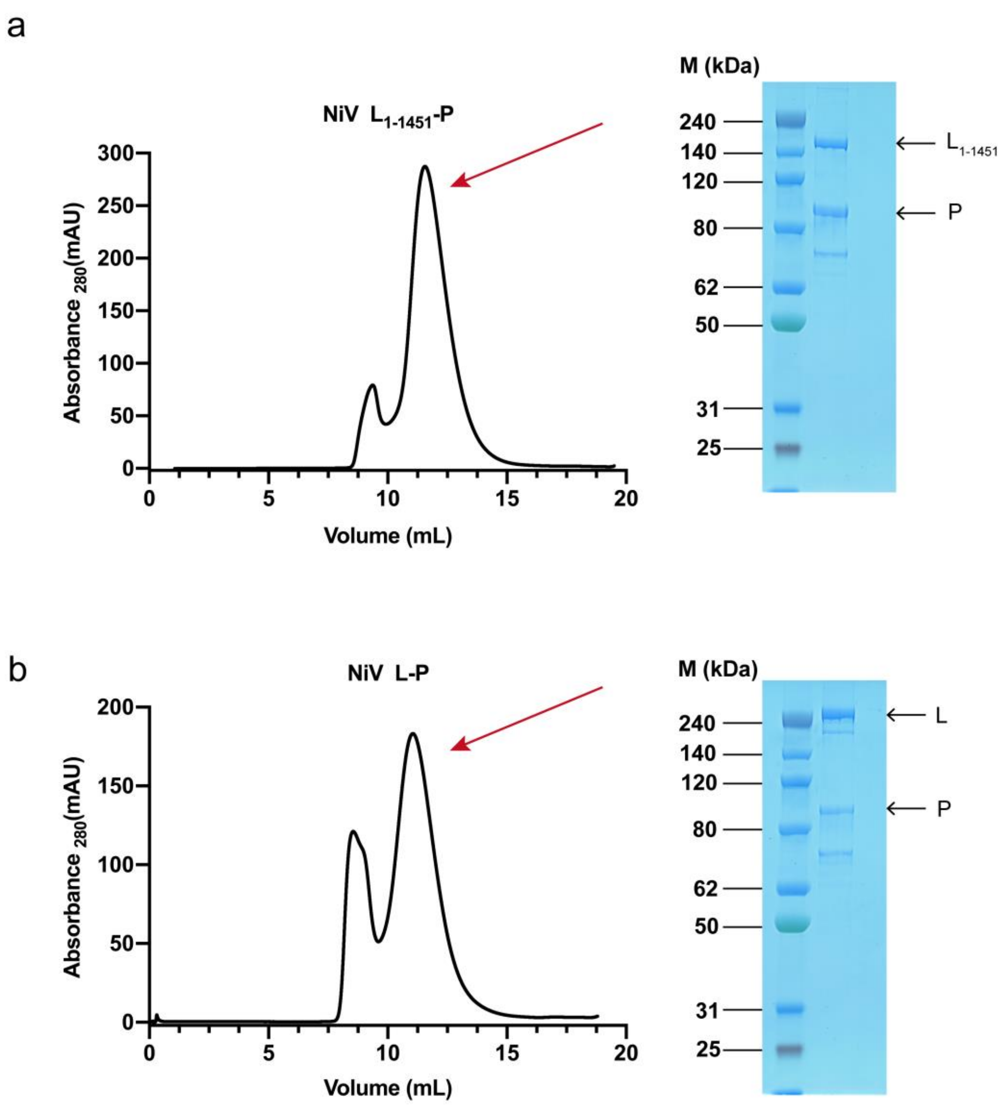

Figure S1 | Purification of the truncated ($L_{1\text{-}1451}$-P) and full-length (L-P) NiV polymerase complexes.

a. Size-exclusion chromatography and Coomassie-stained SDS-PAGE analysis of purified NiV $L_{1\text{-}1451}$-P complex. The molecular weights of the $L_{1\text{-}1451}$ protein and P protein are about 170 kDa and 80 kDa, respectively. **b.** Size-exclusion chromatography and Coomassie-stained SDS-PAGE

analysis of purified NiV L-P complex. The molecular sizes of the L protein and P protein are about 250 kDa and 80 kDa, respectively.

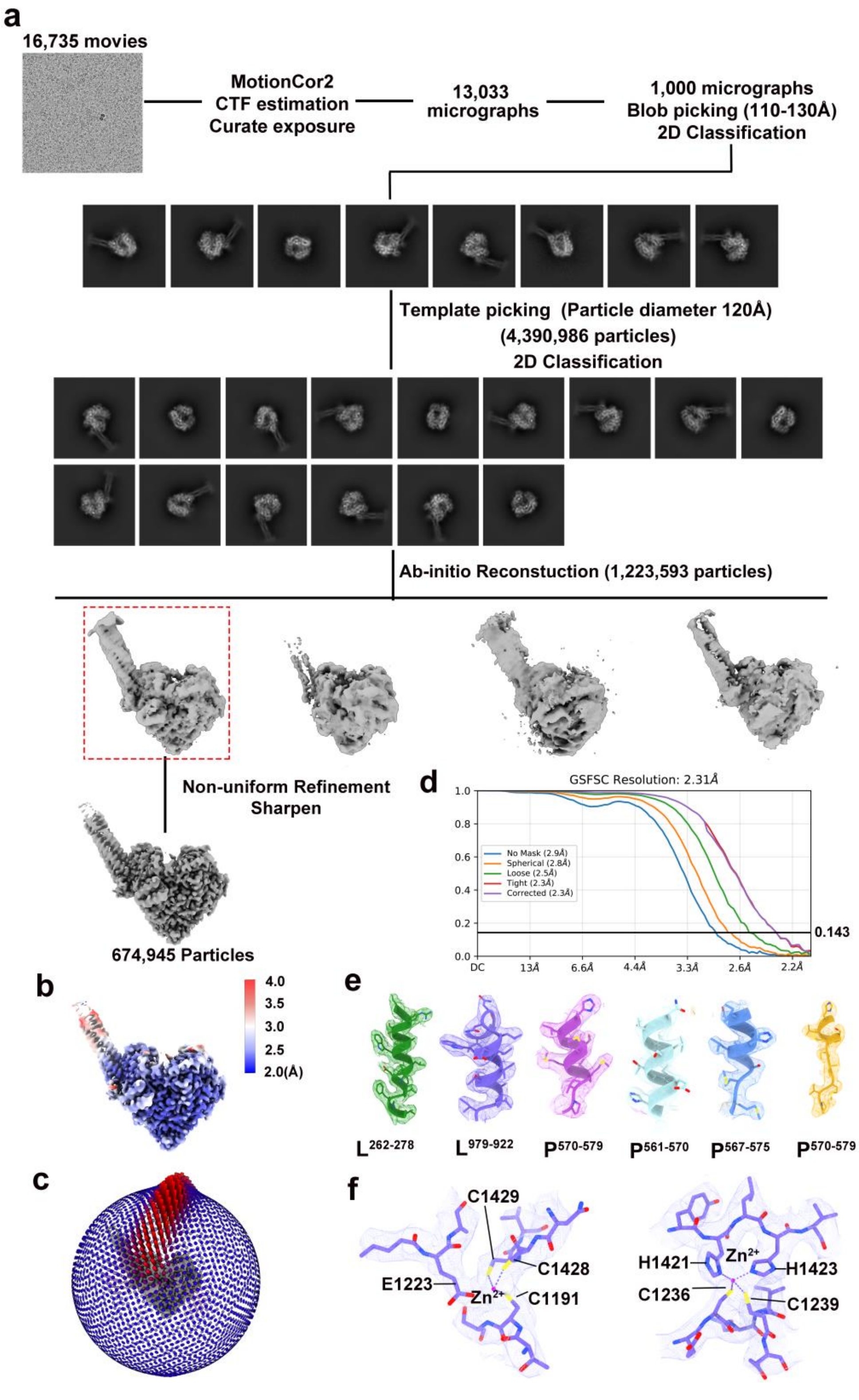

a
16,735 movies
MotionCor2
CTF estimation
Curate exposure
13,033
micrographs
1,000 micrographs
Blob picking (110-130Å)
2D Classification
Template picking (Particle diameter 120Å)
(4,390,986 particles)
2D Classification
Ab-initio Reconstuction (1,223,593 particles)
Non-uniform Refinement
Sharpen
674,945 Particles
b
4.0
3.5
3.0
2.5
2.0(Å)
c
d
GSFSC Resolution: 2.31Å
No Mask (2.9Å)
Spherical (2.8Å)
Loose (2.5Å)
Tight (2.3Å)
Corrected (2.3Å)
0.143
e
L262-278
L979-922
P570-579
P561-570
P567-575
P570-579
f
C1429
C1428
E1223
Zn2+
C1191
H1421
Zn2+
H1423
C1236
C1239

Figure S2 | Cryo-EM data processing and analysis of the NiV $L_{1\text{-}1451}$-P complex.

a. Cryo-EM image processing flowchart for the NiV $L_{1\text{-}1451}$-P complex sample. **b.** A local resolution map for the NiV $L_{1\text{-}1451}$-P complex. **c.** Angular distribution of the particles used for the reconstruction of the NiV $L_{1\text{-}1451}$-P complex structure**. d.** Fourier shell correlation curves of the reconstructed map for the NiV $L_{1\text{-}1451}$-P complex. Overall resolution of the structure was assessed by the gold-standard FSC 0.143 cut-off criteria. **e.** Representative densities from the structure of the NiV $L_{1\text{-}1451}$-P complex. **f.** Cryo-EM densities for the two zinc-binding sites within the PRNTase domain of NiV.

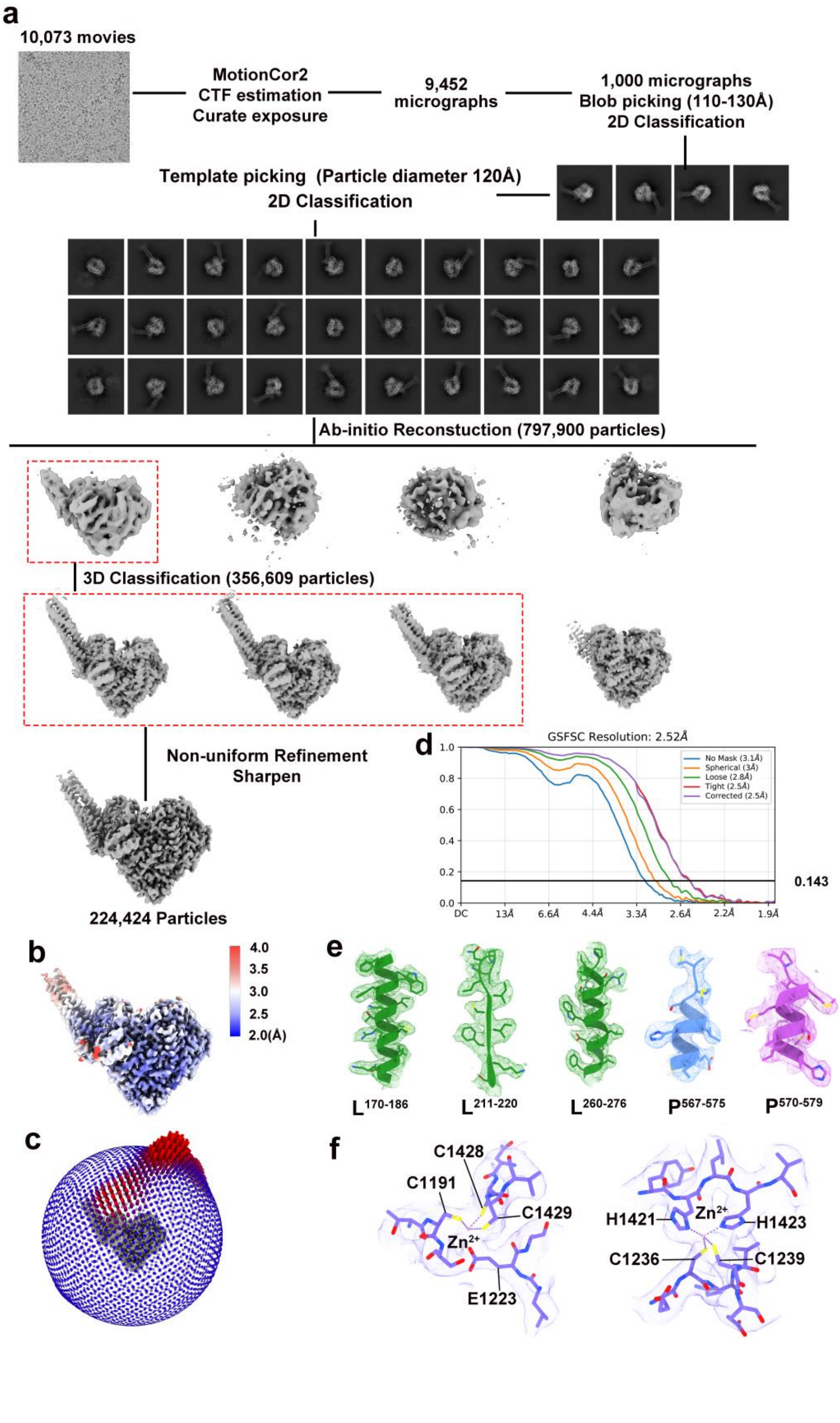
a
10,073 movies
MotionCor2
CTF estimation
Curate exposure
9,452
micrographs
1,000 micrographs
Blob picking (110-130Å)
2D Classification
Template picking (Particle diameter 120Å)
2D Classification
Ab-initio Reconstuction (797,900 particles)
3D Classification (356,609 particles)
Non-uniform Refinement
Sharpen
224,424 Particles
d
GSFSC Resolution: 2.52Å
No Mask (3.1Å)
Spherical (3Å)
Loose (2.8Å)
Tight (2.5Å)
Corrected (2.5Å)
0.143
b
4.0
3.5
3.0
2.5
2.0(Å)
c
e
L170-186
L211-220
L260-276
P567-575
P570-579
f
C1428
C1191
C1429
Zn2+
E1223
H1421
H1423
C1236
C1239

Figure S3 | Cryo-EM data processing and analysis of the NiV L-P complex.

a. Cryo-EM image processing flowchart for the NiV L-P complex sample. **b.** A local resolution map for the NiV L-P complex. **c.** Angular distribution of the particles used for the reconstruction of the NiV L-P complex structure**. d.** Fourier shell correlation curves of the reconstructed map for the NiV L-P complex. Overall resolution of the structure was assessed by the gold-standard FSC 0.143 cut-off criteria. **e.** Representative densities from the structure of the NiV L-P complex. **f.** Cryo-EM densities for the two zinc-binding sites within the PRNTase domain of NiV.

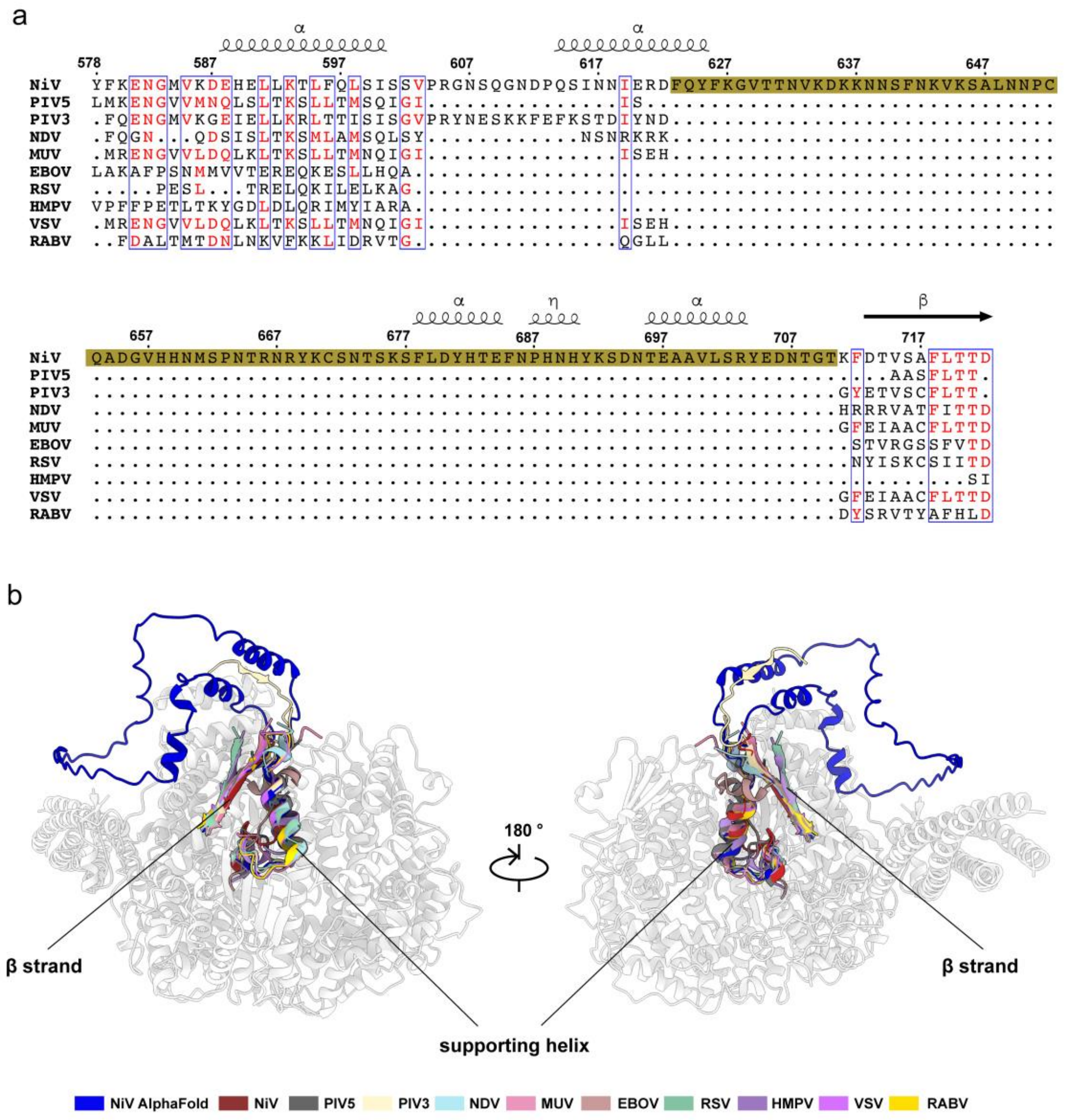

Figure S4 | The RdRp domain of NiV L protein contains a long insertion sequence compared to other NNS RNA virus polymerases.

a. Multiple sequence alignment of polymerase L protein sequences, the long insertion sequence is highlighted in brown. L protein sequences used are NiV (nipah virus), PIV5 (Parainfluenza virus 5), PIV3 (Parainfluenza virus type 3), NDV (Newcastle disease virus), MuV (Mumps virus), EBOV (Ebola virus), RSV (respiratory syncytial virus), HMPV (Human

metapneumovirus), VSV (Vesicular stomatitis virus) and RABV (Rabies virus). **b.** Structural superposition of NiV polymerase complex structure (gary) with an AlphaFold2 predicted long insertion sequence structure (blue) and experimental polymerase strcutures of *Mononegavirales* (colored) indicates that the long insertion sequence (highlighted in brown) form a large loop. This loop is potentially flexible, situated between the supporting helix and a β-sheet in the NiV polymerase.

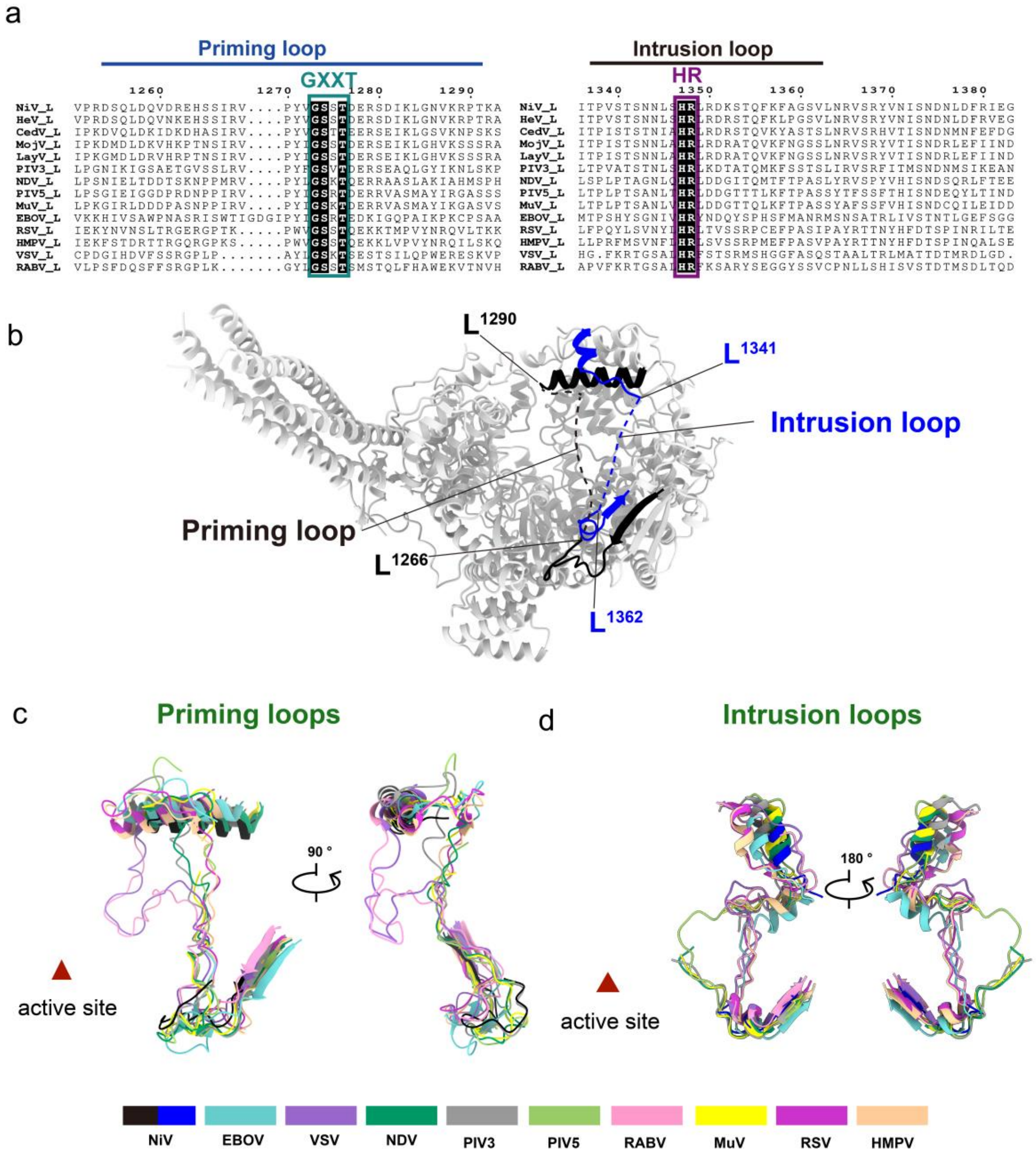

Figure S5 | Structural features of the priming and intrusion loops in NiV polymerase compared to those of other NNS RNA viruses.

a. Multiple sequence alignment of the GXXT and HR motifs in different *Mononegavirales*. The highly conserved GXXT and HR motifs among *Mononegavirales* are highlighted in colored boxes. **b.** The locations of the priming loop and the intrusion loop in the structure of the NiV L-P complex

structure. **c-d.** Comparison of priming (**c**) and intrusion (**d**) loops in the NiV polymerase with those of other *Mononegavirales* shows that the backbone trajectories of residues at both ends of the NiV priming and intrusion loops align well with the conformations observed for HMPV, NDV, and PIV5 polymerases. Polymerase structures used are EBOV (PDB: 8JSM), VSV (PDB: 5A22), NDV (PDB: 7YOU), PIV3 (PDB: 8KDC), PIV5 (PDB: 6V85), RABV (PDB: 6UEB), MuV (PDB: 8IZL), RSV (PDB: 8SNX) and HMPV (PDB: 6U5O). The location of the polymerase catalytic site is indicated by a red triangle.

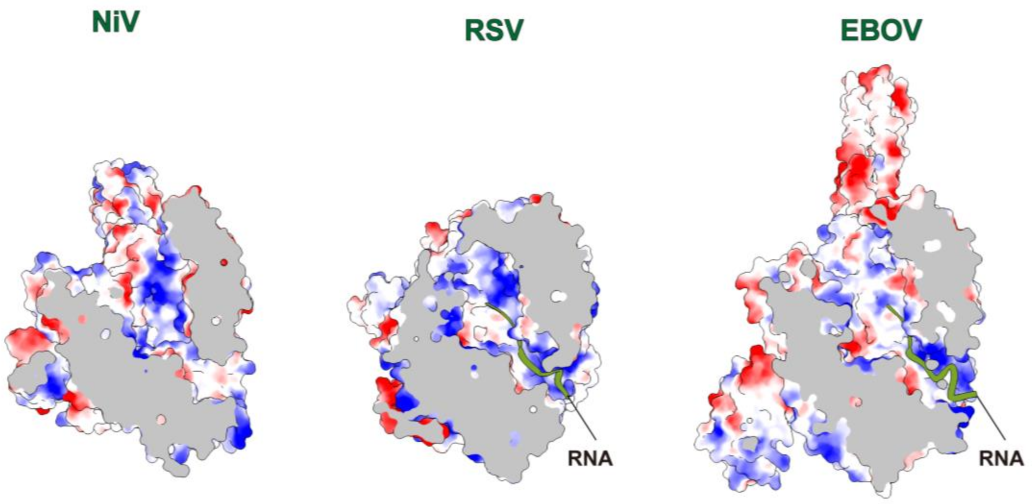

Figure S6 | Polymerase cavity electrostatic potentials compared among NiV, RSV and EBOV.

Cut views to show the electrostatic potentials of NiV, RSV and EBOV polymerase cavities. Bound template RNAs (green) are included for the RSV (PDB: 8SNX) and EBOV (PDB: 8JSM) strcutures. Blue and red colors indicate positive and negative surface charges.

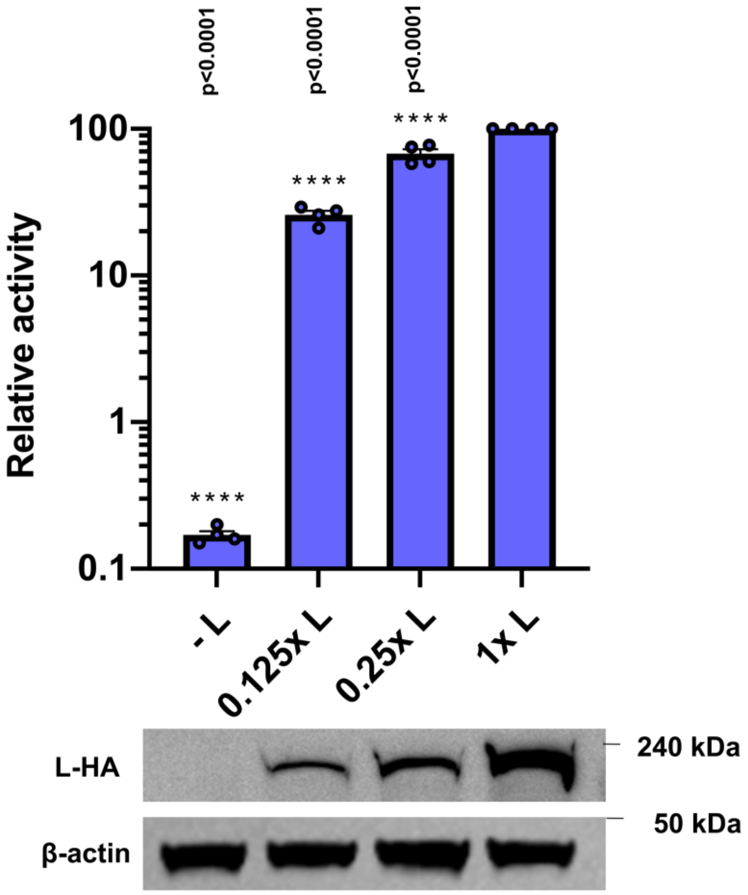

Figure S7 | L protein expression titration experiment.

1×L, 0.25×L, 0.125×L and -L lanes represent protein expression levels in mini-replicon experiments, where 1μg, 0.25 μg, 0.125 μg and 0 μg of L protein expression plasmid were transfected. Corresponding mini-replicon activities are reported as mean ± SEM from four independent experiments (n = 4). All statistics used one-way ANOVA Dunnett's multiple comparisons test. (ns, $p > 0.05$; *, $p < 0.05$; **, $p < 0.01$; ***, $p < 0.001$; ****, $p < 0.0001$).

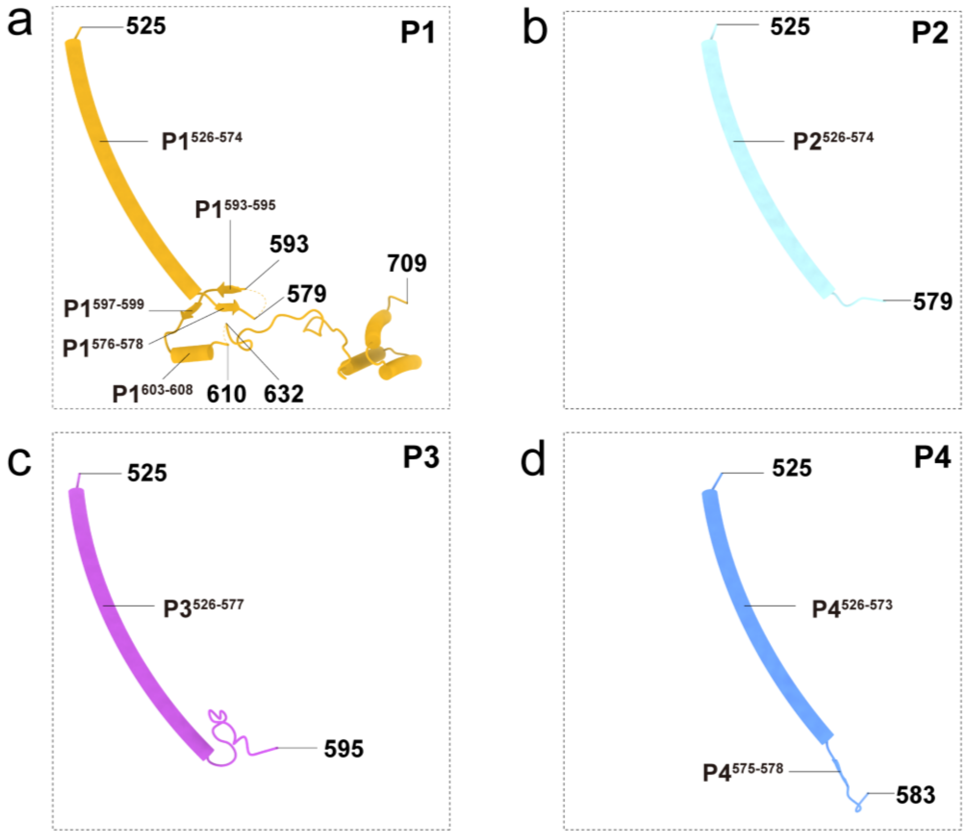

Figure S8 | Structures of P protein monomers within the P protein bundle within the NiV L_{1-1451}-P complex.

a-d. Strcutures of the four P protein monomer – P1 (**a**), P2 (**b**), P3 (**c**) and P4 (**d**) within the L protein bound P protein bundle. Secondary structural elements and their starting and end residues are shown.

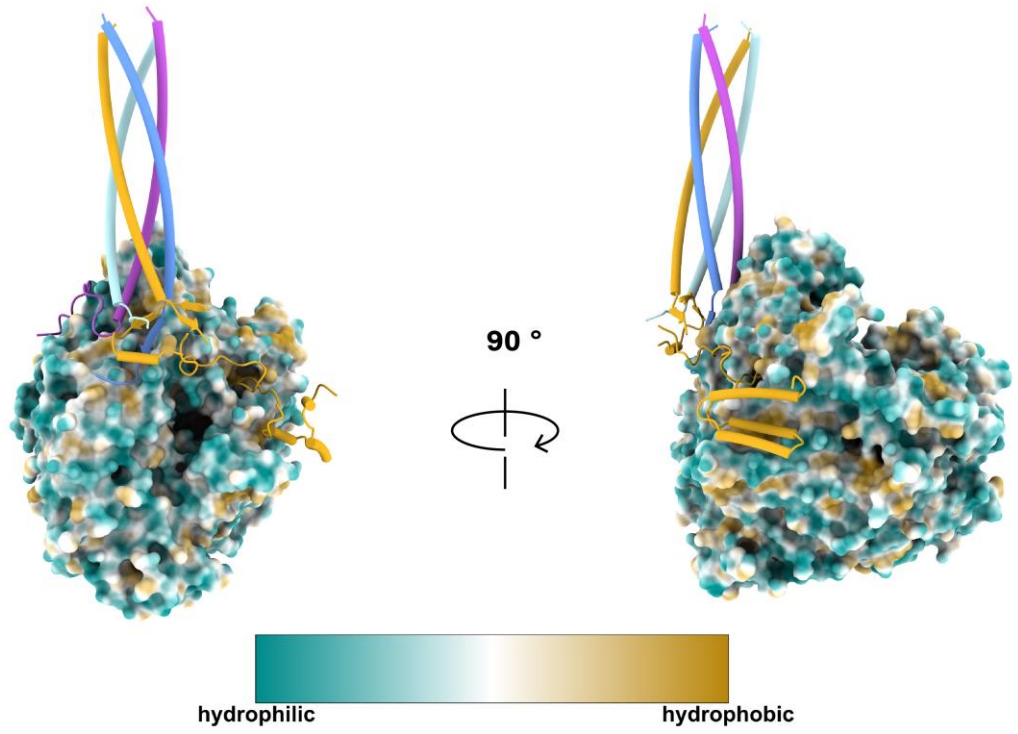

Figure S9 | Binding of the P protein bundle on the L protein surface.

The surface of the L protein is coloured according to the hydrophobicity of its surface residues. The P1, P2, P3 and P4 monomers of the P protein bundle are colored yellow, cyan, magenta and blue, respectively.

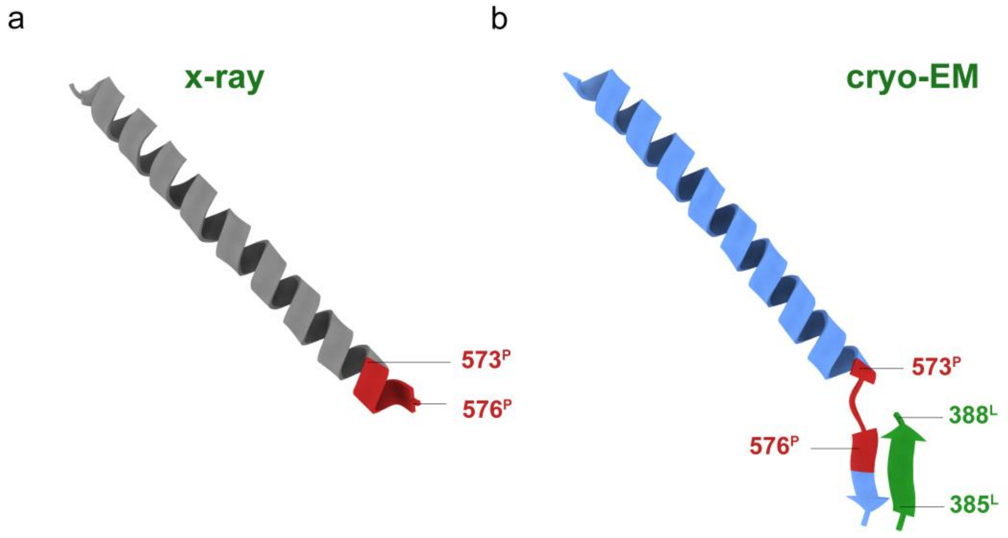

Figure S10 | Conformational change of the NiV P protein upon L protein binding.

a. An α-helix extracted from the X-ray structure of the NiV P protein tetramer. Residues 573-576 at the C-terminal end of the helix is shown in red. **b.** The structure of P4 within the P protein tetramer in the cryo-EM structure. Upon L binding, the residues ($P^{573\text{-}576}$, red) refold to transform into a loop and part of a β-strand to bind $L^{385\text{-}388}$, forming an antiparallel sheet.

```
NiV_L           -------MADELSISDIIYPECHLDSPIVSGKLISAIEYAQL-------RHNQPSDDKRL   46
NDV_L           ----MAGSGSERAEHQIILPESHLSSPLVKHKLLYYWKLTGL-------PLPDECDFDHL   49
RSV_L           MDPIINGNSA-----NVYLTDSYLKGVISFS-------ECNALGSYIFNGPYLKNDYTNL   48
EBOV_L          -----------MATQHTQYPDARLSSPIVLDQCDLVTRACGLYSSYSLNPQLRNCKLPKH   49
                               .    :. *.. :                           .  .
NiV_L           --SENIRLNLHGKRKSLYILRQSKQGDYIRNN-IKN-LKEFMHIAYPECNNILF------   96
NDV_L           ILSRQWKKILES--STPDIERMIKLGRSVHQT-LSH-SSKLTGILHPRCLEDLV------   99
RSV_L           ISRQ-----------NPLIEHMNLKKLNITQSLISKYHKGEIKLEEPTYFQSLLMTYKSM   97
EBOV_L          IYRLKYDVTVTKFLS--DVPVATLPIDFIVPILLKALSGNGFCPVEPRCQQFLD------   101
                                  :         :    :.           *   : *

NiV_L           -----------------------SITSQGMTSKLDNIMKKSFKAYNIISKKVIGMLQNIT   133
NDV_L           -----------------------GLDIPDSTNKFRRIEKKIQIHNTRYGEPFTRLCSYVE   136
RSV_L           TSSEQIATTNLLKKIIRRAIEISDVKVYAILNKLGLKEKDKIKSNNGQDE---------D   148
EBOV_L          -----------------------EIIKYTMQDA-------LFLKYYL--KN-VGA----Q   124
                                        :      .                 :

NiV_L           RNLITQDRRDEIINI----------------HEC-----RRLGDLGKNM-SQSKWYECFL   171
NDV_L           KKLLGSSWTHKIRRS----------------EEF-----DSLRTDPAFW-FHSSWSTAKF   174
RSV_L           NSVITTIIKDDILSAVKDNQSHLKADKNHSTKQKDTIKTTLLKKLMCSMQHPPSWL---I   205
EBOV_L          EDCVDDHFQEKILSS---------------------------------I-QGNEFLHQMF   150
                .. :     ..*                                         .:    :

NiV_L           FWFTIKTEMRAVIKNS---QKPK-FRSDSCIIHMRDKSTEIILNPNL-------------   214
NDV_L           AWLHVKQIQRHLIVAA---RTRS---ASNKLVTLSHRSGQVFITPEL-------------   215
RSV_L           HWFNLYTKLNNILTQY---RSNEVKNHGFTLIDNQTLSGFQFILNQ--------------   248
EBOV_L          FWYDLAILTRRGRLNRGNSRSTW-FVH-DDLIDILGYGDYVFWKIPISLLPLNTQGIPHA   208
                 *  :    .         :.         ::     .   :

NiV_L           ------ICIFKSDKTGK--KCYYLTPEMVLMYCDVLEGRMMMETTVKSDIKYQPL-----   261
NDV_L           ------VIVTHTNEN----KFTCLSQELVLMYADMMEGRDMVNIISSTAVHLRCL-----   260
RSV_L           ----YGCIVYHKELK----RITVTTYNQFLTWKDISLSRLNVCLITWISNCLNTLNKSLG   300
EBOV_L          AMDWYQTSVFKEAVQGHTHIVSVSTADVLIMCKDLITCRFNTTLISKIAEVEDPVCSDY-   267
                        : :             : : .:   *:   *               :

NiV_L           ------ISRSNALWGLIDPLFPVMGNRIYNIVSMIEPLVLALLQLKDEARILRGAFLHHC   315
NDV_L           ------AEKIDDILRLVDALARDLGNQVYDVVALMEGFAYGAVQLLEPSGTFAGDFFSFN   314
RSV_L           LRCGFNNVILTQLFLYGDCILKLFHNEGFYIIKEVEGFIMSLILNITEEDQFRKRFYNSM   360
EBOV_L          ----PNFKIVSMLYQSGDYLLSILGSDGYKIIKFLEPLCLAKIQLCSKYTERKGRFLTQM   323
                            :    * :   : .  : ::  :* :  . :            *

NiV_L           IKEMHQELSECG----FTD---Q-----KIRSMF-----------IDDLLSILNIDNIHL   352
NDV_L           LQELRDTLI-CL----L-P---Q-----RIADSV-----------THAIANIFSGLEQNQ   349
RSV_L           LNNITDAANKAQKN--LLSRVCHTLLDKTVSDNIINGRWIILLSKFLKLIKLAGDNNLNN   418
EBOV_L          HLAVNHTLEEITEIRALKPSQAH-----KIREFH------------R--TLIRLEMTPQQ   364
                   : .          :     :      : .                   :      :

NiV_L           LAEFFSFFRTFGHPILEAKVAAEKVREHMLADKVLEYAPIMKAHAIFCGTIINGYRDRHG   412
NDV_L           AAEMLCLLRLWGHPLLESRAAAKAVRAQMCAPKMVDFDMILQVLSFFKGTIINGYRKKNA   409
RSV_L           LSELYFLFRIFGHPMVDERQAMDAVKINCNETKFYLLSSLSMLRGAFIYRIIKGFVNNYN   478
EBOV_L          LCELFSIQKHWGHPVLHSETAIQKVKKHATVLKALRPIVIFETYCVFKYSIAKHYFDSQG   424
                 .*:  : : :***::. . * . *: :    *      :      *   * : : .

NiV_L           GAWPPLYLPAHASKHIIRLKNSGESLTIDDCVK-NWESFCGIQFDCFMELKLDSDLSMYM   471
NDV_L           GVWPRVKAHTIYGNVIAQLHADSAEISHDIMLR-EYKNLSAIEFEACIEYDPVTNLSMFL   468
RSV_L           -RWPTLRNAIVLPLRWLTYYKLNTYPSLLELTERDLIVLSGLRFYREFRLPKKVDLEMII   537
EBOV_L          -SWYSVTSDRNLTPGLNSYIKRNQ-FPPLPMIKELLWEFYHLDHPPLFSTKIISDLSIFI   482
                  *  :                .         .     :  :  .   :      :*.: :

NiV_L           KDKALSPIKDEWDSVYPREVLSYTPP-------K----STEPRRLVDVFVNDENFDPYNM   520
NDV_L           KDKAIAHPRNNWLASFRRNLLSEEQK-------KNVQDSTSTNRLLIEFLESNDFDPYKE   521
RSV_L           NDKAISPPKNLIWTSFPRNYMPSHIQNYIEHEKLKFSESDKSRRVLEYYLRDNKFNECDL   597
EBOV_L          KDRATAVERTCWDAVFEPNVLGYNPP-------H----KFSTKRVPEQFLEQENFSIENV   531
                :*:* :  :    : :  : :                 . . .*:   ::..:.*.  .

NiV_L           LEYVLSGAYLEDEQFNVSYSLKEKETKQAGRLFAKMTYKMRACQVIAEALIASGVGKYFK   580
NDV_L           MEYLTTLEYLRDDSVAVSYSLKEEEVKVNGRIFAKLTKKLRNCQVMAEGILADQIAPFFQ   581
RSV_L           YNCVVNQSYLNNPNHVVSLTGKERELS-VGRMFAMQPGMFRQVQILAEKMIAENILQFFP   656
EBOV_L          LSYAQKLEYLLPQYRNFSFSLKEKELN-VGRTFGKLPYPTRNVQTLCEALLADGLAKAFP   590
                 .   .   **     .* :    **.* .   ** *.  *  * :.* ::*. :   *

NiV_L           ENGMVKDEHELLKTLFQLSISSVPRGNSQGNDPQSINNIERDFQYFKGVTTNVKDKKNNS   640
NDV_L           GNGVIQDSISLTKSMLAMSQLSYNSNRK--------------------------------   609
RSV_L           ESLTRYGDLELQKILELKAGISNK------------------------------------   680
EBOV_L          SNMMVVTEREQKESLLHQAS----------------------------------------   610
                 .     . .  : :   :

NiV_L           FNKVKSALNNPCQADGVHHNMSPNTRNRYKCSNTSKSFLDYHTEFNPHNHYKSDNTEAAV   700
NDV_L           --------------------------R-------------------------ITDCKERVS   619
RSV_L           ------------------------------------------------------------   680
EBOV_L          ------------------------------------------------------------   610
```

```
NiV_L        LSRYEDNTGTKFDTVSAFLTTDLKKFCLNWRYESMAIFAERLDEIYGLPGFFNWMHKRLE     760
NDV_L        SSRNHDLKGKHRRRVATFITTDLQKYCLNWRYQTIKLFAHAINQLMGLPHFFEWIHLRLM     679
RSV_L        SNRYNDNYNN--YISKCSIITDLSKFNQAFRYETSCICSDVLDELHGVQSLFSWLHLTIP     738
EBOV_L       WHHTSDDFGEHATVRGSSFVTDLEKYNLAFRYEFTAPFIEYCNRCYGVKNVFNWMHYTIP     670
               :  *   .           : ***.*:   :**:        .  :.   *:   .*.*:*   :

NiV_L        RSVIYVADPNCPPNID-KHMELEKTPEDDIFIHYPKGGIEGYSQKTWTIATIPFLFLSAY     819
NDV_L        DTTMFVGDPFNPPSDP-TDYDLTKVPNDDIYIVSARGGIEGLCQKLWTMISIAAIQLAAA     738
RSV_L        HVTIICTYRHAPPYIGDHIVDLNNVDEQSGLYRYHMGGIEGWCQKLWTIEAISLLDLISL     798
EBOV_L       QCYMHVSDYYNPPHNL-TLENRNNPPEGPSSYRGHMGGIEGLQQKLWTSISCAQISLVEI     729
                :        **        :  :  :          *****  ** **   :    :  *

NiV_L        ETNTRIAAIVQGDNESIAITQKVHPNLPYKVKKEICAKQAQLYFERLRMNLRALGHNLKA     879
NDV_L        RSHCRVACMVQGDNQVIAVTREVRPDDSPESVLTQLHEASDNFFRELIHVNHLIGHNLKD     798
RSV_L        KGKFSITALINGDNQSIDISKPIRLMEGQTHAQA-DYLLALNSLKLLYKEYAGIGHKLKG     857
EBOV_L       KTGFKLRSAVMGDNQCITVLSVFPLETDAGEQEQSAEDNAARVAASLAKVTSACGIFLKP     789
             .    :  . : ***: *  :    .                   :       *        *   **

NiV_L        TETIISTHLFIYSKKIHYDGAVLSQALKSMSRCCFWSETLVDETRSACSNISTTIAKAIE     939
NDV_L        RETIRSDTFFIYSKRIFKDGAILSQVLKNSSKLVLVSGDLSENTVMSCANISSTVARLCE     858
RSV_L        TETYISRDMQFMSKTIQHNGVYYPASIKKVLRVGPWINTILDDFKVSLESIGSLTQELEY     917
EBOV_L       DETFVHSGFIYFGKKQYLNGVQLPQSLKTATRMAPLSDAIFDDLQGTLASIGTAFERSIS     849
              **       :    .*     :*.      :*.   :          :  ::    :   .*.:     .

NiV_L        NGLSRNVGYCI----NILKV-------IQQLLISTEFSINETLTL---DVTSPISNNLDW     985
NDV_L        NGLPKDFCYYL----NYLMS-------CIQTYFDSEFSITSSTQS---GSNQSWINDIPF     904
RSV_L        RGESLL-CSLI--FRNVWLYNQIALQLKNHALCNNKLYLDILKVLKHLKTFFNLDNIDTA     974
EBOV_L       ETRHIFPCRITAAFHTFFSVRIL---QYHHLGFNKGFDLGQL-TL---G----KPLDFGT     898
             .                .                  :     .. : :

NiV_L        LITAALIPAPIGGFNYLNLSRIFVRNIGDPVTASLAD----LKRMIDHSIM------TES     1035
NDV_L        IHSYVLTPAQLGGLSNLQYSRLYTRNIGDPGTTAFAE----VKRLEAVGLL------GPN     954
RSV_L        LTLYMNLPMLFGGGDPNLLYRSFYRRTPDFLTEAIVHSVFILSYYTNHDLKDKLQDLSDD     1034
EBOV_L       ISLALAVPQVLGGLSFLNPEKCFYRNLGDPVTSGLFQ----LKTYLRMIEM------D-D     947
             :       *   :** .      :  :  *.    *   *  .:  .      :.                   .

NiV_L        VL-----QKVMNQEPGDASFLDWASDPYSGNLPDSQSITKTIKNITARTILRNSPNPMLK     1090
NDV_L        IM-----TNILTRPPGNGDWASLCNDPYSFNFESVASPSIVLKKHTQRVLFETCSNPLLS     1009
RSV_L        RLNKFLTCIITFDKNPNAEFVTLMRDPQALGSERQAKITSEINRLAVTEVLSTAPNKIFS     1094
EBOV_L       LF-----LPLIAKNPGNCTAIDFVLNPSGLNVPGSQDLTSFLRQIVRRTITLSAKNKLIN     1002
              :         :         :          :*  .  .       .  :  :.. .     :    .. *  ::.

NiV_L        GLFHDKSFDEDLELASFLMDRRVILPRAAHEILDNSLTGAREEIAGLLDTTKGLIRSGLR     1150
NDV_L        GVHTEDNEAEEKALAEYLLNQEVIHPRVAHAIMEASSVGRRKQIQGLVDTTNTVIKIALS     1069
RSV_L        KSAQHY-TTTEIDLNDIMQNIEPTYPHGLRVVYESLPFYKAEKIVNLISGTKSITNILEK     1153
EBOV_L       TLFHASADFEDEMVCKWLLSSTPVMSRFAADIFSRTPSGKRLQILGYLEGTRTLLASKII     1062
                        :   :  .  :  .          :        :   .            :*  .  :.  *.  :

NiV_L        KSGLQPKLVSRLSHHDYN--Q-FLILNKLLSNRRQNDLISSN-TCSVDLARALRSHMWRE     1206
NDV_L        RKPLGIKRLARIINYSSMH-AMLFRDDVFLSNRANHPLVSSD-MCSLALADYARNRSWSP     1127
RSV_L        TSAIDLTDIDRATEMMRKNITLLIRILPLDCNRDKREILSMENLSITELSKYVRERSWSL     1213
EBOV_L       NNNTETPVLDRLRKITLQR-WSLWFSYLDHCDNILAEALTQI-TCTVDLAQILREYSWAH     1120
               .         : *   .            :          .:.       ::      .      *:      *.   *

NiV_L        LALGRVIYGLEVPDALEAMVGRYITGSLECQICEQGNTMYGWFFVPRDSQLDQV------     1260
NDV_L        LTGGRKILGVSNPDTIELVEGEILSISGGCSKCDSGDEQFTWFHLPSNIELTDD------     1181
RSV_L        S----NIVGVTSPSIMYTMDIKYTTSTISSGI--------------------IIEKYNVN     1249
EBOV_L       ILEGRPLIGATLPCMIEQFKVVWLKPYEQCPQCSNAKQPGGKPFVSVAVKKHIVSAWPNA     1180
                     : *    *   :   .       .      .

NiV_L        --DREHSSIRVPYVGSSTDERSDIKLGNVKRPTKALRSAIRIATVYTWAYGDNEECWYEA     1318
NDV_L        --TSKNPPMRVPYLGSKTQERRAASLAKIAHMSPHVKAALRASSVLIWAYGDNDINWTAA     1239
RSV_L        SLTRGERGPTKPWVGSSTQEKKTMPVYNRQVLTKKQRDQIDLLAKLDWVYASIDNKDEFM     1309
EBOV_L       SRISWTIGDGIPYIGSRTEDKIGQPAIKPKCPSAALREAIELASRLTWVTQGSSNSDLLI     1240
                          *::**  *:::         :      :      :    :      :     *.    .  .

NiV_L        WYLASQRVNIDLDVLKAITPVSTSNNLSHRLRDKSTQFKFAGSVLNRVSRYVNISNDNLD     1378
NDV_L        LKLARSRCNISSEYLRLLSPLPTAGNLQHRLDDGITQMTFTPASLYRVSPYVHISNDSQR     1299
RSV_L        EELSIGTLGLTYEKAKKLFPQYLSVNYLHRLTVSSRPCEFPASIPAYRTTNYHFDTSPIN     1369
EBOV_L       KPFLEARVNLSVQEILQMTPSHYSGNIVHRYNDQYSPHSFMANRMSNSATRLIVSTNTLG     1300
                :        .:   :       :  *     :  *    **             *            :       ....

NiV_L        FRIEGE--KVDTNLIYQQAMLLGLSVLEGKFRLRLETDDYNGIYHLHVKDNCCVKEVADV     1436
NDV_L        LFTEEG--VKEGNVVYQQIMLLGLSLIESLFPMTVTKTYDEITLHLHSKFSCCIREAPVA     1357
RSV_L        RILTEKYGDEDIDIVFQNCISFGLSLMSVVEQFTNVCPN---RIILIPKLNEIHLMKPPI     1426
EBOV_L       EFSGGGQSARDSNIIFQNVINYAVALFDIKFRNTEATDIQYNRAHLHL-TKCCTREVPAQ     1359
                          :  ::::*:  :    .::::.                              *      .

NiV_L        GQVDAELPIPEYTEVDNNHLIYDPDPVSEIDCSRL--SNQESKSRE-LDFPLWSTEELHD     1493
NDV_L        VPFELTGVAPDLRVVASNKFMYDPNPVAEGDFARL--DLAIFKSYE-LNLESYSTVELMN     1414
RSV_L        --FTGDVDIHKLKQVIQKQHMFLPDKISLTQYVELFLSNKTLKSGSHVNS---NLILAHK     1481
EBOV_L       YLTYTSTLDLDLTRYRENELIYDNNPLKGGLNCNISFDNPFFQGKQ-LNIIEDDLIRLPH     1418
                          .      .:. ::   : :       .:   .      :.  .  ::     .      .
```

```
NiV_L       V---------LAKTVAQ-TVLE-IITKADKDVLKQHLAIDSDDNINSLITEFLIVDPELF    1542
NDV_L       I---------LSISSGK-LIGQ-SVVSYDEETSIKNDAIIVYDNTRNWISEAQNSDVVRL    1463
RSV_L       ISDYFHNTYILSTNLAGHWILIIQLMKDSK-------GIFEKDWGEGYITDHMFINLKV-    1533
EBOV_L      L---------SGWELAK-TIMQ-SIISDSNNSST---DPISSGETRSFTTHFLTYPKIGL    1464
            :          .   .   :    : . .:            . ..   :.

NiV_L       ALYLGQSISIKWAFEIHHRRPRGRHTMVDLL--SDLVSN---TSKHTYKVLSNALSHPRV    1597
NDV_L       FEYAALEVLLDCSYQLYYLRVRGLNNVVLYM--SDLYKN---MPGILLSNIAATISHPII    1518
RSV_L       --FFNAYKTYL----LCFHKGYGKAKLECDMNTSDLLCVLELIDSSYWKSMSKVFLEQKV    1587
EBOV_L      LYSFGAFVSYYLGNTILRTKKLTLDNFLYYL--TTQIHN---LPHRSLRILKPTFKHASV    1519
                           :   :     ..   :  :                :  .: .  :

NiV_L       FKRFVNCGLLLP------TQGPYLHQQDFEKLSQNLLVTSYMIYLMNWCDFKK--SPFLI    1649
NDV_L       HSRLHTVGLISH------DGSHQLADTDFIELSAKLLVSCTRRVVSGLYAGNK--YDLLF    1570
RSV_L       IKYILSQDASLH-------------------------------RVKGC-----HSFKLWF    1611
EBOV_L      MSRLMSIDPHFSIYIGGAAGDRGL--SDAARLFLRTSISSFLTFVKEWIINRGTIVPLWI    1577
             . : . .                                    :            : :

NiV_L       AEQDE----TVISLREDII-------TSKHLCVIIDLYANHHKPPWIIDLNPQ--EKICV    1696
NDV_L       PSVLD----DNLNEKMLQL-------ISRLCCLYTVLFATTREIPKIRGLPAE--EKCAM    1617
RSV_L       LKRLN--------------------------------VAEFTVCPWVVNIDYHPTHMKAI    1639
EBOV_L      VYPLEGQNPTPVNNFLHQIVELLVHDSSRHQAFKTTIND--H-------VHPH-------    1621
                :                                            :  .

NiV_L       LRDFISKSRHVD--TSS--RSWNTSDLDFVIFYASLTYLRRGIIKQLR------------    1740
NDV_L       LTEYLLSDAVRPLLSPE--QVDSITSPSIVTFPANLYYMSRKSLNLIR------------    1663
RSV_L       LT-YIDLVRM-GLINIDRIHIKNKHKFNDEFYTSNLFYINYNFSDNTH-LLTKHIRIANS    1696
EBOV_L      ---------------DN--LVYTCKSTASNFFHASLAYWRSRHRNSNRKDLTRNSSTG-S    1663
                            .     .  .     : :.* *      .  :

NiV_L       -----------------IRQVTEVIDT------T----------TMLRDNIIVENPPIK-    1766
NDV_L       -----------------EREDRDSILA------L-----------MFPQEPLFEFPLVQD    1689
RSV_L       ELENNYNKLYHPTPETLENILANPIKSNDKKTLNDYCIGKNVDSIMLPLLS--NKKLIK-    1753
EBOV_L      STNNSDGHIK----RSQEQTTRDPHDGTERSLVL--QMSHEIKRTTIPQENTHQGPSFQ-    1716
                              .   :                       :      :   .:

NiV_L       ----------------TGVLDIRGCIIYNLEE--------ILSMNTKSASK---------    1793
NDV_L       IGARVKDQLTMKPAAFLHELDLSAPARYDAYT--------LE---QAR-SD---------    1728
RSV_L       ------------SSAM-IRTNYSKQDLYNLFPMVVIDR--IIDHSGNTAKS---NQ----    1791
EBOV_L      -----------------SFLSDSACGTANPKLNFDRSRHNVKSQDHNSASKREGHQIISH    1759
                                .       :           :        ..

NiV_L       -------------KIFNL----NSRPSVEN------------------HKYRRIGLNSSS    1818
NDV_L       -------------CALAD----MGEDQLVR------------------YLFRGVGTASSS    1753
RSV_L       --------------LYTT---TSHQISLVHNSTSLYCMLPWHHINRFNFVFSSTGCKISI    1834
EBOV_L      RLVLPFFTLSQGTRQLTSSNESQTQDEISKYLRQLRSVI------DTTVYCRFTGIVSSM    1813
                                    . .: .                        *   *

NiV_L       CYKALNLSPLIQRYLPSGAQRLFIGEGSGSMMLLYQSTLGQSISFYN-SGIDGDYIPGQR    1877
NDV_L       WYKASHLLSVPEIRCARHGNSLYLAEGSGAIMSLLELHIPHETIYYN-TLFSNEMNPPQR    1812
RSV_L       EYILKDL------KIKDPNCIAFIGEGAGNLLLRTVVELHPDIRYIYRSLKDCNDH----    1884
EBOV_L      HYKLDEVLWEIEN--FKSAVTLAEGEGAGALLL--IQKYQVKTLFFN-TLATESSIESEI    1868
             * .:                   .**:* ::         .  :   :    .

NiV_L       ELK--------LFPSEYSIAEEDPSLTGKLKGLVVPLFNGRPETTWIGNLD---------    1920
NDV_L       HFG--------PTPTQFLNSVVYRNLQAEVP--CKDGFVQEFRTLWRENTEE--SDLTSD    1860
RSV_L       -----------SLPIEFL-----------------RLYNGHINIDYGENLTIPATDATNN    1916
EBOV_L      VSGMTTPRMLLPVMSKFHNDQIEIILNNSA-----SQITDITNPTWFKDQRA--------    1915
                           ::                         .  :  :

NiV_L       -SYEYIINRTAGRSIGLVHSDMESGIDKNVEEILVEHSHLISI-AINVMMEDGLLVSKIA    1978
NDV_L       KAVGYITSVVPYRSVSLLHCDIEIPPGSNQSLLDQLATNLSLI-AMHSVREGGVVIVKIL    1919
RSV_L       IHWSYL-HIKFAEPISLFVCDAELSVTVNWSKIIIEWSKHVRKCKYCSSVNKCMLIVKYH    1975
EBOV_L      ---------RLPRQVEVITMDAETTENINRSKLYEAVHKLILH-HVDPSVLK-AVVLKVF    1964
                        . : :.  * *     * . :     :               :: *

NiV_L       YTPGFPISRLFNMYRSYFGLVLVCFPVYSNPDSTEVYLLCLQKTV------KTIVPPQK-    2031
NDV_L       YSMGYYFHLLVNLFTPCSVKGYVLSNGYACRGDMECYVVFVMGYLGGPTFVNEVVRMAKT    1979
RSV_L       AQDDID--FKLDNIT--ILKTYVCLG--SKLKGSEVYLVLTIGPANIFPV-FNVVQNAKL    2028
EBOV_L      LSDTEGMLWLNDNLAPFFATGYLIKPITSSARSSEWYL-CLTNFLSTTRK----MPHQNH    2019
                     :            :     :   . * *:                :   :

NiV_L       -VLEHSNLHDEVNDQGITSVIFKIKNSQSKQFHDDLK---------KYYQIDQPFFV-PT    2080
NDV_L       LIQRHGTLLAKSDETALMA-LF---TSQKQRVDNILS-------------SPLPRLA-KL    2021
RSV_L       ILSRTKNFI-------MP------KKADKESIDANIK-------------SLIPFLCYPI    2062
EBOV_L      LS---------CKQVILTALQLQIQR--SPYWLSHLTQYADCDLHLSYIRLGFPSLE-KV    2067
                            :           .      :.                 * :

NiV_L       KITSDEQVLLQAGLKLNGPEILKS---------EISYDIGSDINTLRDTIIIMLNEAMNY    2131
NDV_L       LRRNIDTALIEAGGQPVRPFCAESLVNTLSDITQTTQVIASHIDTVIRSVIYMEAE----    2077
RSV_L       TKKGINTALSKLKSVVSGDILSYSIAGRN-EVFS-----NKLINHKHMNILKWFNHVLNF    2116
EBOV_L      LYHRYNLVDSK-----RGPLVS---------V-------TQHLAHLRAEIRELTND-YNQ    2105
                 : .  :                             . :      :     .
```

```
NiV_L       FDDNRSPSHHLEPYPVLERTRIKTIMNCVTKKVIVYSLIKFKDT-------------KSS    2178
NDV_L       -GDLADTVFLFTPYNLSIDGKKRTSLKQCTRQILEVTILGLGPE-------------DLN    2123
RSV_L       RS----TELNY-------------------------------------------------    2123
EBOV_L      QRQSRTQTYHF---IRTAKGRITKLVNDYLKFFLIVQALKHNGTWQAEFKKLPELISVCN    2162

NiV_L       ELYHIKNNIRRKVL----ILDFRSKLMTKTLPKGMQERRE----KNGFKEVWIVDLSNRE    2230
NDV_L       RVGDIISLILRGTISLEDLIPLRTYLKMSTCPKYLKSVLGLTKLREMFSDGSMLYLTRAQ    2183
RSV_L       ----------NHLYMVESTYPYLSELLNSLTTNELKKLIKITGS--------LLYNFHNE    2165
EBOV_L      RFYHIRDCNCEERFLVQTL-----YLH-RMQDSEVKLIERLTGLLSLFPDGLYRFD----    2212
                      .              *       . ::

NiV_L       VKIWWKIIGY-----ISII--     2244
NDV_L       QKFYMKTVGNAVKGYYNSSKN     2204
RSV_L       ---------------------     2165
EBOV_L      ---------------------     2212
```

Figure S11 | Aligment of NiV, NDV, RSV, and EBOV L protein sequences.

Hydrophobic or aromatic residues contacted by P1 (yellow), P2 (cyan), P3 (magenta) and P4 (blue) monomers of the P tetramer are shade according to the P monomer they contacted with. Residues contacted by two P monomers are indicated by both shades and colored letters. Residues contacted by three P monomers are indicated by shades, colored letters, and underlines.

Total BSA = 3537 Å^2

>NiV_P1 (BSA = 1743 Å^2)
MDKLELVNDGLNIIDFIQKNQKEIQKTYGRSSIQQPSIKDQTKAWEDFLQCTSGESEQVEGG
MSKDDGDVERRNLEDLSSTSPTDGTIGKRVSNTRDWAEGSDDIQLDPVVTDVVYHDHGGECT
GYGFTSSPERGWSDYTSGANNGNVCLVSDAKMLSYAPEIAVSKEDRETDLVHLENKLSTTGL
NPTAVPFTLRNLSDPAKDSPVIAEHYYGLGVKEQNVGPQTSRNVNLDSIKLYTSDDEEADQL
EFEDEFAGSSSEVIVGISPEDEEPSSVGGKPNESIGRTIEGQSIRDNLQAKDNKSTDVPGAG
PKDSAVKEEPPQKRLPMLAEEFECSGSEDPIIRELLKENSLINCQQGKDAQPPYHWSIERSI
SPDKTEIVNGAVQTADRQRPGTPMPKSRGIPIKKGTDAKYPSAGTENVPGSKSGATRHVRGS
PPYQEGKSVNAENVQLNASTAVKETDKSEVNPVDDNDSLDDKYIMPSDDFSNTFFPHDTDRL
NYHADHLGDYDLETLCEESVLMGVINSIKLINLDMRLNHIEEQVKEIPKIINKLESIDRVLA
KTNTALSTIEGHLVSMMIM**I**PGKGKGERKGKNNPELKP**VI**GRD**I**LEQQS**L**FSFDNVKNFRDG
SLTNEPYGAAVQ**L**RED**LILP**E**L**N**F**EETN**A**SQ**FV**P**M**ADDSSRD**V**IKT**L**IRT**H**IKDRELRSELI
GYLNKAENDEEIQEIANTVNDI**I**DGNI

>NiV_P2
MDKLELVNDGLNIIDFIQKNQKEIQKTYGRSSIQQPSIKDQTKAWEDFLQCTSGESEQVEGG
MSKDDGDVERRNLEDLSSTSPTDGTIGKRVSNTRDWAEGSDDIQLDPVVTDVVYHDHGGECT
GYGFTSSPERGWSDYTSGANNGNVCLVSDAKMLSYAPEIAVSKEDRETDLVHLENKLSTTGL
NPTAVPFTLRNLSDPAKDSPVIAEHYYGLGVKEQNVGPQTSRNVNLDSIKLYTSDDEEADQL
EFEDEFAGSSSEVIVGISPEDEEPSSVGGKPNESIGRTIEGQSIRDNLQAKDNKSTDVPGAG
PKDSAVKEEPPQKRLPMLAEEFECSGSEDPIIRELLKENSLINCQQGKDAQPPYHWSIERSI
SPDKTEIVNGAVQTADRQRPGTPMPKSRGIPIKKGTDAKYPSAGTENVPGSKSGATRHVRGS
PPYQEGKSVNAENVQLNASTAVKETDKSEVNPVDDNDSLDDKYIMPSDDFSNTFFPHDTDRL
NYHADHLGDYDLETLCEESVLMGVINSIKLINLDMRLNHIEEQVKEIPKIINKLESIDRVLA
KTNTALSTIEGHLVSMMIMIPGKGKGERKGKNNPELKPVIGRDILEQQSLFSFDNVKNFRDG
SLTNEPYGAAVQLREDLILPELNFEETNASQFVPMADDSSRDVIKTLIRTHIKDRELRSELI
GYLNKAENDEEIQEIANTVNDIIDGNI

>NiV_P3 (BSA = 1176 Å^2)
MDKLELVNDGLNIIDFIQKNQKEIQKTYGRSSIQQPSIKDQTKAWEDFLQCTSGESEQVEGG
MSKDDGDVERRNLEDLSSTSPTDGTIGKRVSNTRDWAEGSDDIQLDPVVTDVVYHDHGGECT
GYGFTSSPERGWSDYTSGANNGNVCLVSDAKMLSYAPEIAVSKEDRETDLVHLENKLSTTGL
NPTAVPFTLRNLSDPAKDSPVIAEHYYGLGVKEQNVGPQTSRNVNLDSIKLYTSDDEEADQL
EFEDEFAGSSSEVIVGISPEDEEPSSVGGKPNESIGRTIEGQSIRDNLQAKDNKSTDVPGAG
PKDSAVKEEPPQKRLPMLAEEFECSGSEDPIIRELLKENSLINCQQGKDAQPPYHWSIERSI
SPDKTEIVNGAVQTADRQRPGTPMPKSRGIPIKKGTDAKYPSAGTENVPGSKSGATRHVRGS
PPYQEGKSVNAENVQLNASTAVKETDKSEVNPVDDNDSLDDKYIMPSDDFSNTFFPHDTDRL
NYHADHLGDYDLETLCEESVLMGVINSIKLINLDMRLNHIEEQVKEIPKIINKLESIDRVLA
KTNTALSTIE**GH**L**V**S**M**M**IMIPG**KGKGERK**G**KNN**P**E**L**KPVIGRDILEQQSLFSFDNVKNFRDG
SLTNEPYGAAVQLREDLILPELNFEETNASQFVPMADDSSRDVIKTLIRTHIKDRELRSELI
GYLNKAENDEEIQEIANTVNDIIDGNI

>NiV_P4 (BSA = 617 Å^2)
MDKLELVNDGLNIIDFIQKNQKEIQKTYGRSSIQQPSIKDQTKAWEDFLQCTSGESEQVEGG
MSKDDGDVERRNLEDLSSTSPTDGTIGKRVSNTRDWAEGSDDIQLDPVVTDVVYHDHGGECT
GYGFTSSPERGWSDYTSGANNGNVCLVSDAKMLSYAPEIAVSKEDRETDLVHLENKLSTTGL
NPTAVPFTLRNLSDPAKDSPVIAEHYYGLGVKEQNVGPQTSRNVNLDSIKLYTSDDEEADQL
EFEDEFAGSSSEVIVGISPEDEEPSSVGGKPNESIGRTIEGQSIRDNLQAKDNKSTDVPGAG
PKDSAVKEEPPQKRLPMLAEEFECSGSEDPIIRELLKENSLINCQQGKDAQPPYHWSIERSI
SPDKTEIVNGAVQTADRQRPGTPMPKSRGIPIKKGTDAKYPSAGTENVPGSKSGATRHVRGS
PPYQEGKSVNAENVQLNASTAVKETDKSEVNPVDDNDSLDDKYIMPSDDFSNTFFPHDTDRL
NYHADHLGDYDLETLCEESVLMGVINSIKLINLDMRLNHIEEQVKEIPKIINKLESIDRVLA
KTNTALSTIEGH**LV**S**MMIMIPG**K**G**KGERKGKNNPELKPVIGRDILEQQSLFSFDNVKNFRDG
SLTNEPYGAAVQLREDLILPELNFEETNASQFVPMADDSSRDVIKTLIRTHIKDRELRSELI
GYLNKAENDEEIQEIANTVNDIIDGNI

Total BSA = 3575 Å^2
>NDV_P1 (BSA = 1303 Å^2)
MATFTDAEIDELFETSGTVIDSIITAQGKPVETVGRSAIPQGKTKALSLAWEKHGNTNTPAA
QESAGEQDQHGQNQASNSNRATPEEGPHSSQAQAATQPQEDANESQLKTGASSSLLSMLDKL
SNKSSNAKKGPPQSPPQQALHSKGSPAVEQTQHGANQGRAQQETGHQAAPSPGPPGTGVNIA
FPGQRGVSPQSVGATQPAPQSGQNQGSTPASADHVQPPVDFVQAMMSMMEAISQRVSKIDYQ
LDLVLKQTSSIPTMRSEIQQLKTSVAVMEANLGMMK**I**LD**PGC**ANVSSLSDLRAVAKSHP**V**LI
AGPGDPSP**Y**VTQGGE**I**A**L**NK**L**SQ**P**VPHPSDLIKHATSGG**P**DIG**I**ERDTVR**AL**I**L**SR**P**MHPSS
SSKLLSKLDSAGSVEEIRKIKRLA**L**NG
>NDV_P2 (BSA = 366 Å^2)
MATFTDAEIDELFETSGTVIDSIITAQGKPVETVGRSAIPQGKTKALSLAWEKHGNTNTPAA
QESAGEQDQHGQNQASNSNRATPEEGPHSSQAQAATQPQEDANESQLKTGASSSLLSMLDKL
SNKSSNAKKGPPQSPPQQALHSKGSPAVEQTQHGANQGRAQQETGHQAAPSPGPPGTGVNIA
FPGQRGVSPQSVGATQPAPQSGQNQGSTPASADHVQPPVDFVQAMMSMMEAISQRVSKIDYQ
LDLVLKQTSSIPTMRSEIQQLKTSVAVMEANLGMMKI**L**DPGCANVSS**L**SD**L**RAV**A**KS**HP**VLI
AGPGDPSPYVTQGGEIALNKLSQPVPHPSDLIKHATSGGPDIGIERDTVRALILSRPMHPSS
SSKLLSKLDSAGSVEEIRKIKRLALNG
>NDV_P3 (BSA = 983 Å^2)
MATFTDAEIDELFETSGTVIDSIITAQGKPVETVGRSAIPQGKTKALSLAWEKHGNTNTPAA
QESAGEQDQHGQNQASNSNRATPEEGPHSSQAQAATQPQEDANESQLKTGASSSLLSMLDKL
SNKSSNAKKGPPQSPPQQALHSKGSPAVEQTQHGANQGRAQQETGHQAAPSPGPPGTGVNIA
FPGQRGVSPQSVGATQPAPQSGQNQGSTPASADHVQPPVDFVQAMMSMMEAISQRVSKIDYQ
LDLVLKQTSSIPTMRSEIQQLKTSV**AV**ME**A**NLG**MM**K**IL**D**P**G**CA**N**V**SS**L**SD**L**RAVAKSHPVLI
AGPGDPSPYVTQGGEIALNKLSQPVPHPSDLIKHATSGGPDIGIERDTVRALILSRPMHPSS
SSKLLSKLDSAGSVEEIRKIKRLALNG
>NDV_P4 (BSA = 923 Å^2)
MATFTDAEIDELFETSGTVIDSIITAQGKPVETVGRSAIPQGKTKALSLAWEKHGNTNTPAA
QESAGEQDQHGQNQASNSNRATPEEGPHSSQAQAATQPQEDANESQLKTGASSSLLSMLDKL
SNKSSNAKKGPPQSPPQQALHSKGSPAVEQTQHGANQGRAQQETGHQAAPSPGPPGTGVNIA
FPGQRGVSPQSVGATQPAPQSGQNQGSTPASADHVQPPVDFVQAMMSMMEAISQRVSKIDYQ
LDLVLKQTSSIPTMRSEIQQLKTSVAV**M**EAN**LG**M**M**K**IL**D**PGCA**N**V**SS**L**SD**L**RAVAKSHPVLI
AGPGDPSPYVTQGGEIALNKLSQPVPHPSDLIKHATSGGPDIGIERDTVRALILSRPMHPSS
SSKLLSKLDSAGSVEEIRKIKRLALNG

Total BSA = 4583 Å^2

>RSV_P1 (BSA = 427 Å^2)
MEKFAPEFHGEDANNRATKFLESIKGKFTSPKDPKKKDSIISVNSIDIEVTKESPITSNSTI
INPTNETDDTAGNKPNYQRKPLVSFKEDPTPSDNPFSKLYKETIETFDNNEEESSYSYEEIN
DQTNDNITARLDRIDEKLSEILGMLHTLVVASAGPTSARDGIRDAMIGLREEMIEKIRTEAL
MTNDR**L**EA**MA**R**L**RNEESEKMAKDTSDEVSLNPTSEKLNNLLEGNDSDNDLSLEDFKGENKYF
QG

>RSV_P2 (BSA = 187 Å^2)
MEKFAPEFHGEDANNRATKFLESIKGKFTSPKDPKKKDSIISVNSIDIEVTKESPITSNSTI
INPTNETDDTAGNKPNYQRKPLVSFKEDPTPSDNPFSKLYKETIETFDNNEEESSYSYEEIN
DQTNDNITARLDRIDEKLSEILGMLHTLVVASAGPTSARDGIRDAMIGLREEMIEKIRTEAL
MTNDRLEAMARLRNEESEKMAKDTSDEVSLNPTSEKLNNLLEGNDSDNDLSLEDFKGENKYF
QG

>RSV_P3 (BSA = 1526 Å^2)
MEKFAPEFHGEDANNRATKFLESIKGKFTSPKDPKKKDSIISVNSIDIEVTKESPITSNSTI
INPTNETDDTAGNKPNYQRKPLVSFKEDPTPSDNPFSKLYKETIETFDNNEEESSYSYEEIN
DQTNDNITARLDRIDEKLSEI**LGMLH**T**L**V**VA**S**AGP**TS**A**RD**G**IRDAM**IGL**REE**M**IEKIRTEAL
MTNDRLEAMARLRNEESEKMAKDTSDEVSLNPTSEKLNNLLEGNDSDNDLSLEDFKGENKYF
QG

>RSV_P4 (BSA = 2433 Å^2)
MEKFAPEFHGEDANNRATKFLESIKGKFTSPKDPKKKDSIISVNSIDIEVTKESPITSNSTI
INPTNETDDTAGNKPNYQRKPLVSFKEDPTPSDNPFSKLYKETIETFDNNEEESSYSYEEIN
DQTNDNITARLDRIDEKLSEILGM**LH**T**LVVA**S**AGP**TSARD**GI**RD**A**MIGLREE**M**IEK**I**RTEA**L**
MTNDRLE**A**MAR**L**RNEESEK**MA**KDTSDE**V**S**L**NPTSEK**L**NN**LL**E**G**NDSDND**L**S**L**ED**F**KGENKYF
QG

```
Total BSA = 3348 Å2
>EBOV_P1
MTTRTKGRGHTAATTQNDRMPGPELSGWISEQLMTGRIPVSDIFCDIENNPGLCYASQMQQT
KPNPKTRNSQTQTDPICNHSFEEVVQTLASLATVVQQQTIASESLEQRITSLENGLKPVYDM
AKTISSLNRVCAEMVAKYDLLVMTTGRATATAAATEAYWAEHGQPPPGPSLYEESAIRGKIE
SRDETVPQSVREAFNNLNSTTSLTEENFGKPDISAKDLRNIMYDHLPGFGTAFHQLVQVICK
LGKDSNSLDIIHAEFQASLAEGDSPQCALIQITKRVPIFQDAAPPVIHIRSRGDIPRACQKS
LRPVPPSPKIDRGWVCVFQLQDGKTLGLKI
>EBOV_P2 (BSA = 161 Å2)
MTTRTKGRGHTAATTQNDRMPGPELSGWISEQLMTGRIPVSDIFCDIENNPGLCYASQMQQT
KPNPKTRNSQTQTDPICNHSFEEVVQTLASLATVVQQQTIASESLEQRITSLENGLKPVYDM
AKTISSLNRVCAEMVAKYDLLVMTTGRATATAAATEAYWAEHGQPPPGPSLYEESAIRGKIE
SRDETVPQSVREAFNNLNSTTSLTEENFGKPDISAKDLRNIMYDHLPGFGTAFHQLVQVICK
LGKDSNSLDIIHAEFQASLAEGDSPQCALIQITKRVPIFQDAAPPVIHIRSRGDIPRACQKS
LRPVPPSPKIDRGWVCVFQLQDGKTLGLKI
>EBOV_P3 (BSA = 2093 Å2)
MTTRTKGRGHTAATTQNDRMPGPELSGWISEQLMTGRIPVSDIFCDIENNPGLCYASQMQQT
KPNPKTRNSQTQTDPICNHSFEEVVQTLASLATVVQQQTIASESLEQRITSLENGLKPVYDM
AKTISSLNRVCAEMVAKYDLLVMTTGRATATAAATEAYWAEHGQPPPGPSLYEESAIRGKIE
SRDETVPQSVREAFNNLNSTTSLTEENFGKPDISAKDLRNIMYDHLPGFGTAFHQLVQVICK
LGKDSNSLDIIHAEFQASLAEGDSPQCALIQITKRVPIFQDAAPPVIHIRSRGDIPRACQKS
LRPVPPSPKIDRGWVCVFQLQDGKTLGLKI
>EBOV_P4 (BSA = 1094 Å2)
MTTRTKGRGHTAATTQNDRMPGPELSGWISEQLMTGRIPVSDIFCDIENNPGLCYASQMQQT
KPNPKTRNSQTQTDPICNHSFEEVVQTLASLATVVQQQTIASESLEQRITSLENGLKPVYDM
AKTISSLNRVCAEMVAKYDLLVMTTGRATATAAATEAYWAEHGQPPPGPSLYEESAIRGKIE
SRDETVPQSVREAFNNLNSTTSLTEENFGKPDISAKDLRNIMYDHLPGFGTAFHQLVQVICK
LGKDSNSLDIIHAEFQASLAEGDSPQCALIQITKRVPIFQDAAPPVIHIRSRGDIPRACQKS
LRPVPPSPKIDRGWVCVFQLQDGKTLGLKI
```

Figure S12 | NiV, NDV, RSV, and EBOV P protein sequences and their L protein interacting residues.

Hydrophobic residues in P1 (yellow), P2 (cyan), P3 (magenta) and P4 (blue) monomers of the P teramers contacting the L proteins are colored.

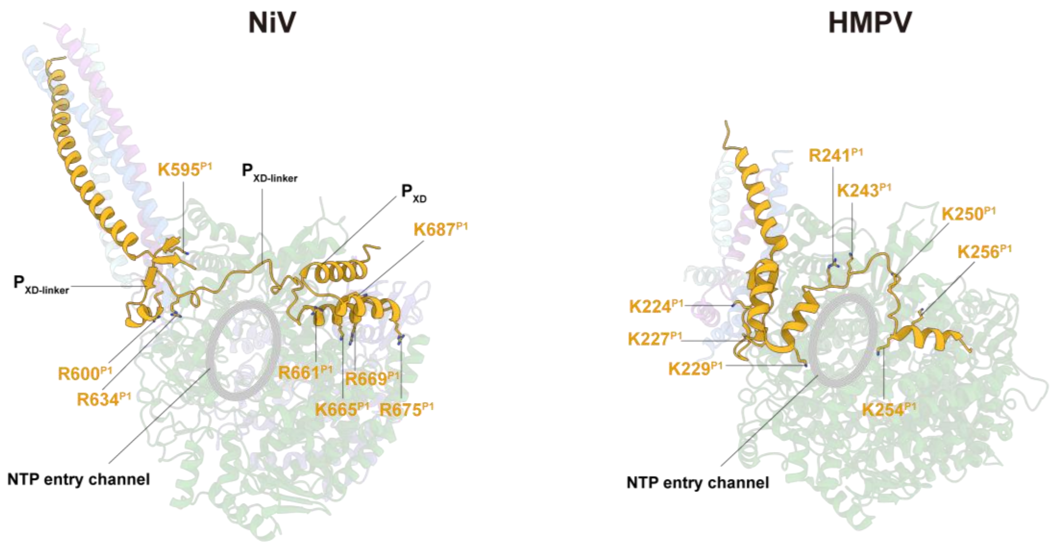

Figure S13 | The NiV P1$_{XD}$ domain and P1$_{XD}$ linker may play a potential role in NTP entry.

For HMPV, basic residues (K224^{P1}, K227^{P1}, K229^{P1}, R241^{P1}, K243^{P1}, K250^{P1}, K254^{P1} and K256^{P1}) in the C-terminal region of subunit P1, located around the polymerase NTP entry tunnel, form a positively charged arch that may attract NTPs to the NTP entry tunnel. Similarly, for NiV, we observe several basic amino acids (K595^{P1}, R600^{P1}, R634^{P1}, R661^{P1}, K665^{P1}, R669^{P1}, R675^{P1} and K687^{P1}) present in the P1$_{XD}$ domain and P1$_{XD}$ linker.

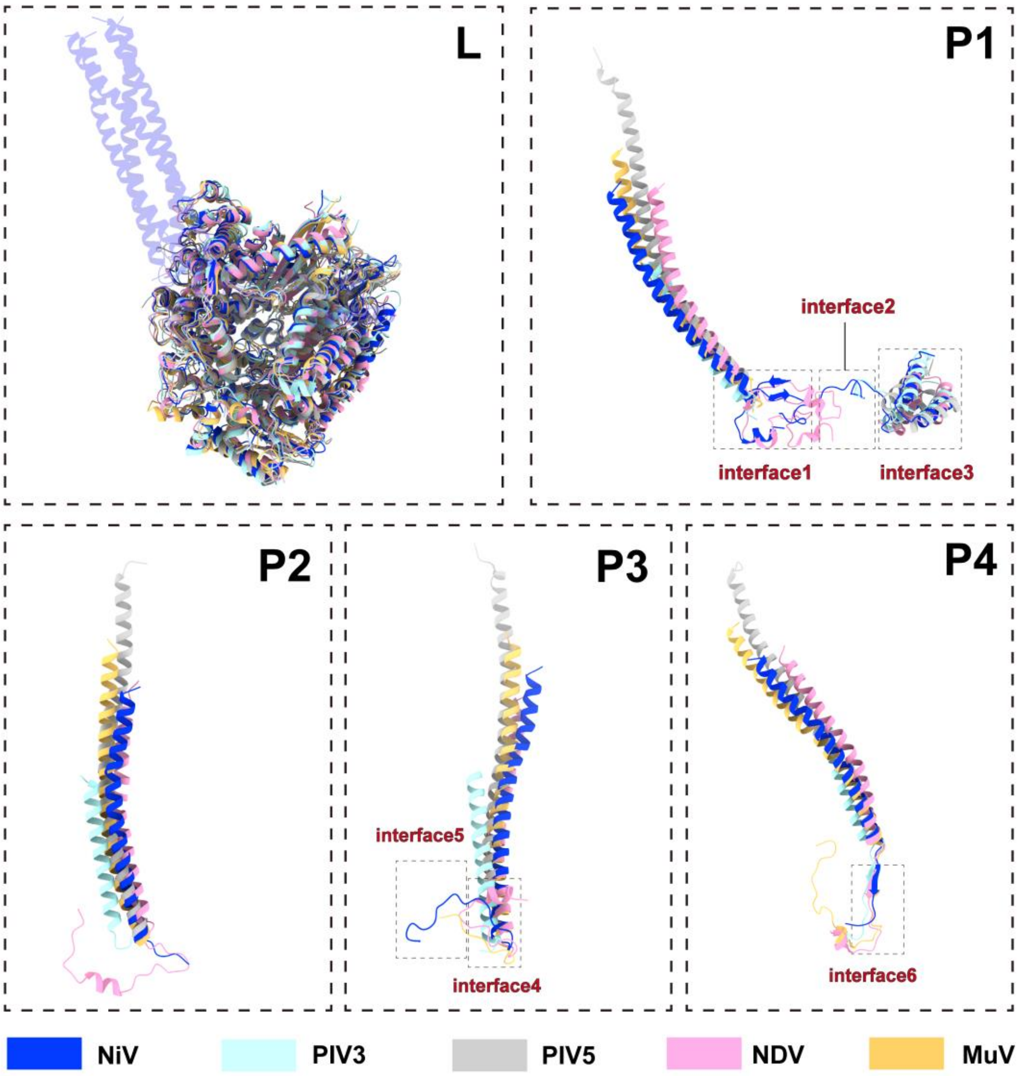

Figure S14 | Comparison of P protein strcutures among paramyxovirus L–P complexes.

Structural superposition of the paramyxoviruses L-P complexes reveals that P (P1, P2, P3 and P4) proteins from different viruses exhibit conformational dynamics on the surface of the L proteins (NiV: blue; PIV3: cyan, 8KDC; PIV5: gray, 6V85; NDV: pink, 7YOV; MuV: yellow, 8IZL).

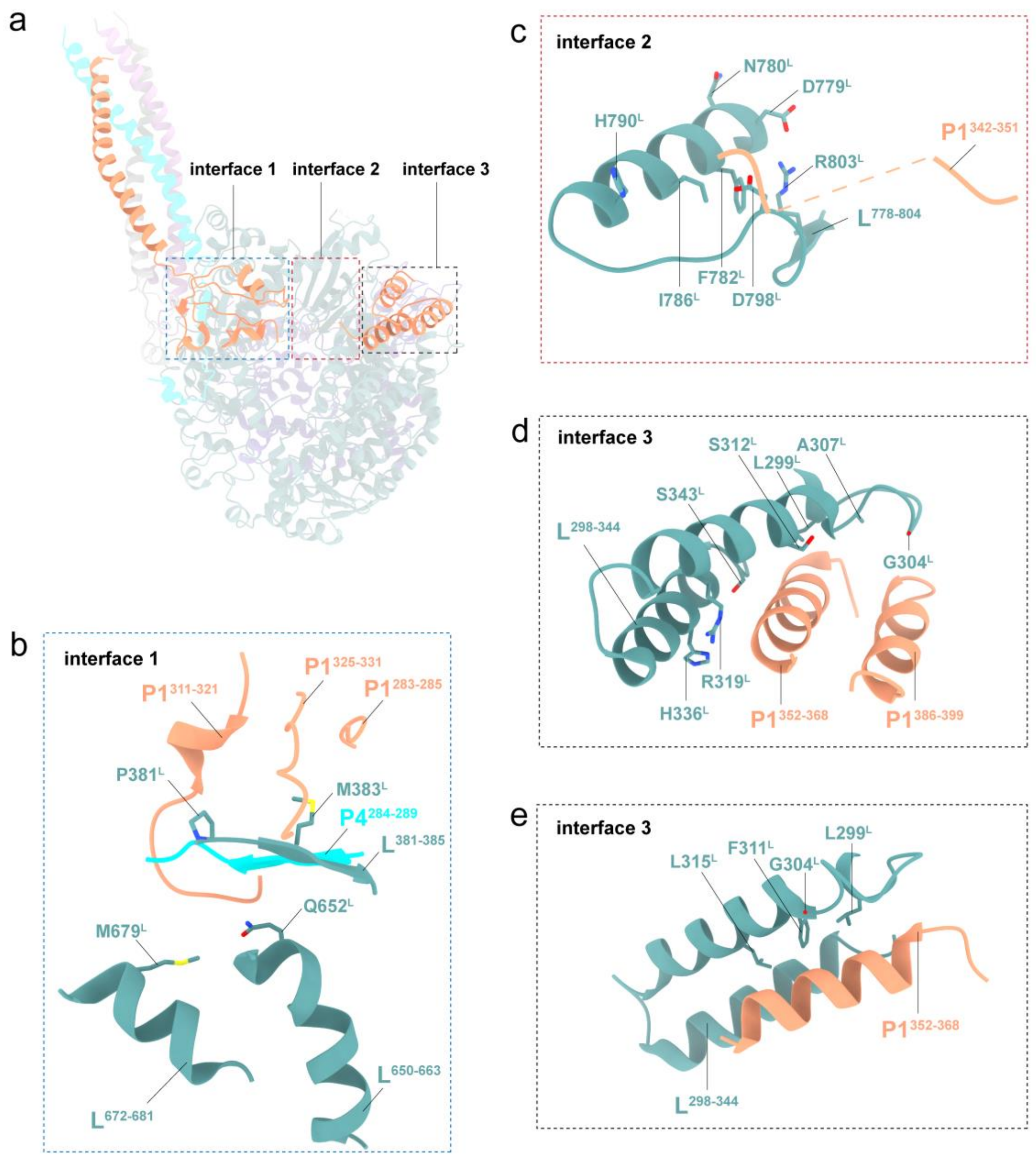

Figure S15 | Structural features of the L-P interactions in interfaces 1-3 of the NDV polymerase complex.

a. The overall structure of the NDV L-P complex (PDB: 7YOV). The locations of the interfaces 1-3, equivalent to those in the NiV L-P complex, are indicated by the dashed boxes. **b-e**. Strcurual details in the NDV L-P interfaces. The L residues in NDV, equivalent to those involved in the NiV

L-P interaction, are shown as sticks and labeled. These residues exhibit substantial variations when compared to those in NiV (see **Fig. 4**).

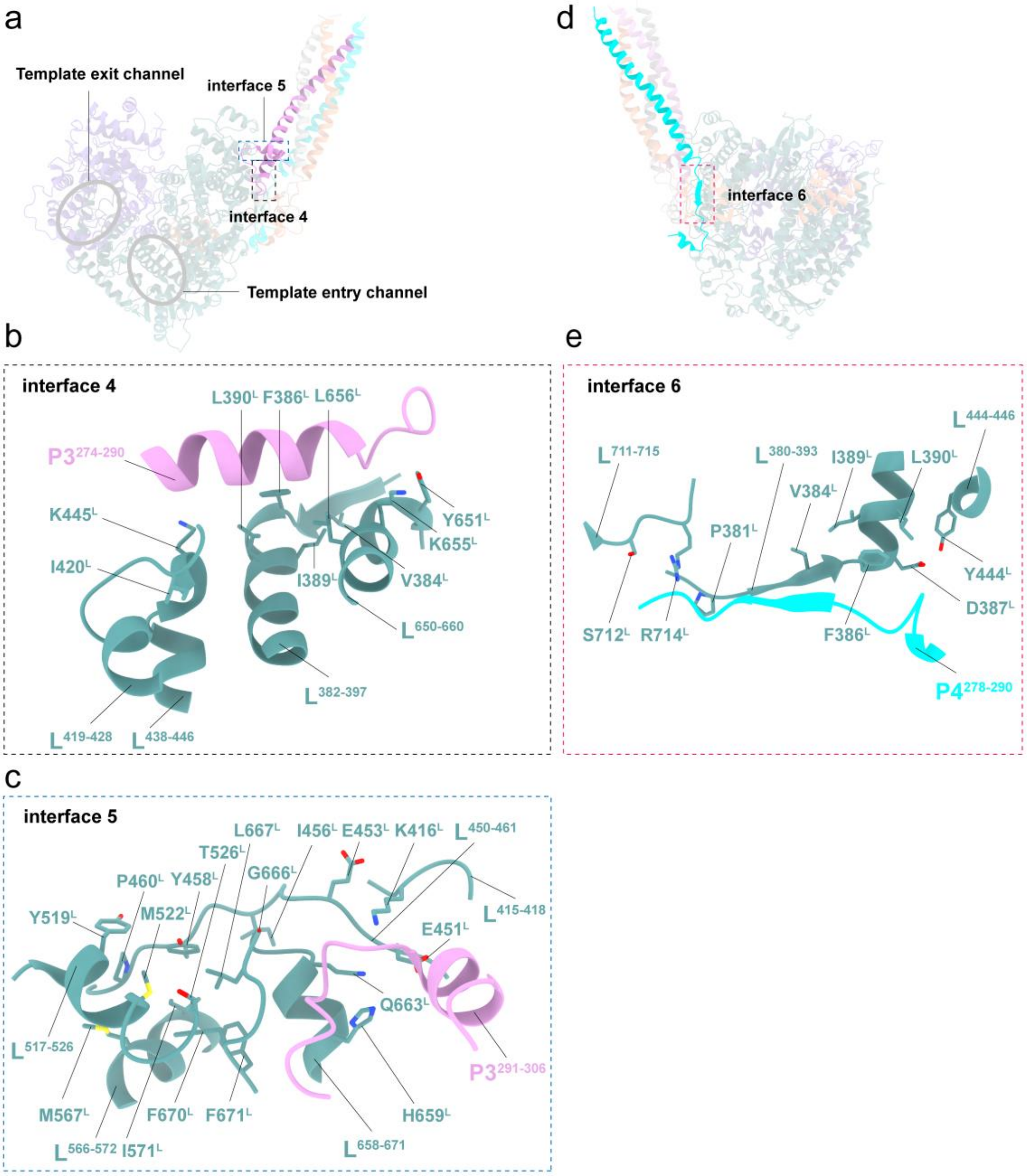

Figure S16 | Structural features of the L-P interactions in interfaces 4-6 of the NDV polymerase complex.

a. The overall structure of the NDV L-P complex (PDB: 7YOV). The locations of the interfaces 4 and 5, equivalent to those in the NiV L-P complex, are indicated by the dashed boxes. **d.** The overall structure of the

NDV L-P complex (PDB: 7YOV) in a different view. The location of the interfaces 6 is indicated by the dashed box. **b-c and e.** Strcurual details in the NDV L-P interfaces. The L residues in NDV, equivalent to those involved in the NiV L-P interaction, are shown as sticks and labeled. These residues exhibit substantial variations when compared to those in NiV (see **Fig. 5**).

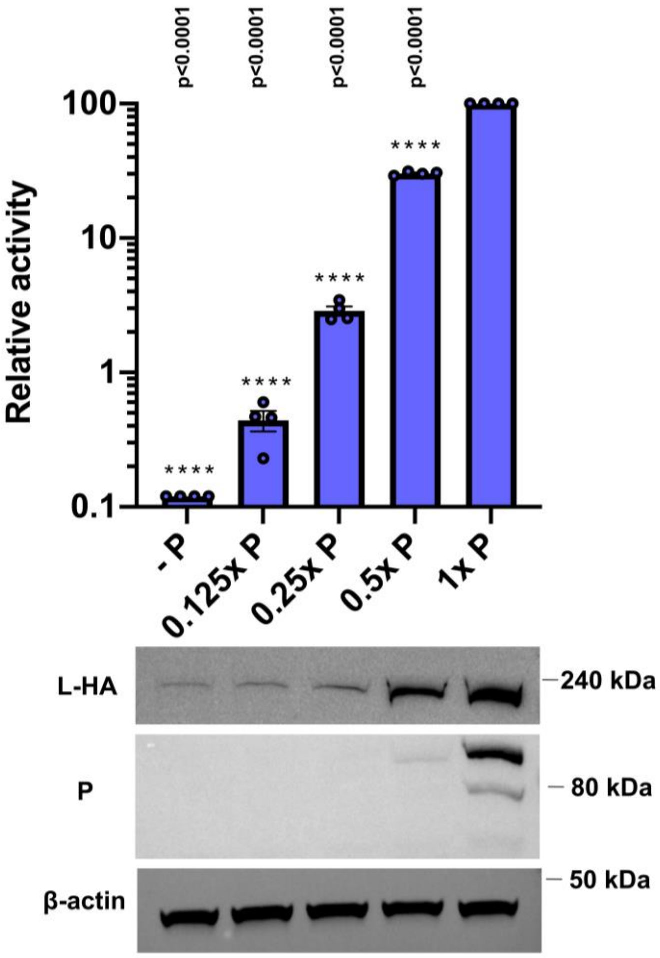

Figure S17 | P protein expression titration experiment showing P protein expression dependent L protein expression and mini-replicon activity.

1×P, 0.5×P, 0.25×P, 0.125×P and -P lanes represent protein expression levels in mini-replicon experiments, where 0.5μg, 0.25 μg, 0.125 μg, 0.0625 μg and 0 μg of P protein expression plasmid were transfected. Corresponding mini-replicon activities are reported as mean ± SEM from four independent experiments (n = 4). All statistics used one-way ANOVA

Dunnett's multiple comparisons test. (ns, $p > 0.05$; *, $p < 0.05$; **, $p < 0.01$; ***, $p < 0.001$; ****, $p < 0.0001$).

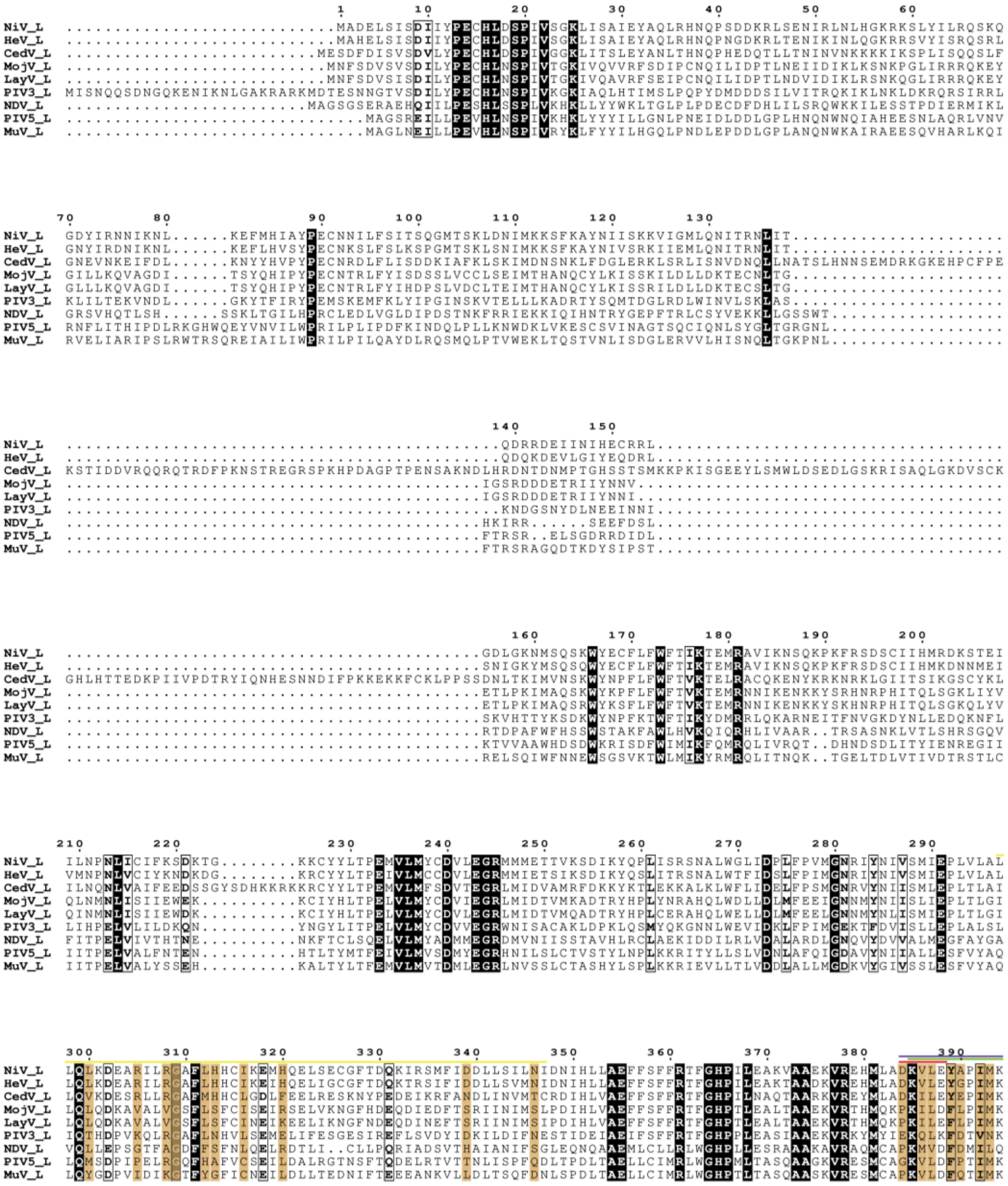
NiV_L
HeV_L
CedV_L
MojV_L
LayV_L
PIV3_L
NDV_L
PIV5_L
MuV_L

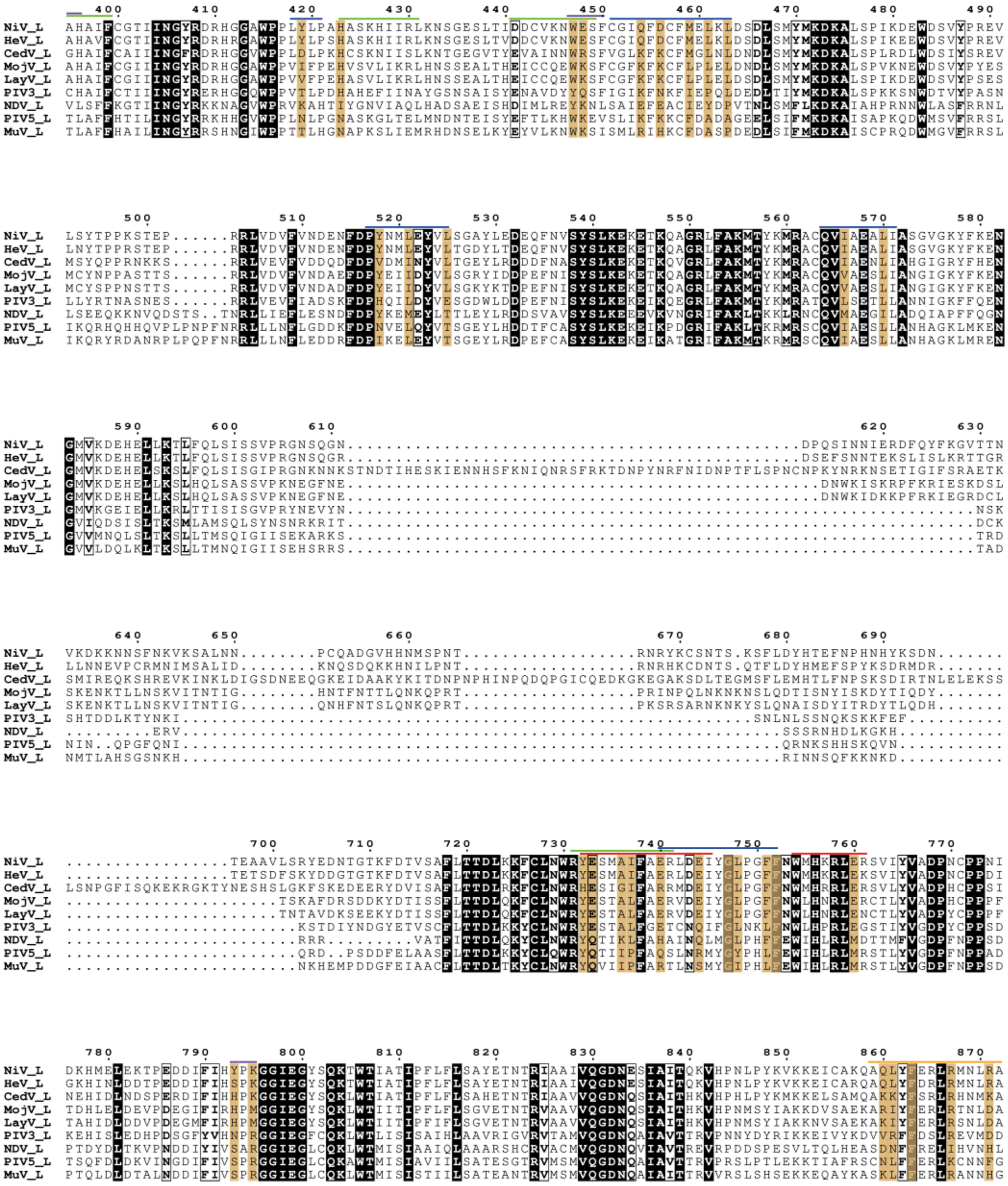

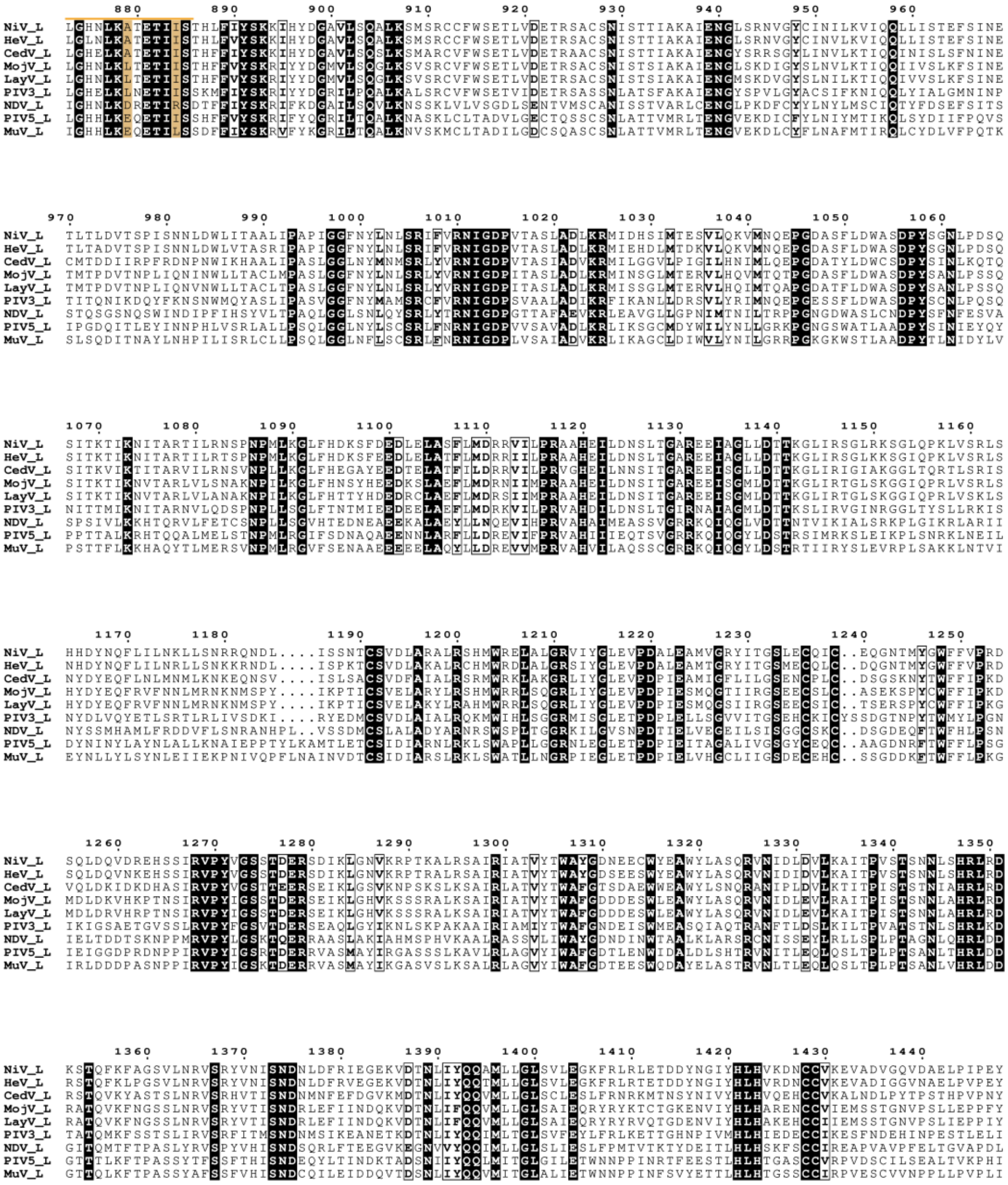

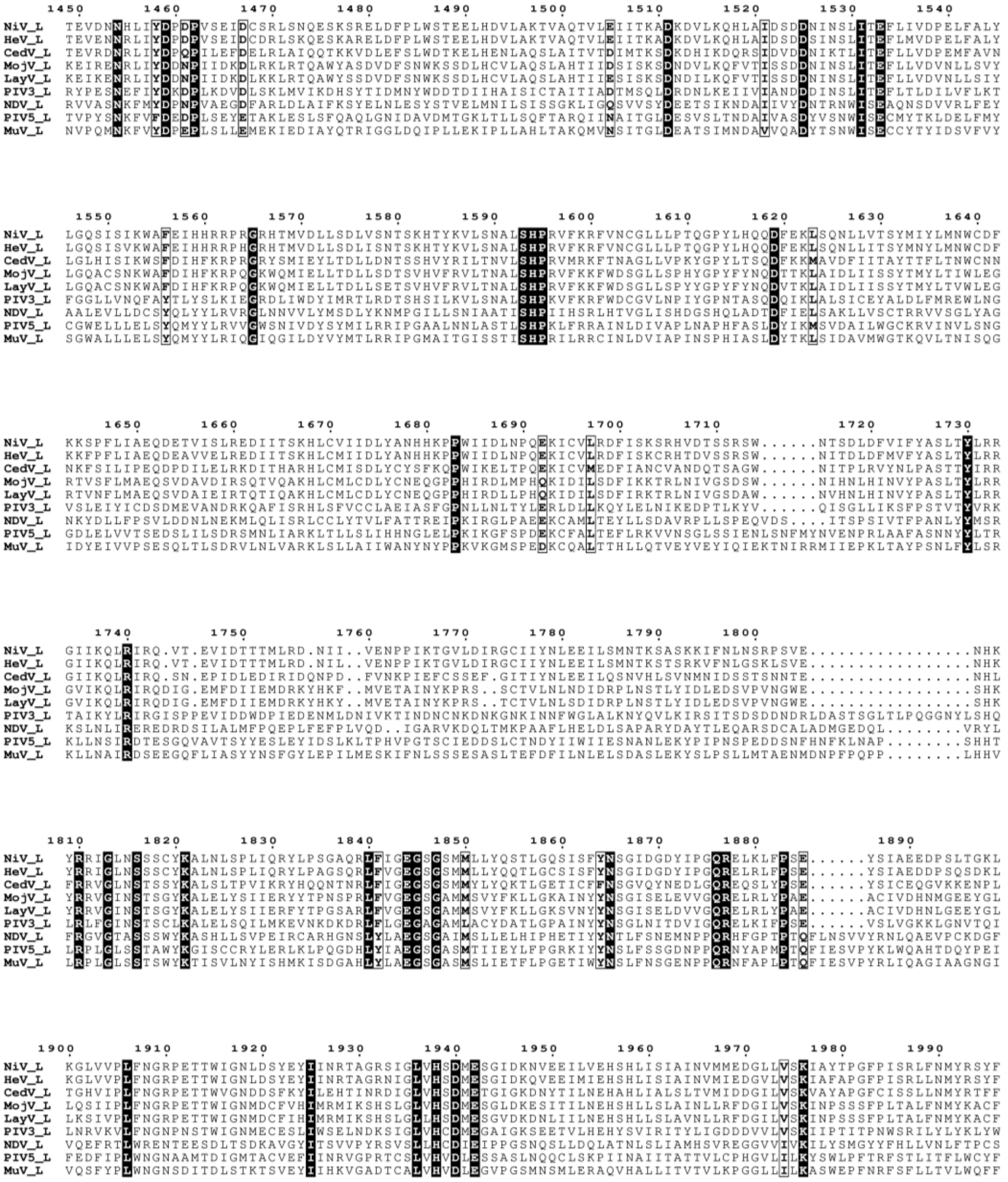

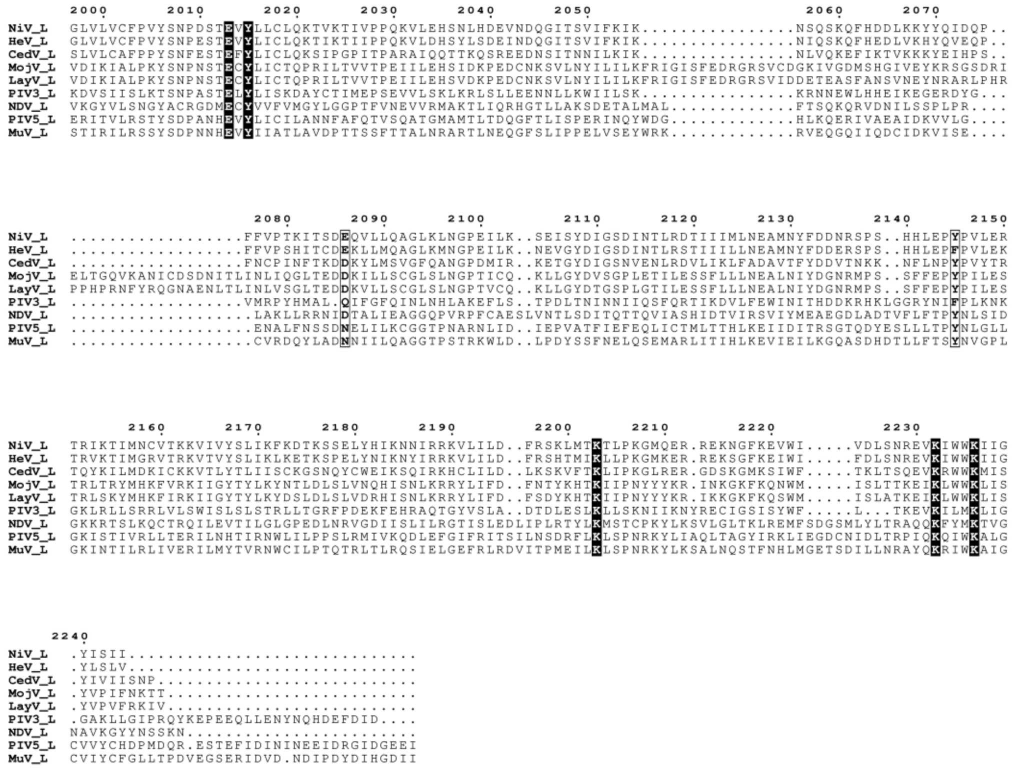

Figure S18 | Multiple sequence alignment of the L proteins from different paramyxovirus.

Residues in the NiV L protein interacting with the P proteins were identified. These residues and their equivalent residues in L proteins of the other paramyxoviruses are highlighted in the brown boxes. The NiV L sequences involved in interfaces 1, 2, 3, 4, 5 and 6 are indicated by red, orange, yellow, green, blue and purple overlines, respectively. Highly conserved amino acid residues are shaded black. L protein sequences used are NiV (Nipah virus), HeV (Hendra virus), CedV (Cedar virus), MojV

(Mojiang virus), LayV (Langya virus), PIV3 (Parainfluenza virus type 3), NDV (Newcastle disease virus), PIV5 (Parainfluenza virus 5), MuV (Mumps virus), EBOV (Ebola virus), RSV (Respiratory syncytial virus), HMPV (Human metapneumovirus), VSV (Vesicular stomatitis virus) and RABV (Rabies virus).

```
NiV_P1      MDKLELVNDGLNIIDFIQKNQKEIQKTYGRSSIQQPSIKDQTKAWEDFLQCTSGESEQVE    60
NDV_P1      ------------------------------------------------------------    0


NiV_P1      GGMSKDDGDVERRNLEDLSSTSPTDGTIGKRVSNTRDWAEGSDDIQLDPVVTDVVYHDHG    120
NDV_P1      ------------------------------------------------------------    0


NiV_P1      GECTGYGFTSSPERGWSDYTSGANNGNVCLVSDAKMLSYAPEIAVSKEDRETDLVHLENK    180
NDV_P1      ------------------------------------------------------------    0


NiV_P1      LSTTGLNPTAVPFTLRNLSDPAKDSPVIAEHYYGLGVKEQNVGPQTSRNVNLDSIKLYTS    240
NDV_P1      ------------------------------------------------------MATF--    4
                                                                 :  :

NiV_P1      DDEEADQLEFEDEFAGSSSEVIVGISPEDEEPSSVGGKPNESIGRTIEGQSIRDNLQ---    297
NDV_P1      TDAE-----IDELFE-TSGTVIDSII-------TAQGKPVETVGRSAIPQGKTKALSLAW    51
            * *     ::: *  :*. ** .*        :. *** *::**:   *.  . *.

NiV_P1      ------------------------AKDNKSTDVPGAGPKDSAVKEE-PPQKRLPMLAEEF    332
NDV_P1      EKHGNTNTPAAQESAGEQDQHGQNQASNSNRATPEEGPHSSQAQAATQPQEDAN---ESQ    108
                                     .*..  .*  **:.* .:    **:      *.

NiV_P1      ECSGSEDPIIRELLK-ENSLINCQQGKDAQPPYHWSIERSISPDKTEIVNGAVQTADRQR    391
NDV_P1      LKTGASSSLLSMLDKLSNKSSNAKKGPPQSPPQQ-ALHSKGSPAVEQTQHGANQGRAQQE    167
             :*:.. ::  * * .*.  *.::*   .** : ::. . **   :  :** *   :*.

NiV_P1      PGTPMPKSRGIPIKKGTDAKYPSAGTENVPGSKSGATRHVRGSPPYQEGKSVNAENVQLN    451
NDV_P1      TGHQAAPSPGPP-GTGVNIAFPG--QRGVSPQSVGATQP-----APQSGQNQG-------    212
            *     * * *  .*.:  :*.   ..*  .. ***:        *.*:. .

NiV_L      ASTAVKETDKSEVNPVDDNDSLDDKYIMPSDDFSNTFFPHDTDRLNYHADHLGDYDLETL     511
NDV_L      ------------STP------ASADHVQPPVDFVQAMMSMM-------------------     235
                        .*        . .:: *  ** ::::

NiV_P1      CEESVLMGVINSIKLINLDMRLNHIEEQVKEIPKIINKLESIDRVLAKTNTALSTIEGHL    571
NDV_P1      -------E-AISQRVSKIDYQLDLVLKQTSSIPTMRSEIQQLK-------TSVAVMEANL    280
                      * :: ::* :*: : :*...**.: .:::.:.       *:::.:*.:*

NiV_P1      VSMMIMIPGKGKGERK---------------GKNNPELKPVIGRDI----LEQQSLFSFD    612
NDV_P1      GMMKILDPGCANVSSLSDLRAVAKSHPVLIAGPGDPSPYVTQGGEIALNKLSQPVPHPSD    340
             * *: ** .: .                 * .:*.   . * :*    *.*   .  *

NiV_P1      NVKNFRDGSLTNEPYGAAVQLREDLILPELNFEETNASQFVPMADDSSRDVIKTLIRTHI    672
NDV_P1      LIKHATSG-------G-----------PDIGIE---------------RDTVRALILSRP    367
            :*:  .*       *           *::.:*               **.:::** ::

NiV_P1      KDRELRSELIGYLNKAENDEEIQEIANTVNDIIDGNI        709
NDV_P1      MHPSSSSKLLSKLDSAGSVEEIRKIKR---LALNG--        399
            . .  *:*:. *:.* . ***::* .     ::*
```

```
NiV_P2      MDKLELVNDGLNIIDFIQKNQKEIQKTYGRSSIQQPSIKDQTKAWEDFLQCTSGESEQVE     60
NDV_P2      ------------------------------------------------------------     0

NiV_P2      GGMSKDDGDVERRNLEDLSSTSPTDGTIGKRVSNTRDWAEGSDDIQLDPVVTDVVYHDHG     120
NDV_P2      ------------------------------------------------------------     0

NiV_P2      GECTGYGFTSSPERGWSDYTSGANNGNVCLVSDAKMLSYAPEIAVSKEDRETDLVHLENK     180
NDV_P2      ------------------------------------------------------------     0

NiV_P2      LSTTGLNPTAVPFTLRNLSDPAKDSPVIAEHYYGLGVKEQNVGPQTSRNVNLDSIKLYTS     240
NDV_P2      ------------------------------------------------------MATF--     4
                                                                  :  :

NiV_P2      DDEEADQLEFEDEFAGSSSEVIVGISPEDEEPSSVGGKPNESIGRTIEGQSIRDNLQ---     297
NDV_P2      TDAE-----IDELFE-TSGTVIDSII-------TAQGKPVETVGRSAIPQGKTKALSLAW     51
             * *     ::: *  :*. ** .*        :. *** *::**:   *.  . *.

NiV_P2      ------------------------AKDNKSTDVPGAGPKDSAVKEE-PPQKRLPMLAEEF     332
NDV_P2      EKHGNTNTPAAQESAGEQDQHGQNQASNSNRATPEEGPHSSQAQAATQPQEDAN---ESQ     108
                                      .*..  .*  **:.* .:    **:      *.

NiV_P2      ECSGSEDPIIRELLK-ENSLINCQQGKDAQPPYHWSIERSISPDKTEIVNGAVQTADRQR     391
NDV_P2      LKTGASSSLLSMLDKLSNKSSNAKKGPPQSPPQQ-ALHSKGSPAVEQTQHGANQGRAQQE     167
              :*:.. ::  * * .*.  *.::*   .** : ::. . **   :  :** *   :*.

NiV_P2      PGTPMPKSRGIPIKKGTDAKYPSAGTENVPGSKSGATRHVRGSPPYQEGKSVNAENVQLN     451
NDV_P2      TGHQAAPSPGPP-GTGVNIAFPG--QRGVSPQSVGATQP-----APQSGQNQG-------     212
             *     * * *  .*.:  :*.   ..*  .. ***:        *.*:. .

NiV_P2      ASTAVKETDKSEVNPVDDNDSLDDKYIMPSDDFSNTFFPHDTDRLNYHADHLGDYDLETL     511
NDV_P2      ------------STP------ASADHVQPPVDFVQAMMSMM-------------------     235
                         .*       . .:: *  ** ::::

NiV_P2      CEESVLMGVINSIKLINLDMRLNHIEEQVKEIPKIINKLESIDRVLAKTNTALSTIEGHL     571
NDV_P2      -------E-AISQRVSKIDYQLDLVLKQTSSIPTMRSEIQQLK-------TSVAVMEANL     280
                       * :: ::* :*: : :*...**.: .:::.:.       *:::.:*.:*

NiV_P2      VSMMIMIPGKGKGERK---------------GKNNPELKPVIGRDI----LEQQSLFSFD     612
NDV_P2      GMMKILDPGCANVSSLSDLRAVAKSHPVLIAGPGDPSPYVTQGGEIALNKLSQPVPHPSD     340
              * *: ** .: .                 * .:*.   . * :*    *.*   .  *

NiV_P2      NVKNFRDGSLTNEPYGAAVQLREDLILPELNFEETNASQFVPMADDSSRDVIKTLIRTHI     672
NDV_P2      LIKHATSG-------G-----------PDIGIE---------------RDTVRALILSRP     367
             :*:  .*       *           *::.:*               **.:::** ::

NiV_P2      KDRELRSELIGYLNKAENDEEIQEIANTVNDIIDGNI        709
NDV_P2      MHPSSSSKLLSKLDSAGSVEEIRKIKR---LALNG--        399
             . .  *:*:. *:.* . ***::* .     ::*
```

```
NiV_P3      MDKLELVNDGLNIIDFIQKNQKEIQKTYGRSSIQQPSIKDQTKAWEDFLQCTSGESEQVE    60
NDV_P3      ------------------------------------------------------------    0


NiV_P3      GGMSKDDGDVERRNLEDLSSTSPTDGTIGKRVSNTRDWAEGSDDIQLDPVVTDVVYHDHG    120
NDV_P3      ------------------------------------------------------------    0


NiV_P3      GECTGYGFTSSPERGWSDYTSGANNGNVCLVSDAKMLSYAPEIAVSKEDRETDLVHLENK    180
NDV_P3      ------------------------------------------------------------    0


NiV_P3      LSTTGLNPTAVPFTLRNLSDPAKDSPVIAEHYYGLGVKEQNVGPQTSRNVNLDSIKLYTS    240
NDV_P3      ------------------------------------------------------MATF--    4
                                                                  :  :

NiV_P3      DDEEADQLEFEDEFAGSSSEVIVGISPEDEEPSSVGGKPNESIGRTIEGQSIRDNLQ---    297
NDV_P3      TDAE-----IDELFE-TSGTVIDSII-------TAQGKPVETVGRSAIPQGKTKALSLAW    51
             * *     ::: *  :*. ** .*        :. *** *::**:   *.  . *.

NiV_P3      ------------------------AKDNKSTDVPGAGPKDSAVKEE-PPQKRLPMLAEEF    332
NDV_P3      EKHGNTNTPAAQESAGEQDQHGQNQASNSNRATPEEGPHSSQAQAATQPQEDAN---ESQ    108
                                      .*..  .*  **:.* .:    **:      *.

NiV_P3      ECSGSEDPIIRELLK-ENSLINCQQGKDAQPPYHWSIERSISPDKTEIVNGAVQTADRQR    391
NDV_P3      LKTGASSSLLSMLDKLSNKSSNAKKGPPQSPPQQ-ALHSKGSPAVEQTQHGANQGRAQQE    167
              :*:.. ::  * * .*.  *.::*   .** : ::. . **   :  :** *   :*.

NiV_P3      PGTPMPKSRGIPIKKGTDAKYPSAGTENVPGSKSGATRHVRGSPPYQEGKSVNAENVQLN    451
NDV_P3      TGHQAAPSPGPP-GTGVNIAFPG--QRGVSPQSVGATQP-----APQSGQNQG-------    212
             *     * * *  .*.:  :*.   ..*  .. ***:        *.*:. .

NiV_P3      ASTAVKETDKSEVNPVDDNDSLDDKYIMPSDDFSNTFFPHDTDRLNYHADHLGDYDLETL    511
NDV_P3      ------------STP------ASADHVQPPVDFVQAMMSMM-------------------    235
                         .*       . .:: *  ** ::::

NiV_P3      CEESVLMGVINSIKLINLDMRLNHIEEQVKEIPKIINKLESIDRVLAKTNTALSTIEGHL    571
NDV_P3      -------E-AISQRVSKIDYQLDLVLKQTSSIPTMRSEIQQLK-------TSVAVMEANL    280
                       * :: ::* :*: : :*...**.: .:::.:.       *:::.:*.:*

NiV_P3      VSMMIMIPGKGKGERK---------------GKNNPELKPVIGRDI----LEQQSLFSFD    612
NDV_P3      GMMKILDPGCANVSSLSDLRAVAKSHPVLIAGPGDPSPYVTQGGEIALNKLSQPVPHPSD    340
              * *: ** .: .                 * .:*.   . * :*    *.*   .  *

NiV_P3      NVKNFRDGSLTNEPYGAAVQLREDLILPELNFEETNASQFVPMADDSSRDVIKTLIRTHI    672
NDV_P3      LIKHATSG-------G-----------PDIGIE---------------RDTVRALILSRP    367
             :*:  .*       *           *::.:*               **.:::** ::

NiV_P3      KDRELRSELIGYLNKAENDEEIQEIANTVNDIIDGNI        709
NDV_P3      MHPSSSSKLLSKLDSAGSVEEIRKIKR---LALNG--        399
             . .  *:*:. *:.* . ***::* .     ::*
```

```
NiV_P4      MDKLELVNDGLNIIDFIQKNQKEIQKTYGRSSIQQPSIKDQTKAWEDFLQCTSGESEQVE    60
NDV_P4      ------------------------------------------------------------    0


NiV_P4      GGMSKDDGDVERRNLEDLSSTSPTDGTIGKRVSNTRDWAEGSDDIQLDPVVTDVVYHDHG    120
NDV_P4      ------------------------------------------------------------    0


NiV_P4      GECTGYGFTSSPERGWSDYTSGANNGNVCLVSDAKMLSYAPEIAVSKEDRETDLVHLENK    180
NDV_P4      ------------------------------------------------------------    0


NiV_P4      LSTTGLNPTAVPFTLRNLSDPAKDSPVIAEHYYGLGVKEQNVGPQTSRNVNLDSIKLYTS    240
NDV_P4      ------------------------------------------------------MATF--    4
                                                                 :  :

NiV_P4      DDEEADQLEFEDEFAGSSSEVIVGISPEDEEPSSVGGKPNESIGRTIEGQSIRDNLQ---    297
NDV_P4      TDAE-----IDELFE-TSGTVIDSII-------TAQGKPVETVGRSAIPQGKTKALSLAW    51
            * *     ::: *  :*. ** .*        :. *** *::**:   *.  . *.

NiV_P4      -------------------------AKDNKSTDVPGAGPKDSAVKEE-PPQKRLPMLAEEF    332
NDV_P4      EKHGNTNTPAAQESAGEQDQHGQNQASNSNRATPEEGPHSSQAQAATQPQEDAN---ESQ    108
                                     .*..  .*  **:.* .:    **:      *.

NiV_P4      ECSGSEDPIIRELLK-ENSLINCQQGKDAQPPYHWSIERSISPDKTEIVNGAVQTADRQR    391
NDV_P4      LKTGASSSLLSMLDKLSNKSSNAKKGPPQSPPQQ-ALHSKGSPAVEQTQHGANQGRAQQE    167
             :*:.. ::  * * .*.  *.::*   .** : ::. . **   :  :** *   :*.

NiV_P4      PGTPMPKSRGIPIKKGTDAKYPSAGTENVPGSKSGATRHVRGSPPYQEGKSVNAENVQLN    451
NDV_P4      TGHQAAPSPGPP-GTGVNIAFPG--QRGVSPQSVGATQP-----APQSGQNQG-------    212
            *     * * *  .*.:  :*.   ..*  ..  ***:        *.*:. .

NiV_P4      ASTAVKETDKSEVNPVDDNDSLDDKYIMPSDDFSNTFFPHDTDRLNYHADHLGDYDLETL    511
NDV_P4      ------------STP------ASADHVQPPVDFVQAMMSMM-------------------    235
                        .*       . .:: *  ** ::::

NiV_P4      CEESVLMGVINSIKLINLDMRLNHIEEQVKEIPKIINKLESIDRVLAKTNTALSTIEGHL    571
NDV_P4      -------E-AISQRVSKIDYQLDLVLKQTSSIPTMRSEIQQLK-------TSVAVMEANL    280
                      * :: ::* :*: : :*...**.: .:::.:.       *:::.:*.:*

NiV_P4      VSMMIMIPGKGKGERK---------------GKNNPELKPVIGRDI----LEQQSLFSFD    612
NDV_P4      GMMKILDPGCANVSSLSDLRAVAKSHPVLIAGPGDPSPYVTQGGEIALNKLSQPVPHPSD    340
             * *: ** .: .                 *  .:*.   . * :*    *.*   .  *

NiV_P4      NVKNFRDGSLTNEPYGAAVQLREDLILPELNFEETNASQFVPMADDSSRDVIKTLIRTHI    672
NDV_P4      LIKHATSG-------G-----------PDIGIE---------------RDTVRALILSRP    367
            :*:  .*       *           *::.:*               **.:::** ::

NiV_P4      KDRELRSELIGYLNKAENDEEIQEIANTVNDIIDGNI        709
NDV_P4      MHPSSSSKLLSKLDSAGSVEEIRKIKR---LALNG--        399
            . .  *:*:. *:.* . ***::* .     ::*
```

Figure S19 | Hydrophobic residues in P1 (yellow), P2 (cyan), P3 (magenta) and P4 (blue) monomers contacting the L proteins are compared between NiV and NDV.

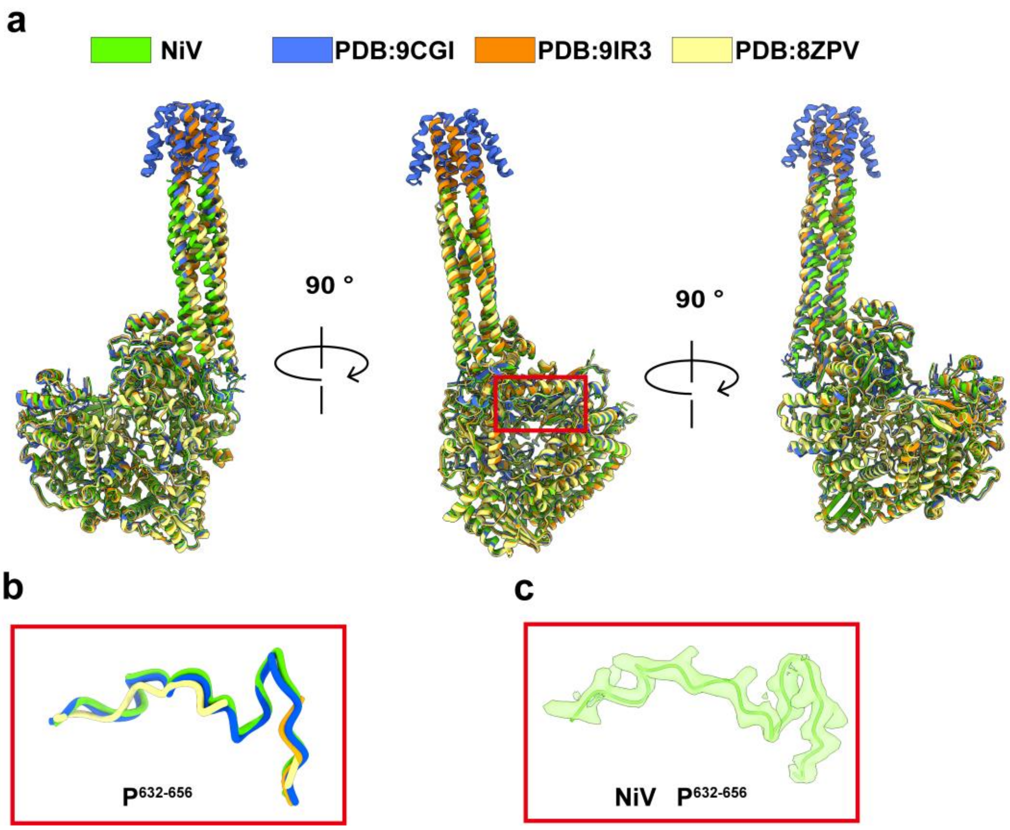

Figure S20 | A comparison of the reported NiV L-P complex structures.

a. Three different views of our NiV $L_{1\text{-}1451}$-P complex structure superposed onto the released NiV L-P complex structures (9CGI, 9IR3 and 8ZPV). **b.** Close-up view of the modelled structures for the residues $P^{632\text{-}656}$. **c.** The cryo-EM density for the residues $P^{632\text{-}656}$ in the NiV $L_{1\text{-}1451}$-P complex structure.

Supplementary Table 1. Cryo-EM data collection, refinement and validation statistics.

	NiV $L_{1\text{-}1451}$-P complex	NiV L-P complex
**Data collection and processing**		
Magnification	165000	165000
Voltage (kV)	300	300
Electron exposure ($e^-/Å^2$)	50	50
Defocus range (μm)	0.6-2.4	0.6-2.4
Pixel size (Å)	0.73	0.73
Movies (no.)	16735	10073
Initial particle images (no.)	4390986	7475406
Symmetry imposed	*C1*	*C1*
Final particle images (no.)	674945	224424
Map resolution (Å)	2.31	2.52
FSC threshold	0.143	0.143
Map resolution range (Å)	2.30-37.23	2.15-38.17
**Refinement**		
Initial model used	PDB 6EB8	PDB 9IV9
Model resolution (Å)	2.56	2.87
FSC threshold	0.5	0.5
Map sharpening *B* factor ($Å^2$)	-88	-89
Model composition		
Non-hydrogen atoms	13048	12944
Protein residues	1624	1611
Ligands	2	2
B factors ($Å^2$)		
Protein	104.83	101.08
Ligand	146.19	124.50
R.m.s. deviations		
Bond lengths (Å)	0.004	0.004
Bond angles (°)	0.798	0.669
**Validation**		
MolProbity score	1.34	1.34
Clash score	3.69	3.45
Poor rotamers (%)	0	0
Ramachandran plot		
Favored (%)	96.94	96.73
Allowed (%)	3.06	3.27
Disallowed (%)	0	0

Supplementary Table 2. Interactions between L and P proteins.

Interface	Molecule	Sites	L	Interaction Pattern
Interface1	P1	I576^{P1}<br>I578^{P1}<br>L608^{P1}<br>L633^{P1}<br>L637^{P1}<br>I638^{P1}	V386^{L}	Hydrophobic interactions
		R634^{P1}	D384^{L}	long-range electrostatic interaction
		R600^{P1}	E733^{L}<br>E760^{L}	salt bridges
	P4	M575^{P4}<br>M577^{P4}<br>P579^{P4}	V386^{L}	Hydrophobic interactions
Interface2	P1	L639^{P1}#	R871^{L}	hydrogen bond
		P640^{P1}#	R867^{L}	hydrogen bond
		Q651^{P1}	Q860^{L}	hydrogen bond
		L642^{P1}<br>F644^{P1}	L861^{L}<br>F863^{L}<br>A879^{L}<br>I884^{L}	Hydrophobic interactions
Interface3	P1	D658^{P1}#<br>D662^{P1}	H320^{L}	hydrogen bond<br>salt bridge
		S660^{P1}	H313^{L}	hydrogen bond
		K665^{P1}<br>R669^{P1}	D339^{L}	salt bridges
		T670^{P1}	N346^{L}	hydrogen bond
		H671^{P1}	L300^{L}#	hydrogen bond
		N702^{P1}<br>D706^{P1}	R308^{L}	hydrogen bond<br>salt bridge
		D706^{P1}<br>D703^{P1}	R305^{L}	salt bridges
		F652^{P1}<br>V663^{P1}<br>L667^{P1}	L300^{L}<br>G309^{L}<br>L312^{L}<br>I316^{L}	Hydrophobic interactions
interface4	P3	S565^{P3}	H423^{L}	hydrogen bond
		H570^{P3}	Y389^{L}<br>E448^{L}	hydrogen bond<br>salt bridge
		I576^{P3}<br>M577^{P3}<br>P579^{P3}	L387^{L}<br>I392^{L}<br>M393^{L}<br>Y732^{L}<br>A736^{L}<br>I737^{L}	Hydrophobic interactions
Interface5	P3	K583^{P3}	E740^{L}<br>E744^{L}	salt bridges
		E585^{P3}#	Q454^{L}	hydrogen bond
		K587^{P3}	Y419^{L}	cation-π interaction
		K589^{P3}#	D456^{L}	salt bridge
		N591^{P3}	M459^{L}#<br>Y746^{L}#	hydrogen bonds

		E593^{P3}	L461^{L}#<br>Y518^{L}	hydrogen bonds
		L594^{P3}	L461^{L}<br>L463^{L}<br>Y518^{L}<br>L521^{L}<br>L525^{L}<br>I566^{L}<br>L570^{L}<br>L748^{L}<br>F751^{L}<br>F752^{L}	Hydrophobic interactions
Interface6	P4	G580^{P4}#	D384^{L}	hydrogen bond
		G582^{P4}#	K795^{L}	hydrogen bond
		K583^{P4}	Y793^{L}	cation-π interaction
		V572^{P4}<br>M574^{P4}<br>I576^{P4}<br>I578^{P4}	L387^{L}<br>Y389^{L}<br>A390^{L}<br>I392^{L}<br>M393^{L}<br>W447^{L}	Hydrophobic interactions

The "#" symbol indicates the formation of hydrogen bonds with nitrogen or oxygen in the main chain.
